# Mechanochemical Methods for Amide Bond Formation

**DOI:** 10.1021/acs.chemrev.5c00534

**Published:** 2026-05-20

**Authors:** Tatsiana Nikonovich, Christos M. Chatzigiannis, Corentin Bordier, Rubén Solorzano-Rodriguez, Daniel M. Baier, Florian F. Ort, Riina Aav, Tom Leyssens, Daniel Blanco-Ania, Floris P. J. T. Rutjes, Dzmitry Kananovich, Evelina Colacino

**Affiliations:** ∇ Department of Chemistry and Biotechnology, 54561 Tallinn University of Technology, Akadeemia tee 15, 12618 Tallinn, Estonia; ‡ 119128 ICGM, Univ Montpellier, CNRS, ENSCM, 34293 Montpellier, France; § IMCN/MOST, UCLouvain, 1 Place Louis Pasteur, 1348 Louvain-La-Neuve, Belgium; ∥ Synthetic Organic Chemistry, IMM, 6029 Radboud University, Heyendaalseweg 135, 6525 AJ, Nijmegen, The Netherlands

## Abstract

Mechanochemical methodologies are reshaping synthetic organic chemistry by enhancing practicality and reducing environmental impact. This review presents a comprehensive account of mechanochemical methods for amide bond formation, arguably the most developed and industrially relevant area within mechanochemical organic synthesis. Covering literature from early contributions to the present (September 2025), the review organization follows key substrate classes and methodological strategies: amide bond formation via coupling of carboxylic acids or their activated derivatives with amines (Section 2), followed by unconventional approaches (Section 3) employing carboxylic acid and amine surrogates, redox chemistry, rearrangements, and transition metal-mediated reactions leading to amide products through alternative bond-forming pathways. Mechanoenzymatic transformations are treated separately (Section 4), with stereochemistry-related issues, such as the preservation of enantiomeric purity, discussed in Section 5. Section 6 highlights the use of amide bond formation as a model system for probing mechanochemical driving forces. Special attention is given to scalability and successful scale-up examples, alignment with green chemistry principles, limitations, unexplored areas, and challenges requiring further development. This review is intended as an accessible and thorough resource for synthetic chemists in both academia and industry, including those newly exploring the field of mechanochemistry, and it provides practical guidance for process optimization and scale-up.

## Graphical abstract

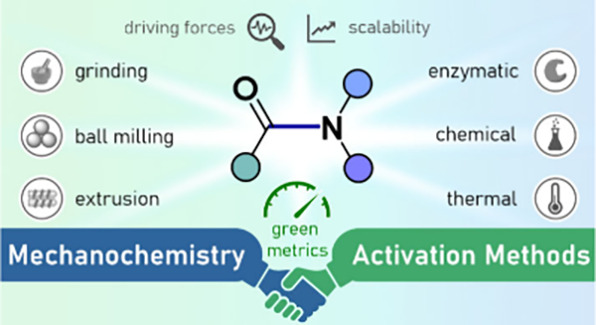

## 1 Introduction

Amides constitute a vital class of organic compounds, with the peptide (amide) bond forming the backbone of essential biomolecules such as peptides and proteins. This bond also serves as a fundamental structural element in pharmaceuticals, agrochemicals, polymers, and advanced materials. Amide bond formation is one of the most common transformations in drug discovery and development, accounting for approximately a quarter of all reactions used in the field.^1^ In recent years, the growing importance of therapeutic peptides^2^ for the treatment of various diseases has further increased the relevance of peptide coupling strategies. Consequently, this area has drawn considerable interest, with researchers focused on developing more efficient and versatile methods for synthesizing amides and peptides. Efforts have been directed toward optimizing stoichiometric activating agents,^3,4^ use of green solvents,^5,6^ establishing new approaches and advancing catalytic systems,^7–9^ and exploring photochemical,^10,11^ electrochemical,^12^ biocatalytic,^13^ and flow chemistry^14^ approaches.

However, conventional organic synthesis depends on performing reactions in organic solvents, which account for 80–90% of total mass input and are major contributors to process safety concerns,^15^ waste generation, and environmental impact, particularly in multistep peptide synthesis.^16^ Underscoring the urgency of the problem, the two most widely used solvents for amide bond formation, DMF and DCM,^17^ have recently faced regulatory restrictions in both the EU^18,19^ and the USA^20,21^ due to their hazardous nature.^18,20^ In response to these challenges, the development of greener and more sustainable amidation methods remains a central focus of initiatives such as the Green Chemistry Challenge by the American Chemical Society Green Chemistry Institute Pharmaceutical Roundtable (ACS GCIPR).^22^

In this context, mechanochemistry has emerged as a powerful enabling technology for sustainable organic synthesis,^23–27^ as it induces chemical transformations in solid reactants through the action of mechanical force (typically applied through ball milling or extrusion). By conducting reactions in the solid state or with minimal liquid additives (liquid-assisted grinding, LAG),^300,28^ mechanochemistry circumvents the need for bulk solvents, potentially leading to intensified processes, reduced waste,^29^ enhanced safety, shorter reaction times, and sometimes unique reactivity or selectivity profiles unattainable in solution.^30^ In addition to dramatically reducing solvent use, the energy efficiency of mechanochemistry has been demonstrated through dedicated life-cycle assessment (LCA) studies.^29,31^

Over the past few decades, the application of mechanochemistry to amide bond formation has experienced significant progress, evolving from initial proof-of-concept studies to advanced protocols suitable for complex targets. These include peptide synthesis, the production of active pharmaceutical ingredients (APIs), and the first compelling demonstrations of scalable processes. Among the various organic transformations adapted to mechanochemical conditions, amide bond formation stands out as one of the most mature, versatile, and industrially significant areas.

Despite the significant progress achieved in recent years, an up-to-date and comprehensive account of mechanochemical amidation methods is still lacking. Previous focused reviews^32,33^ are now outdated for such a rapidly evolving field, while other available reviews have addressed mechanochemical amide bond formation only within the broader context of organic mechanochemistry or as illustrative examples linked to specific themes such as selected methodologies,^34^ green chemistry metrics,^35^ process-scale considerations,^36^ or application areas.^37^ In contrast, the present article compiles all available literature directly relevant to mechanochemical amidation, summarizing developments from early foundational studies to the most recent advances as of September 2025, and providing a structured and critical evaluation of the field that considers both practical applications and underlying fundamental aspects.

We take a systematic look at the field, beginning in Section 2 with mechanochemical adaptations of classical amidation strategies by reactions of carboxylic acids and amines (Figure 1), including their direct thermal condensation and the widely adopted methods based on preactivation or in situ activation of carboxylic acids with amide coupling reagents. Section 3 shifts focus to less conventional approaches enabled by mechanochemistry, such as the use of amine and carboxylic acid surrogates, redox processes, rearrangements, multicomponent reactions and transition metal-mediated transformations that produce amide products through alternative bond-forming pathways. Section 4 delves into the emerging field of mechanoenzymology, where enzymes are employed under mechanochemical conditions to catalyze amide bond formation. In Section 5, we summarize how stereochemical integrity is maintained during mechanochemical amide synthesis. Finally, Section 6 discusses how amide-forming reactions serve as model systems to elucidate the driving forces of mechanochemistry in mechanistic studies. Throughout the review, we highlight major breakthroughs, showcase representative examples, evaluate the advantages and limitations of various approaches, and consider sustainability metrics and scalability, offering a detailed perspective on this rapidly advancing field.

**Figure 1:**
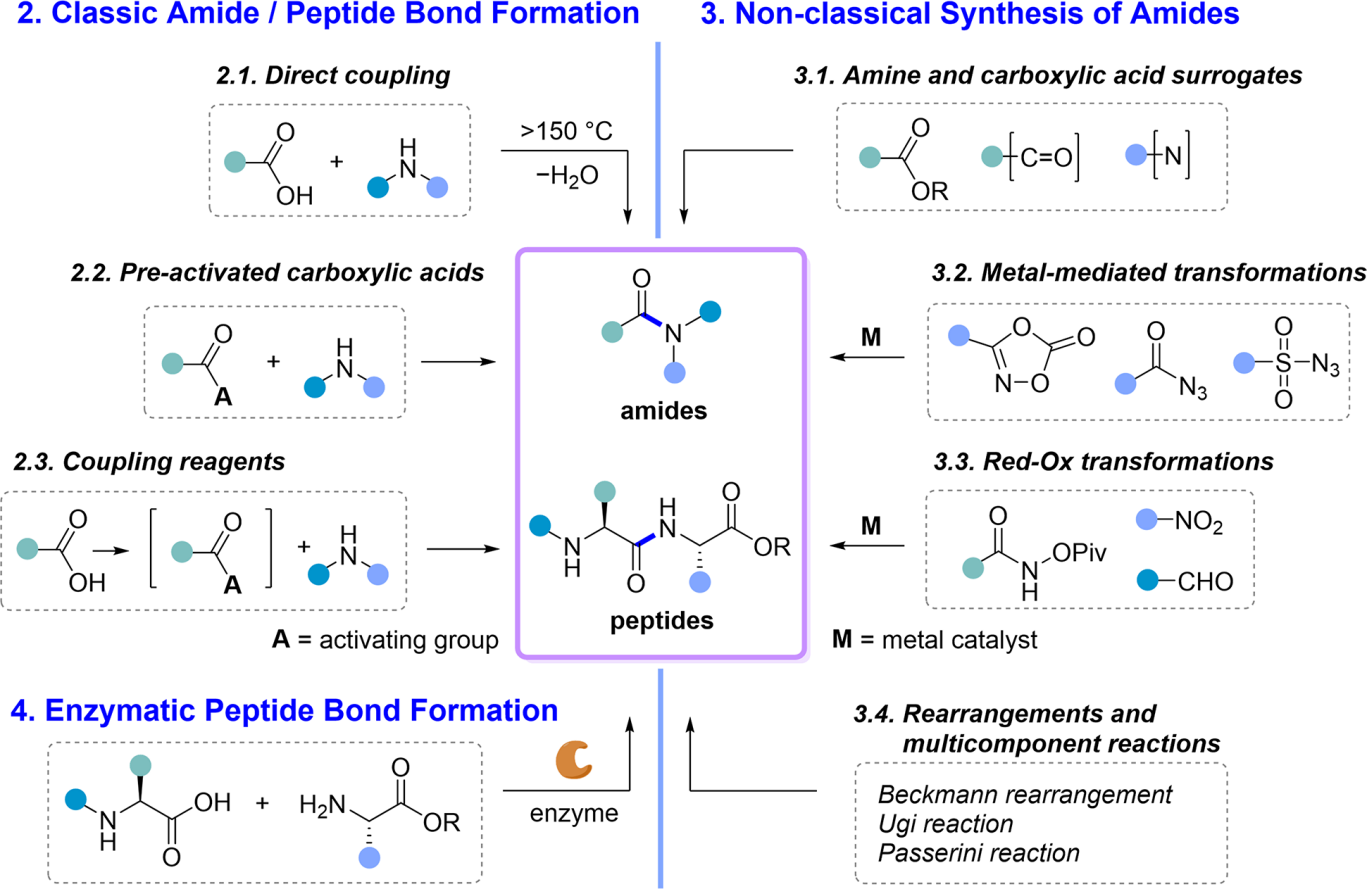
Overview of the chemical methodologies discussed in this review.

A detailed overview of mechanochemical techniques and equipment lies beyond the scope of this review. Specific topics such as instrumentation, scale-up strategies,^36^ and alignment with green chemistry principles^35,38,39^ and sustainability objectives^40^ have been thoroughly addressed in several dedicated publications. Here, we briefly introduce the fundamental concepts of mechanochemistry and describe the primary types of instrumentation, to assist readers who are new to the field.

Mechanochemistry, as defined by IUPAC, refers to chemical reactions induced by the direct absorption of mechanical energy.^41^ This definition covers a broad range of phenomena,^42^ but the most relevant aspect for synthetic chemistry is its capacity to drive chemical transformations in solid-state materials. In practice, this is accomplished using a variety of tools and methodologies (Table 1), ranging from traditional manual grinding with a mortar and pestle to advanced automated systems.

**Table 1:**
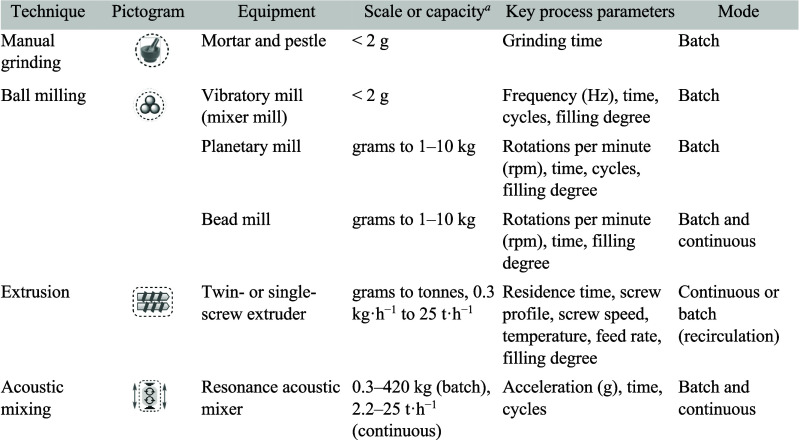
Mechanochemical Techniques and Relevant Technical Details

a Adapted from ref 36.

Ball milling is the most widely employed and established technique. It uses milling media (balls) made of inert materials such as zirconia or stainless steel to grind and mix solid reactants within various types of mills. Vibratory (also called mixer or shaker) mills are the most common in research laboratories. Most reactions discussed in this review were developed using these instruments, which operate by oscillating a pair of milling jars at frequencies up to 30–50 Hz. Planetary ball mills, which support larger vessels, are also widely used. For scale-up applications, high-performance bead milling systems such as the Dyno-Mill offer valuable capabilities.

Twin- and single-screw extruders are another well-established option for scale-up, enabling continuous mechanochemical processing through reactive extrusion. An additional low-impact approach is provided by resonance acoustic mixers (RAM), which gently agitate the reactants by vibrating the reaction vessel at acoustic frequencies, eliminating the need for milling media.

The addition of a small amount of liquid can profoundly influence reactivity, improving mixing and significantly accelerating reaction rates. This approach, known as liquid-assisted grinding (LAG), is quantified by the parameter η (μL·mg^–1^),^300,28^ which represents the volume of liquid additive relative to the mass of solid reactants:

1 $$\eta =\frac{\mathrm{volume of liquid}}{\mathrm{mass of solid reactants}}$$

Finally, the renewed interest in mechanochemistry is closely linked to its strong alignment with the 12 principles of green chemistry. In this review, we go beyond the traditional focus on chemical yield to include additional metrics that offer a more holistic evaluation of reaction efficiency and sustainability. Specifically, we consider key green chemistry indicators such as atom economy (AE), reaction mass efficiency (RME), and process mass intensity (PMI),^43^ which are defined as follows:

2 $$\mathrm{AE}=\frac{\mathrm{molecular weight of product}}{\mathrm{total molecular weight of reactants}}\times 100\%$$

3 $$\mathrm{RME}=\frac{\mathrm{mass of isolated product}}{\mathrm{total mass of reactants}}\times 100\%$$

4 $$\mathrm{PMI}=\frac{\mathrm{total mass in a process}}{\mathrm{mass of product}}$$

It is worth noting that, depending on the materials considered, PMI values may refer either to the reaction PMI (based solely on reagents, reaction solvents and additives) or to the total PMI (which also includes all materials used for workup and downstream processing). Whenever relevant to the discussion, this distinction is specified, and comparable PMI values are used for benchmarking against solution-phase reactions. In many publications, the *E*-factor is employed as an alternative metric.^44^ Since PMI and *E*-factor convey essentially the same information, using both is redundant and can lead to confusion. Therefore, *E*-factors reported in the original studies were recalculated to PMI values using the relationship:

5 PMI=E‐factor+1

In addition to these metrics, space-time yield (STY) is a productivity metric that quantifies the amount of product formed per unit reactor volume and per unit time. It complements green chemistry metrics by normalizing output to the equipment footprint and is useful for benchmarking process intensification across batch and continuous setups. We report STY on a mass basis as:

6 $$STY=\frac{m_{product}}{V_{reactor}\times t}\left[\mathrm{kg}\cdot m^{-3}\cdot h^{-1}\right]$$

where *m*_*product*_ is the isolated product mass, *V*_*reactor*_ is the working reactor volume (mill jar or reacting section of the extruder), and *t* is the processing time (for batch) or the observation window at steady state (for continuous, equivalent to mass-flow/reactor-volume). Higher STY indicates greater throughput from the same hardware.

## 2 Synthesis of Amides by Condensation of Carboxylic Acids and Amines

Condensation of readily available carboxylic acids with primary or secondary amines is the most direct and widely adopted strategy for amide bond formation. This section focuses on how these transformations have been adapted to mechanochemistry, providing the foundation for mechanochemical amide synthesis and reflecting similar processes developed in solution-phase chemistry. These methods commonly include preactivated carboxylic acid derivatives, such as anhydrides, acid chlorides, activated esters, and similar compounds, as discussed in Section 2.2, along with the use of stoichiometric amide coupling reagents, covered in Section 2.3. The final Sections 2.4 and 2.5 discuss the comparison of the methods and provide practical guidelines, respectively. However, we begin with the most straightforward and atom-efficient route: direct thermal condensation of carboxylic acids with amines, presented in Section 2.1. This part serves as a basis for understanding the challenges that necessitate the use of activation strategies addressed later in the chapter.

### 2.1 Direct Amide Coupling of Inactivated Carboxylic Acids

The direct condensation of carboxylic acids and amines is considered the most favorable approach from a green chemistry perspective, as it avoids the use of amide coupling reagents and produces only water as a byproduct.^45–47^ However, it requires high temperatures (typically above 150 °C), likely due to the formation of unreactive ammonium carboxylate salts.^48^ These conditions demand specialized equipment for heating and temperature control, which is not commonly available in standard mechanochemical setups. As a result, investigation of this strategy began only recently.

In 2023, Stolar et al.^49^ reported the first examples (Scheme 1), optimizing the thermally promoted mechanochemical condensation of benzoic acid and *p*-toluidine to produce amide **1**. The reaction was conducted in stainless steel milling jars using a VBM at 30 Hz and heated to 190 °C. A key improvement for achieving high conversions (80–89%) involved opening the hot jar after 1 h of milling to allow the system to cool and the water to evaporate, followed by resuming the milling process. Nearly quantitative conversion was obtained using a 2:1 acid-to-amine ratio. The formation of cocrystal salt intermediates was confirmed via *ex situ* synchrotron PXRD and single-crystal XRD analysis. The method was applied to two examples, demonstrating gram-scale synthesis of amide **1** in 85% yield (isolated by CC and purified by recrystallization) and nearly quantitative preparation of the API moclobemide (**2**), which was isolated pure directly from the milling jar. The direct amidation method achieved high atom economy (92.1% for **1** and 93.7% for **2**) and excellent reaction PMI values of 1.27 and 1.07, respectively, which are close to the ideal value of 1, indicating minimal waste and strong sustainability potential.

**Scheme 1:**
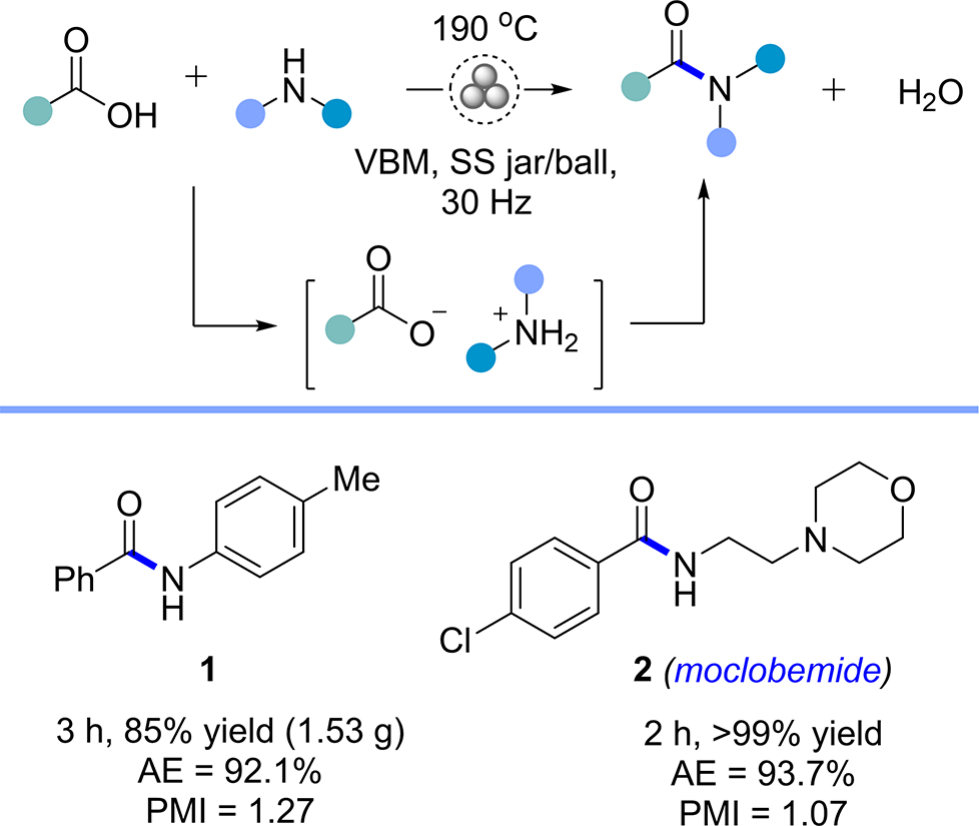
Thermally Promoted Direct Amide Coupling in a VBM

The approach was further advanced by Lavayssiere et al.,^50^ who adapted it to a twin-screw extruder (TSE) setup. Solvent-free synthesis of eight amides was achieved by leveraging the heating and mixing capabilities of a vertical corotating TSE (Scheme 2). Aromatic and aliphatic carboxylic acids, along with anilines and primary aliphatic amines, afforded the corresponding amide products in high yields of 83–97%. Primary and secondary benzylamines gave amides **5** and **6** upon reaction with palmitic acid in high 95% and 97% yields, although the latter required a longer reaction time.

**Scheme 2:**
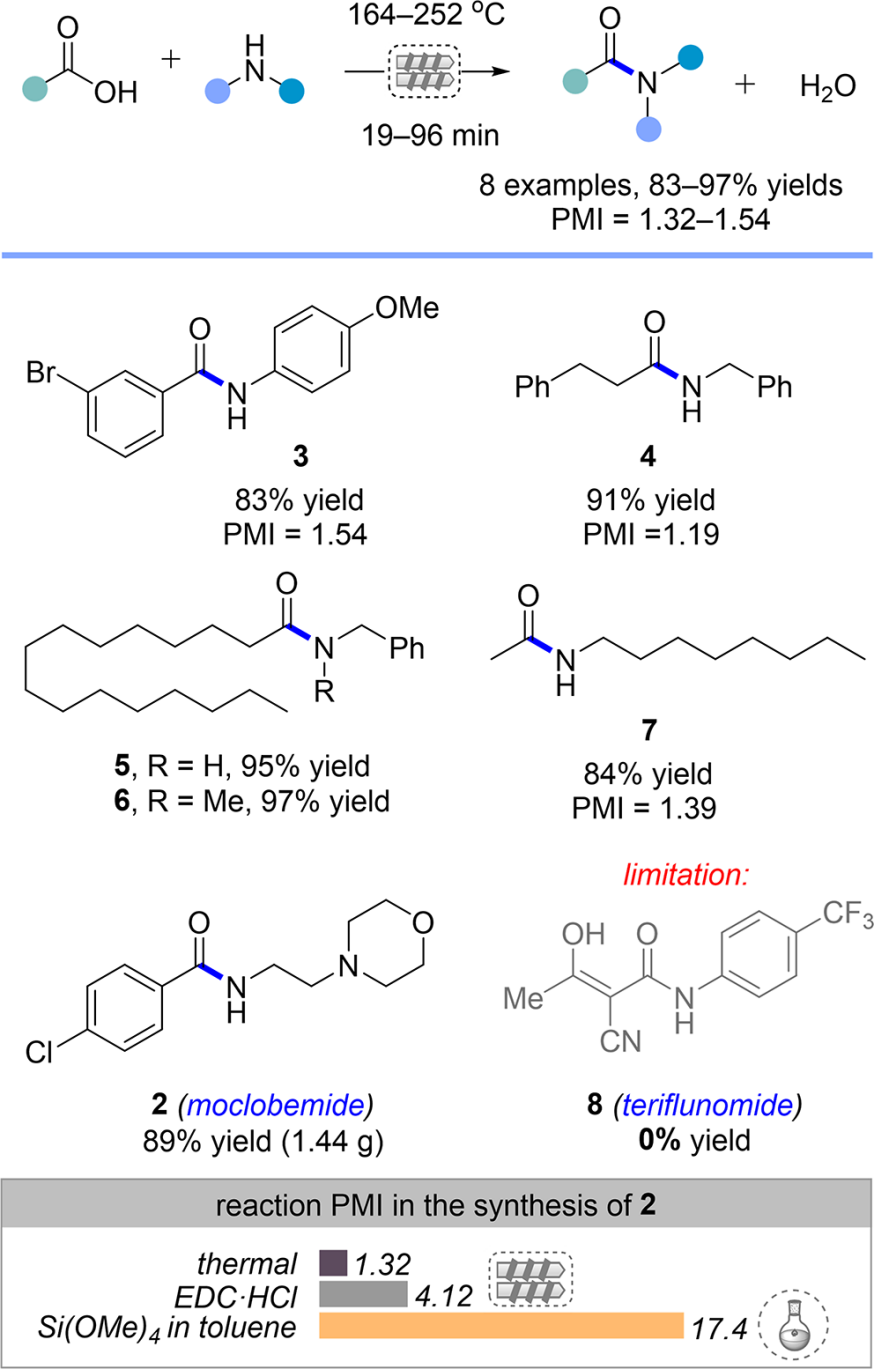
Thermally Promoted Direct Amide Coupling in a Twin-Screw Extruder (TSE), Selected Examples and a Limitation

The preparation of acetylamide **7** required an excess (2.7 equiv) of volatile acetic acid to achieve efficient conversion. A distinctive feature of the methodology was the integration of a Bayesian optimization (BO) algorithm to efficiently explore the multiparameter space (including temperature, reaction time, and acid-to-amine ratio) and identify optimal conditions with a minimal number of experimental runs. For instance, in the synthesis of amide **3**, BO successfully guided the optimization to achieve 92.6% conversion and 83% isolated yield by extruding the neat reactants at 252 °C with a residence time of 19 min.

This approach was also applied to the synthesis of the API moclobemide (**2**). The reaction PMI for compound **2** (PMI = 1.32), obtained in 89% yield, was lower than that of previous mechanochemical syntheses via extrusion using an amide coupling reagent (PMI = 4.12),^51^ and approximately 13-fold lower compared to direct amidation mediated by tetramethyl orthosilicate in toluene solution (PMI = 17.4).^52^ Nevertheless, it is important to note that solvents such as ethanol and water, as well as reagents like aqueous KOH, were still required during the workup step in the extrusion approach. The pure products were obtained either by extraction or by recrystallization. Consequently, the total PMI exceeds the reaction PMI, suggesting that further optimization of the isolation protocol may be required. Currently, a fair comparison of total PMIs for the mechanochemical and the solvent-based protocols (total PMI = 141)^53^ cannot be done.

In contrast to amide **2**, synthesis of the API teriflunomide (**8**) was unsuccessful, resulting in the formation of tar in the extruder, likely due to thermal decomposition of 5-methylisoxazole-4-carboxylic acid precursor.

The study also highlighted that the evaporation of water at elevated temperatures contributed to shift the equilibrium toward amide formation. Overall, this work demonstrates the effective combination of continuous mechanochemical processing and BO algorithm in developing sustainable and efficient amidation protocols.

Despite the nearly ideal reaction PMIs and high AE of thermally promoted direct amide coupling, the use of elevated temperatures introduces several restrictions to substrate scope due to possible thermal degradation of sensitive compounds (e.g., the unsuccessful synthesis of **8**), promotion of side reactions such as diketopiperazine formation from amino acids and peptides,^54^ and incompatibility with substrates of low boiling points. These limitations dictate the selection of suitable substrates, which must tolerate temperatures of at least 150–200 °C and be free of thermolabile functionalities. Furthermore, the extent to which the reaction equilibrium is influenced by water volatility may be case-dependent. Additionally, high-temperature operation raises concerns regarding thermal safety and energy efficiency from a green chemistry perspective.

To address these issues, reactions should be conducted at lower temperatures, well below the decomposition and boiling points of thermally unstable or volatile substrates, preferably under ambient conditions. Achieving such reactivity typically requires the use of activated carboxylic acid derivatives, in which the hydroxyl group is replaced by a more effective leaving group. These strategies, adapted for mechanochemical applications, currently represent the mainstream approach in amide and peptide coupling and are presented below.

### 2.2 Amide Coupling of Preactivated Carboxylic Acids

To enable amide bond formation under ambient temperatures, prior activation of the carboxylic acid is necessary to replace the hydroxyl group with a better leaving group.^55^ This is also the case in living systems, where peptide bonds are formed on the ribosome through template-directed coupling of amino acids delivered as tRNA ester derivatives.^56^ The activation strategy predominates in the established mechanochemical methods for amide bond formation (Scheme 3).

**Scheme 3:**
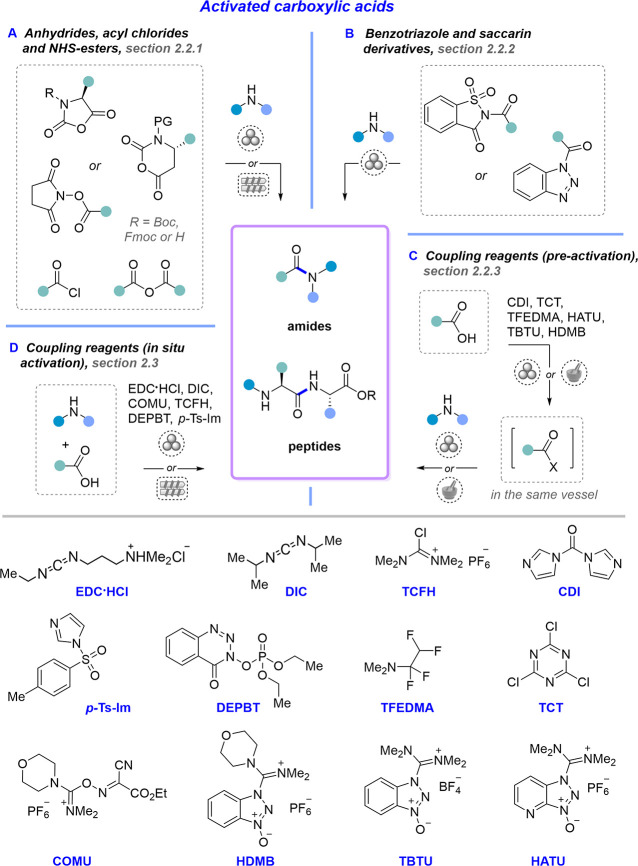
Overview of Strategies Involving Preactivation of Carboxylic Acids and Structures of Amide Coupling Reagents

The activation of carboxylic acids followed by their reaction with amines can be carried out using three main approaches. First, the acylating reagent can be synthesized and isolated in advance through conventional methods, unless it is available from commercial sources. Examples include anhydrides, acyl chlorides, NHS esters, benzotriazole and saccharin derivatives. These intermediates are then reacted with amines in a separate step (Scheme 3, A and B; see Sections 2.2.1 and 2.2.2). Second, an activated derivative such as an acylimidazole, activated ester, or acyl fluoride can be generated directly from the carboxylic acid using mechanochemistry. The reaction with the amine then proceeds in the same milling vessel in a stepwise manner without isolating the intermediate (Scheme 3, C, discussed in Section 2.2.3). Finally, the acylating agent can be formed *in situ* from the carboxylic acid in the presence of the amine, using various coupling reagents and allowing for single-step reactions. This approach is especially effective for amide and peptide synthesis,^3,57^ and better suitable for scale-up (Scheme 3, D, covered in Section 2.3).

#### 2.2.1 Anhydrides, Acyl Chlorides and NHS-Esters

Reactions of amines with anhydrides are among the earliest examples of mechanochemical amide synthesis, applied to API synthesis^301^ and nucleoside protection.^302,303^ For example, in 2008, Giri et al.^58^ reported mechanochemical *N*-benzoylation with benzoic anhydride in a VBM to protect the amino group in cytidine **9**, achieving high 90% of the product **10** (Scheme 4).

**Scheme 4:**
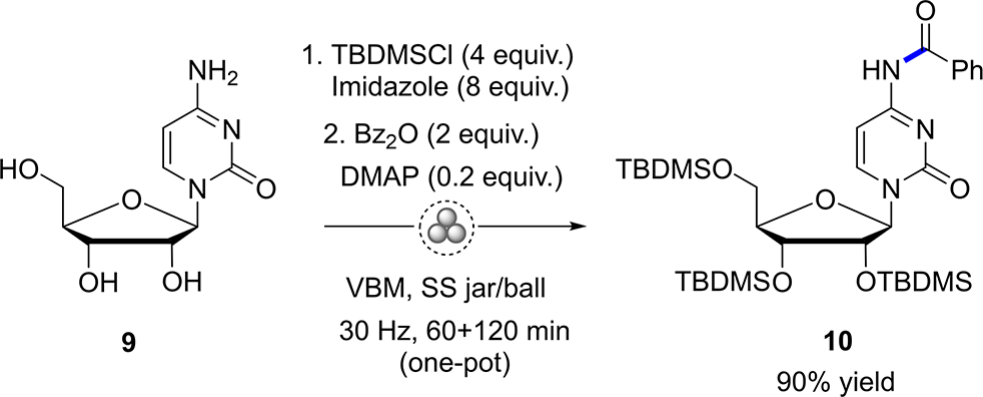
Mechanochemical N-Benzoylation of Cytidine

In 2009, the first example of peptide bond formation under solvent-free conditions was reported by Declerck et al.^59^ A range of dipeptides was obtained by ball milling of urethane-protected α-amino acid *N*-carboxyanhydrides (UNCA) with α-amino ester hydrochlorides and NaHCO_3_ in a steel milling vessel with steel balls at 30 Hz (Scheme 5). Notably, Boc-protected valine and phenylalanine NCAs were coupled almost quantitatively (97–100% conversion) with various amino acid counterparts (Leu, Ala, Phe, Gly), whereas Fmoc-protected valine NCA reacted with reduced 78–93% conversions.

**Scheme 5:**
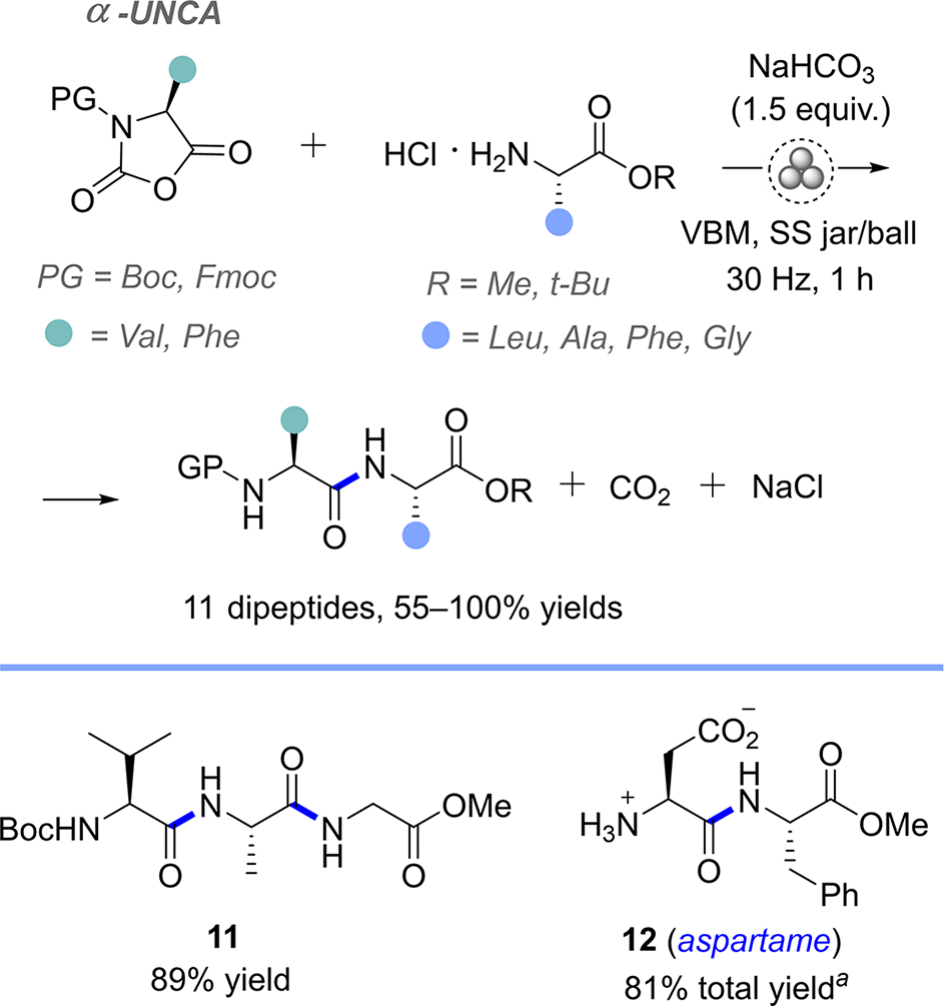
Synthesis of Di- and Tripeptides by Using UNCA Derivatives

a Yield after Boc-deprotection with HCl­(g) and adjusting pH to the isoelectric point (pH 5.0) with aq. Na_2_CO_3_.

All the dipeptides were isolated by extractive workup in 55–100% yields. No epimerization of stereocenters in amino acids was detected by HPLC analysis. In addition, the reaction proceeded with the same efficiency at lower milling frequencies, albeit required longer time (5 h at 10 Hz vs 2.5 h at 20 Hz). Using this methodology, tripeptide **11** was synthesized in 89% yield, while the sweetener aspartame (**12**) was obtained in 81% overall yield over three steps. This sequence included the removal of Boc and *t*-Bu ester protecting groups using gaseous HCl in a gas–solid reaction, carried out without any solvent.

However, the developed approach had a limitation of relatively low milling load (5.9 mg·mL^–1^), referring to the amount of reactant material relative to the internal volume of the milling jar. In 2013, the same group^60^ reported a methodology for the scaled-up synthesis of small peptide synthesis, as well as longer peptide chains containing up to five amino acid residues (Scheme 6).

**Scheme 6:**
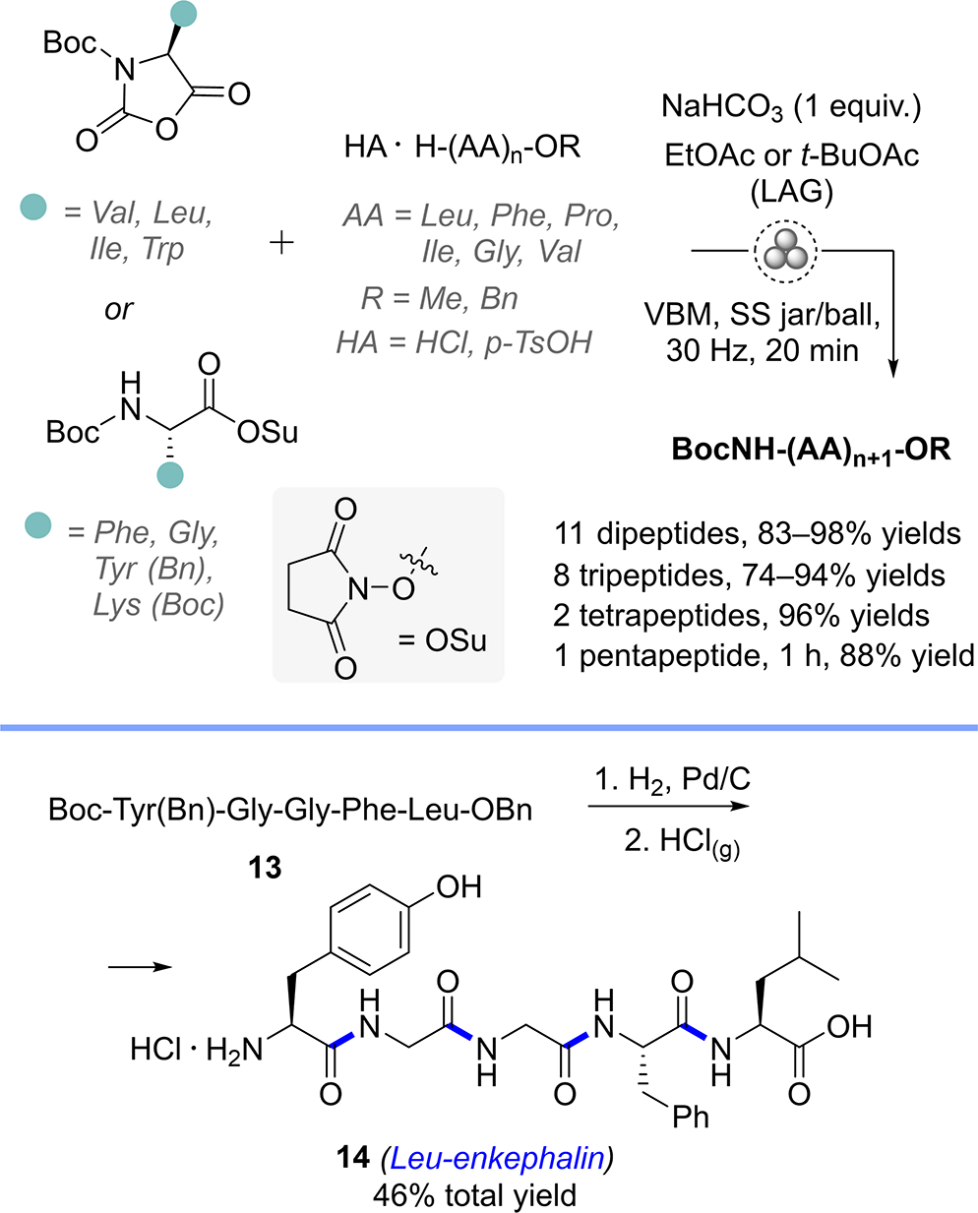
Synthesis of Di-, Tri-, Tetra- and Pentapeptides by Using UNCA and NHS Derivatives

Increasing the milling load to 22.5 mg·mL^–1^ led to a sharp decrease in reaction rate, attributed to the formation of a highly viscous and sticky reaction mixture that hindered effective mixing, along with partial hydrolysis of the UNCA derivative. To overcome this issue, ethyl acetate was introduced as a LAG additive (η = 1.4 μL·mg^–1^). Notably, conventional stirring with ethyl acetate or DMF at the same η value resulted in dramatic drop in conversion, highlighting the essential role of ball milling.

The optimized conditions were then applied to the synthesis of a diverse set of dipeptides, which were isolated as pure compounds after extraction workup in 83–98% yields. The scope of the methodology was further expanded by replacing UNCA derivatives with commercially available Boc-protected α-amino acid *N*-hydroxysuccinimide (NHS) esters. HPLC analysis confirmed that no significant epimerization occurred under the reaction conditions. A green chemistry assessment was performed using the Ecoscale scoring system.^61^ For the synthesis of the Boc-Tyr­(Bn)-Leu-OMe dipeptide, the method achieved an Ecoscale score of 84 out of a maximum of 100, outperforming a comparable solution-based protocol (score of 69) that employed triethylamine as a base and DMF as the solvent.

The synthesized Boc-dipeptides were subsequently deprotected using gaseous HCl, and the resulting dipeptide hydrochlorides were coupled with various UNCA or NHS derivatives to furnish tripeptides in 74–94% yields (Scheme 6). Notably, the reaction was compatible with amino acids bearing heteroatoms in their side chains, such as lysine, as well as sterically demanding residues like leucine and isoleucine. The synthesis of Boc-Gly-Phe-Leu-OBn tripeptide was successfully scaled up to a milling load of 152.7 mg·mL^–1^, affording a 94% yield (937 mg). Two tetrapeptides Boc-(Leu)_4_-OBn and Boc-Gly-Gly-Phe-Leu-OBn were synthesized in 96% yields. Finally, pentapeptide **13**, a precursor of Leu-enkephalin (**14**), was obtained in 88% yield by coupling of tetrapeptide HCl·H-Gly-Gly-Phe-Leu-OBn with Boc-Tyr­(Bn)-OSu. The total synthesis of Leu-enkephalin was completed by catalytic hydrogenolysis of the benzyl protecting groups, followed by Boc group removal using gaseous HCl. After nine alternating coupling and deprotection steps, Leu-enkephalin (**14**) was obtained in an overall yield of 46%.

The reactive extrusion approach was harnessed for further scale-up of the methodology (Scheme 7).^62^ Following brief optimization, acetone was identified as the most effective LAG additive (η = 0.15 mL·g^–1^) to improve the flow of the reaction mixture within the extruder chamber. Recirculating the mixture for 10 min at 40 °C and 150 rpm resulted in complete conversion to dipeptide **15**, which was isolated in 85% yield (3.72 g) after extractive workup, with complete retention of the enantiomeric purity (>99% *ee*).

**Scheme 7:**
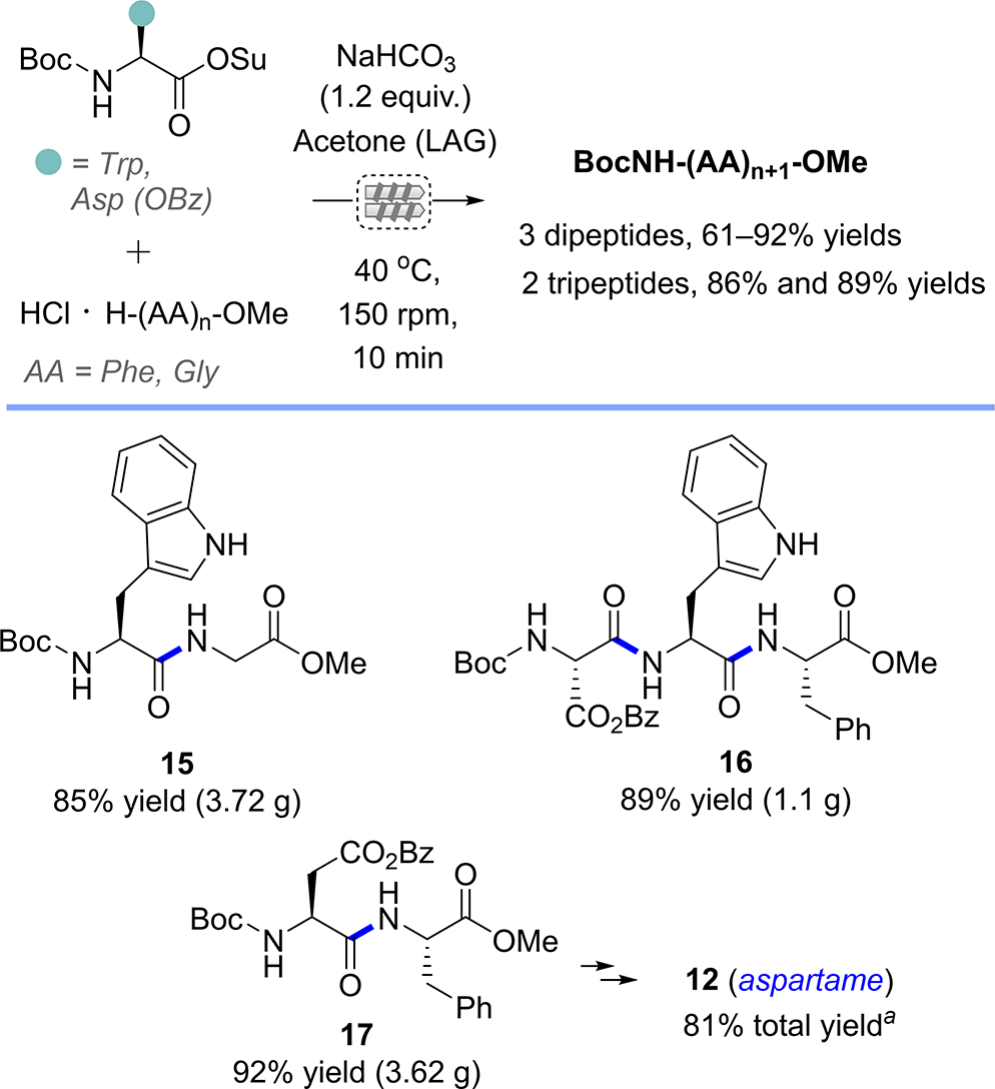
Synthesis of Di- and Tripeptides via Reactive Extrusion

a Yield after Boc-deprotection with HCl­(g) and adjusting pH to the isoelectric point (pH 5.0) with aq. Na_2_CO_3_.

The optimized reaction conditions were then applied to the synthesis of several di- and tripeptides. Following Boc removal using gaseous HCl, the resulting dipeptides were further converted into tripeptides, including tripeptide **16**, obtained in 89% yield (1.1 g). An upscaled synthesis of aspartame (**12**) was also demonstrated, achieving a total yield of 81%. Notably, the dipeptide precursor **17** was obtained in 92% yield (3.62 g) and excellent diastereoselectivity (>99% *de*) with a residence time of only 1.5 min in the extrusion barrel.

Space-time yields (STY) were calculated for the synthesis of several dipeptides using reactive extrusion, ball milling, and solution-phase methods (with triethylamine and DMF). STY is defined as the amount of final product produced per unit reactor volume per unit reaction time, expressed in g·cm^–3^·day^–1^. For example, in the synthesis of dipeptide **17**, the STY of the reactive extrusion method was significantly higher than that of ball milling and solution-based approaches (471.2 vs 2.2 and 4.9 g·cm^–3^·day^–1^, respectively).

Hernández and Juaristi^63^ explored the extension of the UNCA-strategy toward the preparation of α,β- and β,β-dipeptides by coupling of β-amino acid UNCAs with hydrochloride salts of α- and β-amino esters (Scheme 8).

**Scheme 8:**
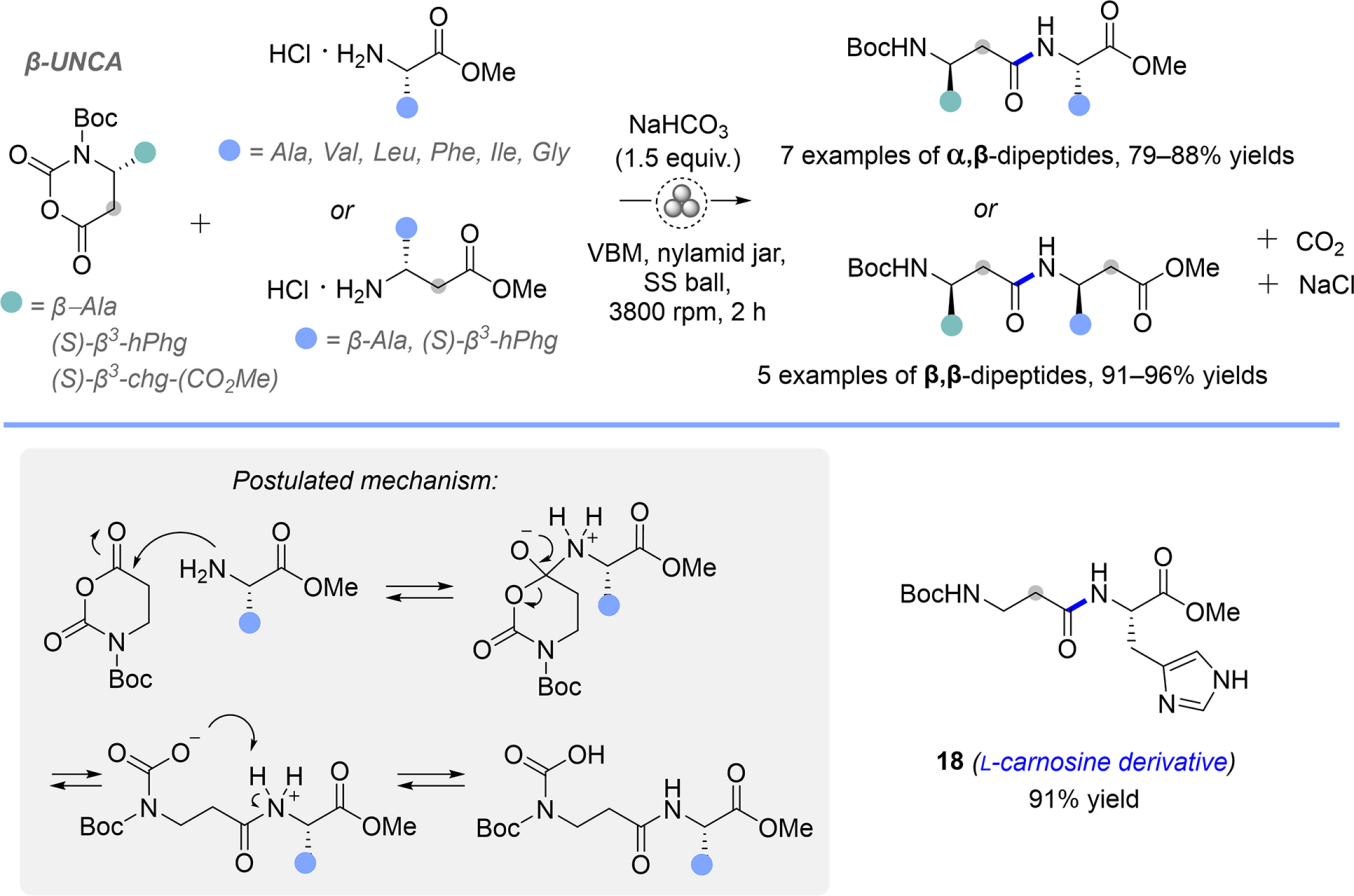
Synthesis of α,β- and β,β-Dipeptides via the UNCA-Strategy^a^

a (*S*)-β^3^-hPhg = (*S*)-β^3^-homophenylglycine, (*S*)-β^3^-chg-(CO_2_Me) = (*S*)-β^3^-carboxyhomoglycine.

Notably, the reactivity of β-UNCAs is generally lower compared to their α-analogues. Using high-energy ball milling, several α,β- and β,β-dipeptides were synthesized in 79–96% yields. Dipeptide **18**, a derivative of the natural compound L-carnosine, was obtained in 91% yield. The absence of epimerization was confirmed by comparison of specific optical rotations. A reaction mechanism was proposed by analogy with the ring-opening polymerization of α-amino acid NCAs.

In 2021, Santino et al.^64^ demonstrated the use of unprotected NCAs for peptide synthesis (Scheme 9), eliminating the need for *N*-protection and deprotection steps but introducing the risk of NCA self-polymerization. To mitigate this, coupling reactions were performed in the presence of hydroxyapatite (Ca_10_(PO_4_)_6_(OH)_2_, HAp), a weak base, and γ-valerolactone (GVL) as a green LAG additive. After 30 min of milling, the reaction mixture was diluted with ethanol, HAp was separated, and the solution was acidified.

**Scheme 9:**
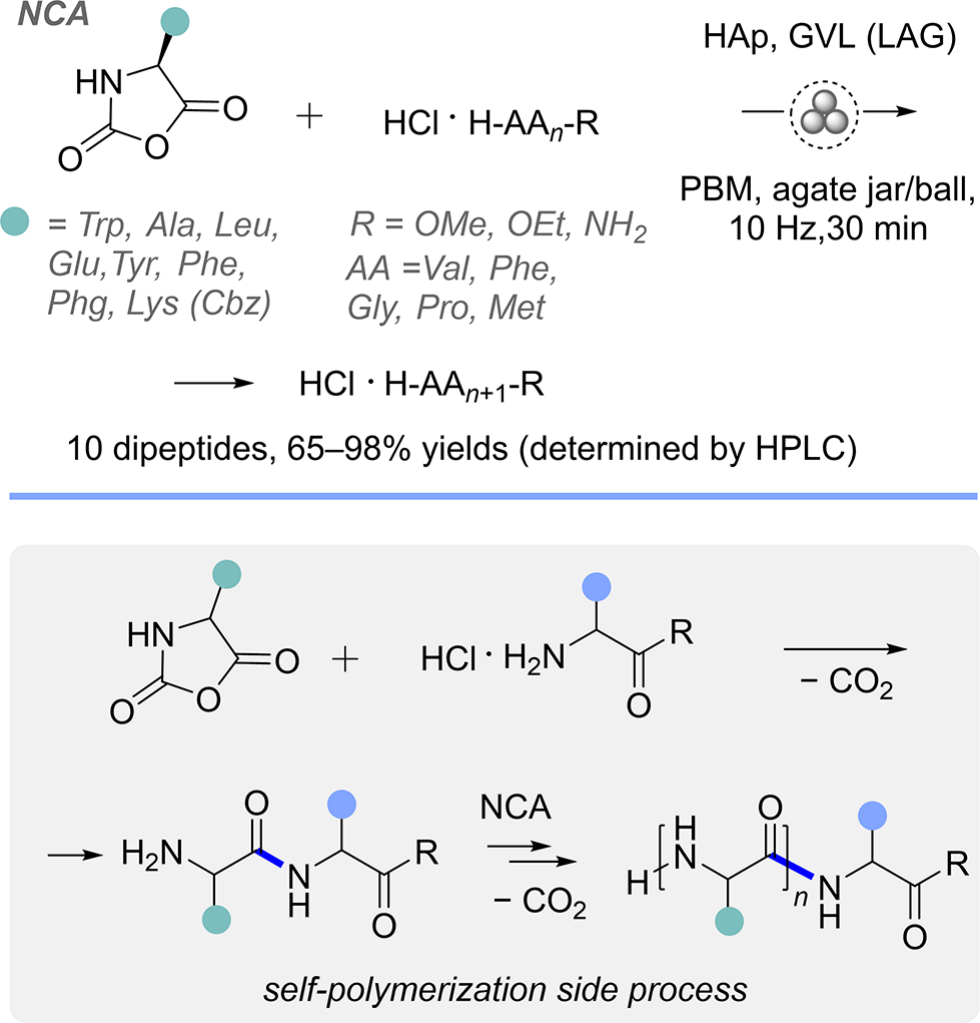
Synthesis of Dipeptides by Using Unprotected NCAs

The resulting dipeptide hydrochlorides were acetylated with excess Ac_2_O for HPLC analysis. HAp could be reused for at least four additional cycles without loss of yield. The study highlighted the importance of NCA purity and crystallinity, confirmed by NMR, SEM, and XRD analyses. For example, HCl·H-Val-OMe coupled with amorphous tryptophan NCA (TrpNCA), showing disordered aggregates in SEM images, yielded 30% polymerization byproducts, including tri- and tetrapeptides. In contrast, recrystallized TrpNCA, with well-defined crystals, reduced these side products to 18%. The method was applied to several dipeptides, achieving HPLC-determined yields of 65–98%, although isolated yields were not reported. Peptide bonds formed chemoselectively with NCAs of glutamine and tyrosine bearing unprotected side chains, and reactions proceeded without significant epimerization, as verified by HPLC.

In 2011, Ravalico et al.^65^ reported a method for the mechanochemical base-mediated acylation of amines using NHS esters. The reaction was completed within 10 min, providing yields equal to or higher than those obtained with conventional DMF-based protocols (Scheme 10).

**Scheme 10:**
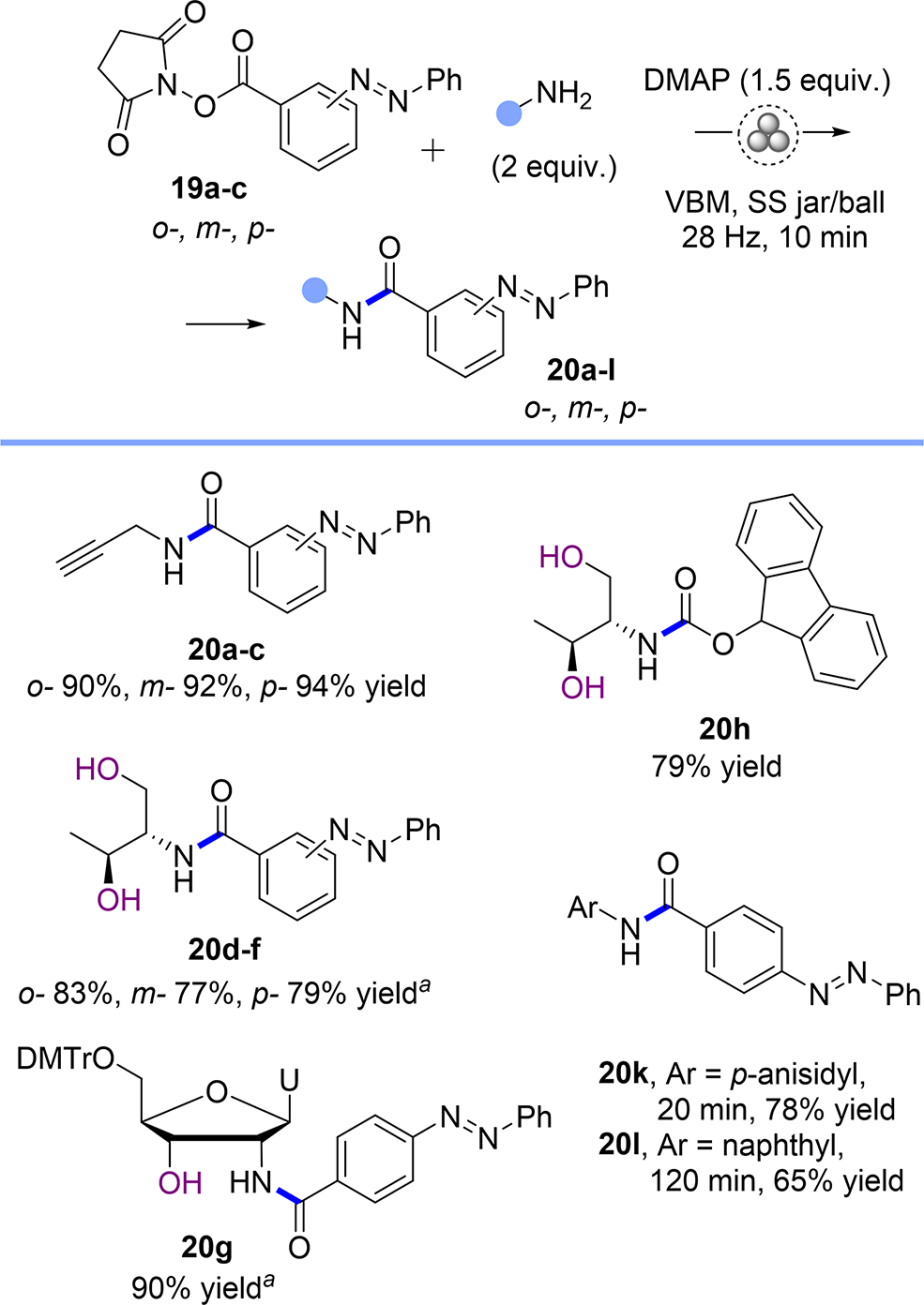
Synthesis of Azobenzene-Derivatized Amides

a Ethyl acetate was used as LAG additive. U = *N*-1-uracilyl.

Key factors influencing the yield of amide **20a** included milling frequency (optimal at 28 Hz), the equivalents of amine and base, and the choice of base. Among the bases tested, DMAP outperformed DABCO, DBU, and Na_2_CO_3_. It was found that under mechanochemical conditions, the reaction rate is influenced not only by the basicity of the base but also by its physical properties, such as melting point. The faster rate observed with DMAP may result from more rapid liquefaction of the reaction mixture during milling. This behavior was observed as the mixtures formed pastes in the early stages of milling and became powders upon completion. A distinct advantage of the ball milling approach was its suitability for reactions involving photosensitive groups. This made the method particularly effective for incorporating azobenzene chromophores via the acylation with esters **19**. The corresponding amides **20** (10 examples) were obtained in 65–94% yields. The use of LAG (ethyl acetate) was necessary to improve the yields of d-threoninol and deoxynucleoside derivatives **20d**-**h**, which could not be efficiently isolated from DMF-based reactions. Notably, the acylation occurred chemoselectively at the nitrogen atom, leaving the hydroxyl groups unaffected.

In 2025, Daurio et al.^66^ demonstrated a reactive extrusion approach for the synthesis of di- and tripeptides using UNCA or NHS derivatives of amino acids and sodium bicarbonate as a base. The protocol was successfully scaled up to a throughput of 1 kg·h^–1^ and demonstrated compatibility with common protecting and leaving groups, as well as a wide range of commercially available amino acids.

Urethane protecting groups at the *N*-terminus of amino acids are essential in peptide synthesis.^304^ In 2013, Konnert et al.^67^ reported the preparation of Boc-, Cbz-, and Fmoc-protected α-amino acids using a planetary ball mill (Scheme 11).

**Scheme 11:**
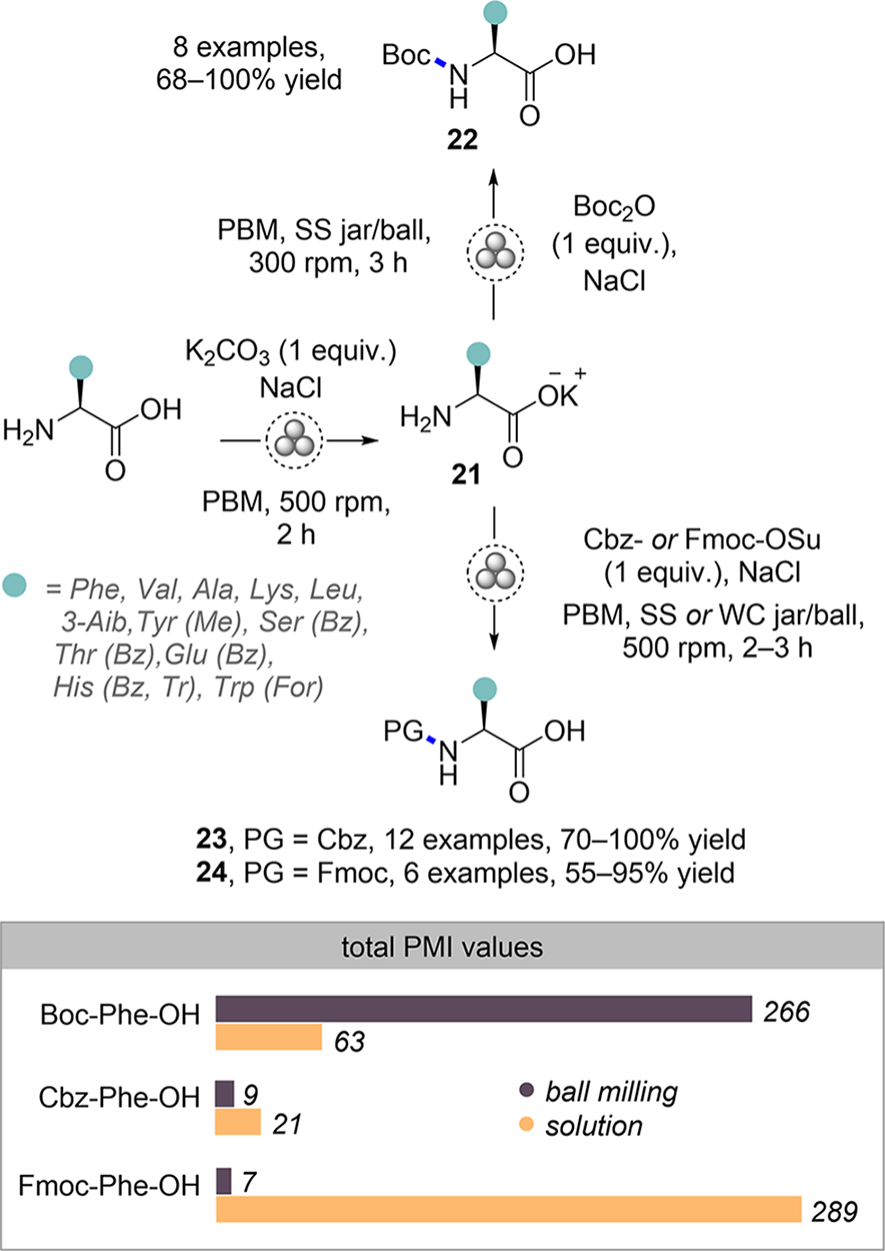
*N*-Protection of AAs with Urethane PGs

The protocol involved the initial generation of a potassium salt **21** via reaction with K_2_CO_3_, followed by treatment with Boc_2_O, CbzOSu or FmocOSu. The addition of NaCl as a grinding auxiliary helped maintain the reaction mixture in a powder form. This method enabled the synthesis of various Boc-, Cbz-, and Fmoc-protected α-amino acids **22**–**24** in 55–100% yields, on scales ranging from 50 mg to 1 g. For the synthesis of Cbz- and Fmoc-protected phenylalanine, total PMI values of 9 and 7, respectively, were achieved, which are 2.3- and 41-fold lower than those of the corresponding solvent-based methods. This improvement arises from the absence of bulk solvent and the simplicity of the isolation procedure in the mechanochemical protocols, which involved only the addition of 1 M HCl, filtration, and washing with water. In contrast, isolation of the Boc-protected product required solvent–solvent extraction, resulting in a significantly higher total PMI of 266.

In 2020, Shi et al.^68^ reported a DMAP-catalyzed *N*-*tert*-butoxycarbonylation of amides using a planetary ball mill (Scheme 12). The reaction of primary amides with Boc_2_O led exclusively to the formation of di-Boc-amides **25** in 81–97% yields, with no mono-Boc intermediates detected. In contrast, secondary amides reacted more slowly, affording *N*-Boc-amides **26** in 35–99% yields. A series of intermolecular competition experiments revealed excellent selectivity, not only between primary and secondary amides but also among secondary amides bearing electronically distinct substituents on the NH group. In particular, the presence of electron-withdrawing groups on the NH moiety significantly enhanced the reaction rate.

**Scheme 12:**
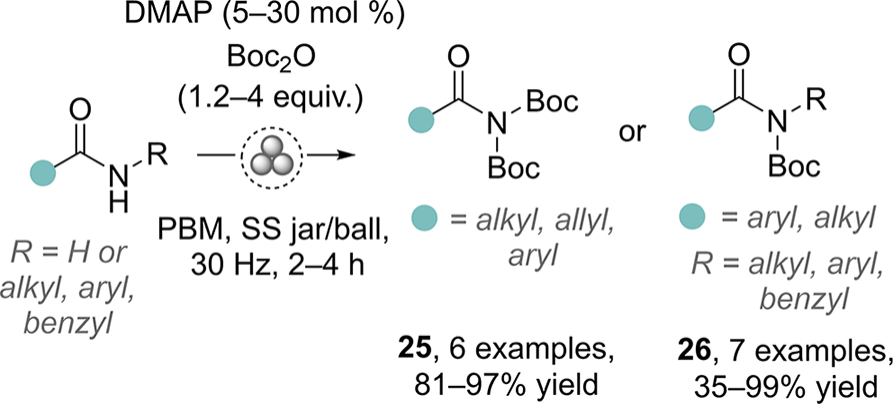
Boc-Protection of Amides

Beyond carbamate protection, other carbonic acid amide derivatives can be efficiently synthesized by high-speed ball milling, including hydantoins from amino acid esters and potassium cyanate,^305^ and oxazolidinones from aziridines and CO_2_.^306^

In 2018, Portada et al.^69^ reported the acylation of amines using *p*-nitrobenzoyl chloride and acetic anhydride, as the key steps of gram-scale mechanochemical syntheses of the antiarrhythmic drug procainamide **27** and analgesic paracetamol **28** (Scheme 13). For procainamide, K_2_CO_3_ was used as a base and silica as a milling auxiliary, followed by reduction of the nitro group under ball milling conditions. Similarly, Park et al.^307^ synthesized paracetamol by one-pot hydrogenation of 4-nitrophenol followed by acetylation with acetic anhydride in a ball mill.

**Scheme 13:**
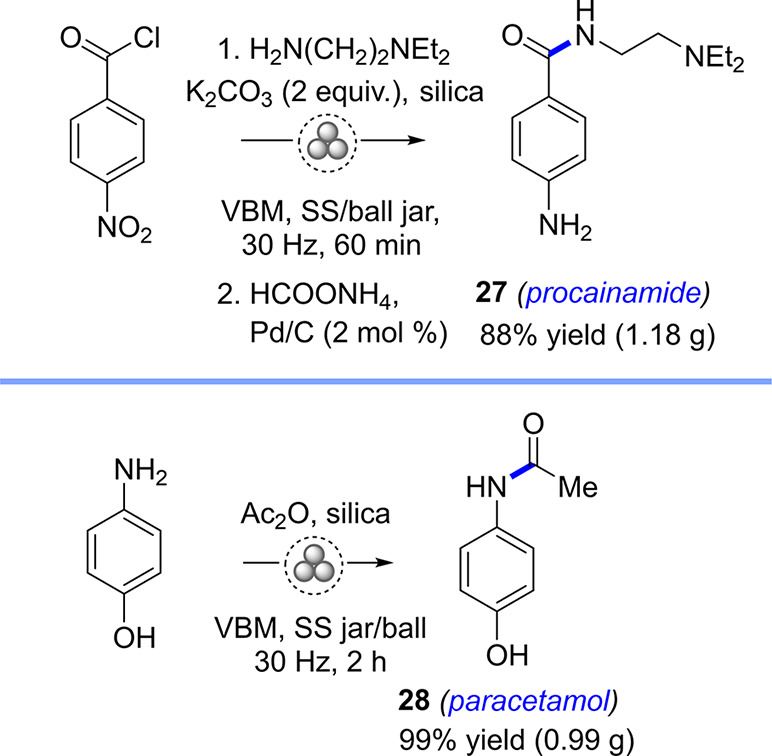
Synthesis of Procainamide and Paracetamol

Mechanochemical acylation with acid chlorides has proven effective for the synthesis of polyamides (Scheme 14). For example, Rajput and Banerjee^70^ synthesized two porous organic polymers **29a**–**b** by amide coupling of 1,3,5-benzenetricarbonyl (trimesoyl) chloride **30** with *p*-phenylenediamine or benzidine in the presence of triethylamine using manual grinding. Although the mechanochemically synthesized polymers exhibited a smaller surface area and moderate adsorption properties compared to those synthesized in solution, they demonstrated exceptional stability in water and concentrated acids. Similarly, Yang et al.^71^ reported a straightforward mechanochemical synthesis of 2D aromatic polyamides. Their study highlighted that solvent-free conditions were crucial for achieving highly crystalline 2D aromatic polyamides, in contrast to the amorphous products obtained through conventional solution-based methods.

**Scheme 14:**
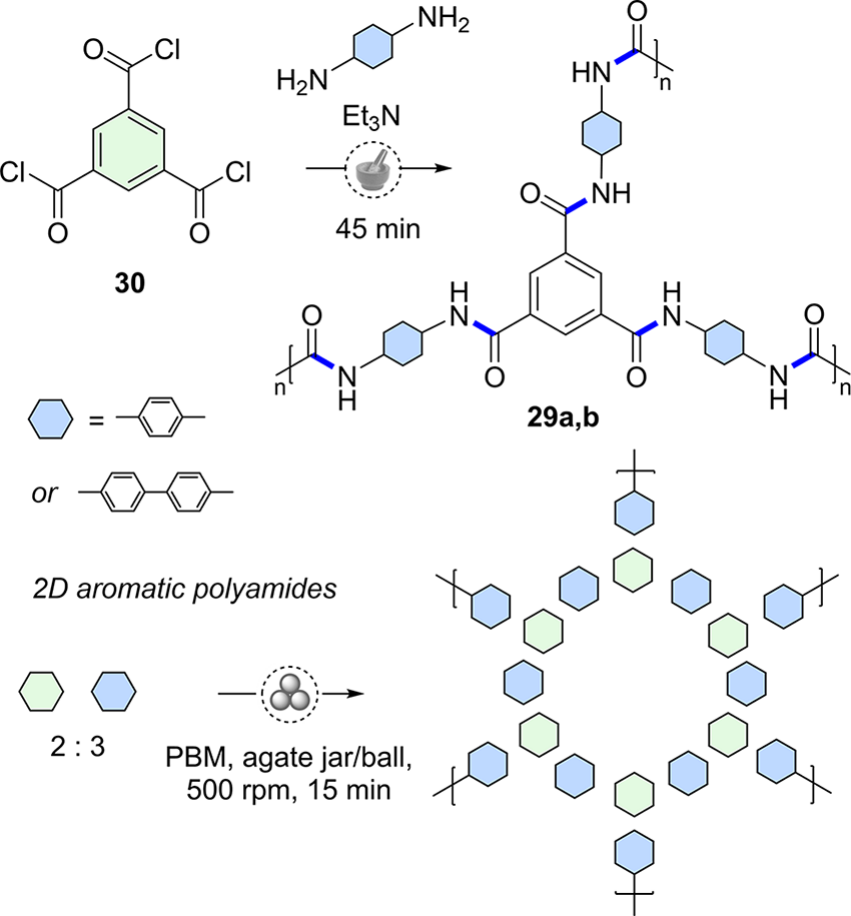
Synthesis of 2D Polyamides from Trimesoyl Chloride

An early example of imide synthesis under mechanochemical conditions was reported by Kaupp et al.,^72^ who prepared phthalimide **31** by ball milling of phthalic anhydride with 4-toluidine in almost quantitative 99% yield (Scheme 15, A). Subsequently,^73^ a similar mechanochemical synthesis of the fluorimetric probe tetraphenylethylene phthalimide **32** was demonstrated, obtained in a high 94% yield. This probe was effectively used for the selective and sensitive detection of hydrazine in solid, liquid, and vapor phases.

**Scheme 15:**
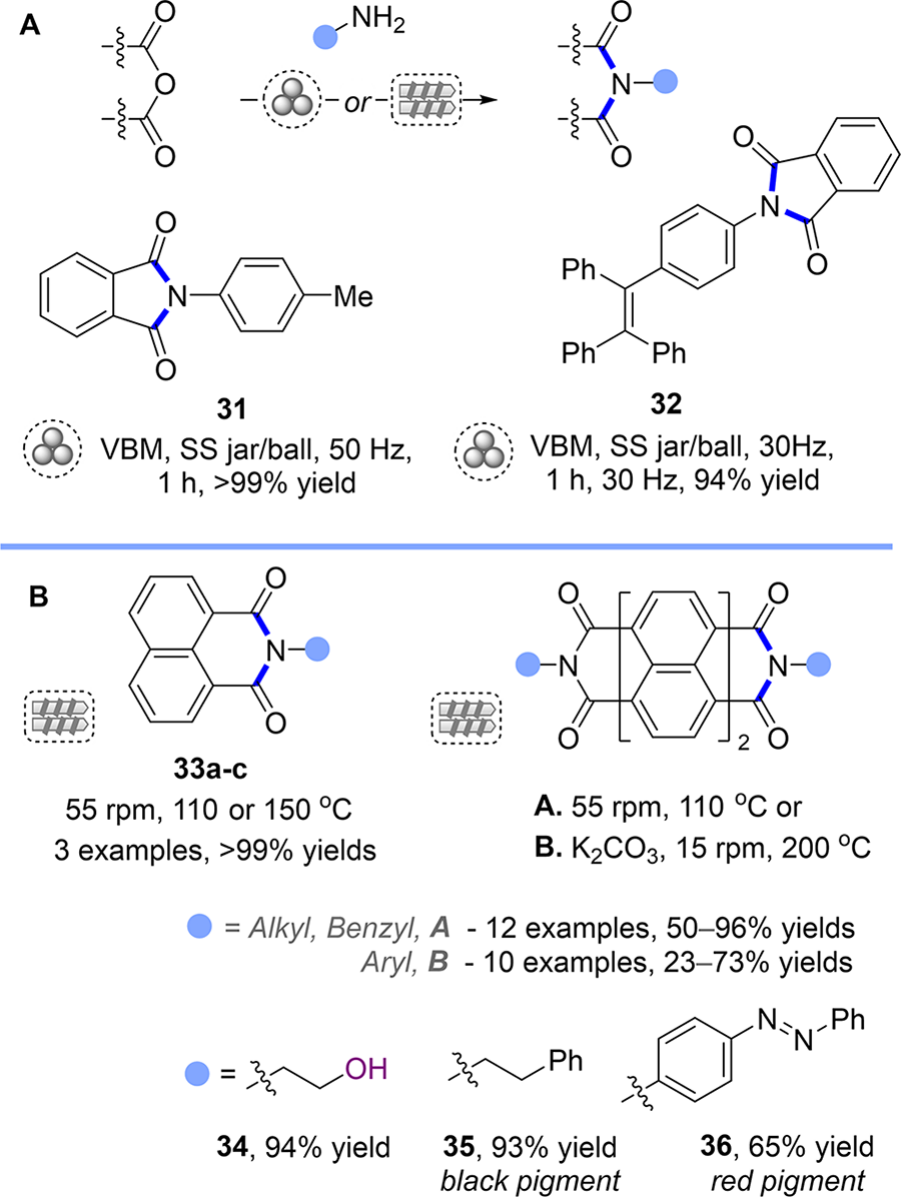
Synthesis of Imides and Diimides from Anhydrides

In 2020, a continuous, scalable, and solvent-free method for the synthesis of various naphthalic imides and perylene diimides (PDIs) by TSE was reported by Cao et al.^74^ (Scheme 15, B).

For instance, three naphthalic imides **33a**–**c** were produced quantitatively without the need for product purification. In the more challenging synthesis of PDIs, alkyl and benzyl amine derived PDIs were obtained in 50–99% yields with good functional-group tolerance, including free hydroxy group containing PDI **34** (94% yield). The electronic properties of benzylamine substrates had a notable impact on the yield, with electron-donating groups leading to higher conversions. The use of K_2_CO_3_ enabled the synthesis of otherwise challenging aniline-derived PDIs in 23–73% yields. The reaction was effective for anilines bearing a range of ring substituents (-OMe, -OEt, -NO_2_, -Br, and phenyldiazenyl). However, sterically hindered anilines were less compatible. As a demonstration of practical applicability, five commercial red and black PDI pigments were synthesized, including black pigment 31 (**35**) and red pigment 178 (**36**). Moreover, an automated continuous TSE process for manufacturing PDIs was successfully demonstrated, producing two black pigments (including **35**) in quantitative yields on a kilogram per day scale.

#### 2.2.2 *N*-Acyl Benzotriazole and Saccharin Derivatives

In addition to anhydrides and NHS esters, *N*-acyl benzotriazoles have emerged as valuable preactivated intermediates for mechanochemical peptide synthesis, providing an alternative route to amide bond formation under solvent-free or LAG conditions. *N*-Acyl benzotriazoles represent robust, isolable acyl donors for mechanochemical amidation, combining high intrinsic reactivity with superior shelf stability and reduced hydrolytic lability relative to NHS esters. Their efficient coupling performance obviates the need for additional activating agents.

The application of *N*-acyl benzotriazoles to the synthesis of peptides was initiated by the Colacino group (Scheme 16).^75^ A number of dipeptides was obtained in variable 20–98% yields by ball milling of *N*-protected-α-amino acyl benzotriazole derivatives **37** with several α- or β-amino acid hydrochlorides in the presence of DIEA and, in some cases, ethyl acetate as LAG additive. The outcome of the reaction was influenced by the nature of both benzotriazole derivatives and amine partners, as well as by the nature of the protecting group used. Notably, partial Fmoc deprotection was observed during milling under LAG conditions, while it was not detected under neat grinding conditions. This contrast to the reactions of NHS esters, that tolerate LAG conditions but undergo faster hydrolysis, limiting reproducibility in prolonged milling.

**Scheme 16:**
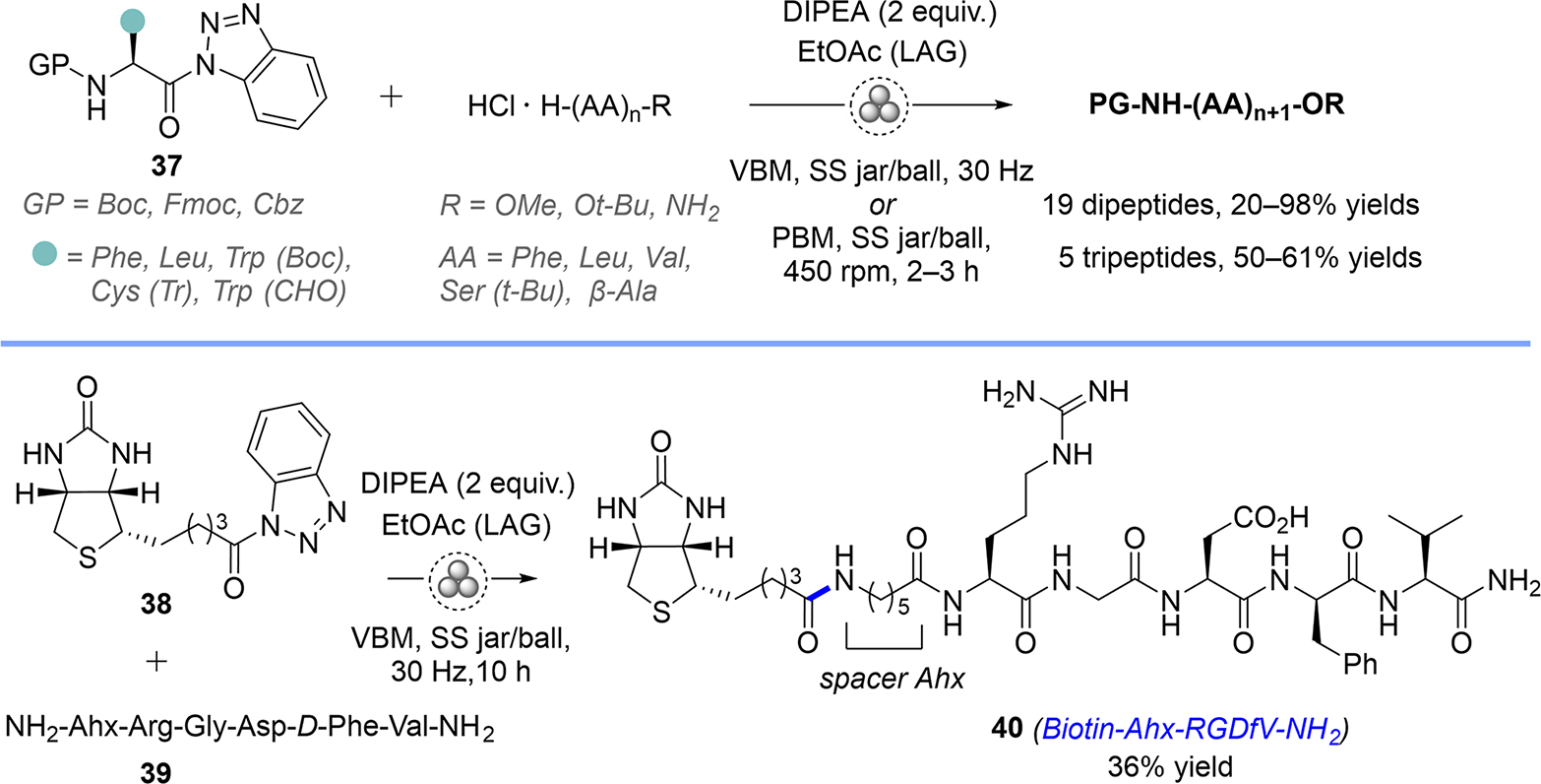
Synthesis of Di- and Tripeptides by Using N-Acyl Benzotriazole Carboxylates^a^

a Ahx = aminohexanoyl spacer.

LAG conditions proved advantageous for the synthesis of tripeptides, which were obtained in moderate 50–61% yields across various amino acid combinations. The majority of di- and tripeptides were isolated as pure compounds by precipitation in water. A comparison of green chemistry metrics for the mechanochemical process versus similar solvent-based procedures showed improved PMIs and RME metrics, alongside better yields, and faster reaction times.

To demonstrate the functionalization of biomolecules, the authors synthesized the biotinylated peptide **40** by coupling a biotin *N*-acyl benzotriazole derivative **38** with a solid-phase synthesized pentapeptide **39** (Scheme 16). Extended milling of compounds **38** and **39** for 10 h in the presence of DIEA and ethyl acetate led to almost complete conversion to the target product **40**. However, despite the high conversion, the isolated yield of **40** was only 36% after precipitation from ethyl acetate and subsequent purification by preparative HPLC to remove residual unreacted peptide.

From a safety standpoint, no explosive behavior was observed for 1*H*-benzotriazole, although its relatively high exothermic decomposition energy (1590 J·g^–1^) with decomposition onset above 300 °C warrant attention.^76^ Accordingly, *N*-acyl benzotriazole derivatives are considered safer to handle than the corresponding azido- or nitro-substituted analogues. Overall, they offer a favorable balance between stability, reactivity, and operational simplicity, rendering them valuable intermediates for solvent-free peptide bond formation under controlled mechanochemical conditions.

Sulfonamide-type leaving groups were also exploited as activators. Cuccu et al.^77^ reported the use of *N*-acyl saccharin derivatives for the synthesis of formamides, acetamides and propionamides (Scheme 17).

**Scheme 17:**
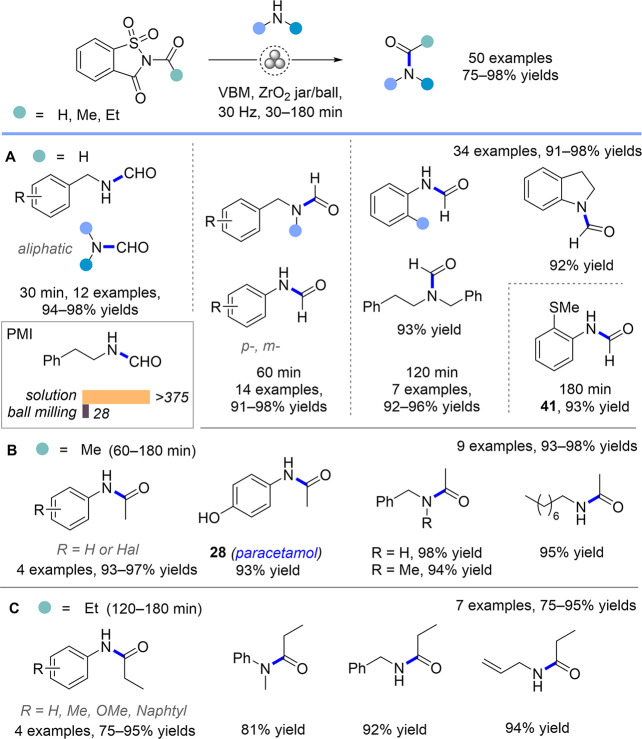
Synthesis of Amides by Using *N*-Acyl Saccharins

A number of formamides was obtained in excellent 91–98% yields by milling N-formyl saccharin with primary and secondary aromatic and aliphatic amines (Scheme 17, A). Primary and secondary aliphatic amines reacted within 30 min, while less nucleophilic anilines and secondary benzylic amines required approximately 1 h to reach full conversion. Sterically hindered ortho-substituted anilines and indoline required 2 h, and *o*-methylthioaniline was the least reactive, affording formamide **41** in 93% yield after 3 h.

The developed formylation methodology was further extended to *N*-acetylation (Scheme 17, B) and *N*-propionylation (Scheme 17, C). A range of aromatic and aliphatic acetamides and propionamides was prepared in good to excellent yields (75–98%), including analgesic paracetamol **28** in 93% yield. The authors also demonstrated a simple purification protocol, involving grinding the crude reaction mixture with moist NaHCO_3_ for 10 min, followed by addition of ethyl acetate, filtration, and concentration under reduced pressure. This procedure converts the saccharin byproduct into its sodium salt, which can be easily isolated by filtration and reused as an acyl group transfer reagent. The mechanochemical approach also reduced the total PMI by nearly 13-fold compared to a similar solution-based method (27.9 vs >375).

#### 2.2.3 Preactivation with Amide Coupling Reagents

To circumvent the need for isolating activated carboxylic acid derivatives, several ″one-jar, two-step″ mechanochemical protocols have been developed. These approaches involve the prior generation of highly reactive acylating agents, such as acyl imidazoles (from CDI), activated esters (from TCT, TBTU, HATU, HDMB), or acyl fluorides (from TFEDMA), followed by their subsequent reaction with amines in the same milling vessel.

CDI is an inexpensive and readily available reagent with versatile applications in mechanochemical organic synthesis.^78^ It generates benign byproducts, such as carbon dioxide and imidazole, which can be easily separated from the resulting amide product. The first CDI-mediated solvent-free amide coupling was reported in 2012 by Verma et al.^79^ (Scheme 18, A). Remarkably, carboxylic acid activation with CDI was carried out manually by mixing and grinding the reactants with a spatula for just 5 min. The subsequent reaction of the resulting acyl imidazole with an amine proceeded rapidly within 10 min in the presence of catalytic imidazole hydrochloride (0.1 mol %) and a minimal amount of water. A series of amides was synthesized in 88–98% yields from various substituted benzoic and phenylacetic acids, as well as aliphatic and benzylic amines (e.g., **42**–**44**). However, sterically hindered *tert*-butylamine was found to be unreactive. The method was also extended to *tert*-butyl alcohol as a substrate in place of a carboxylic acid, affording Boc-protected amine **45** in 88% yield. Product isolation was accomplished by extraction with ethyl acetate.

**Scheme 18:**
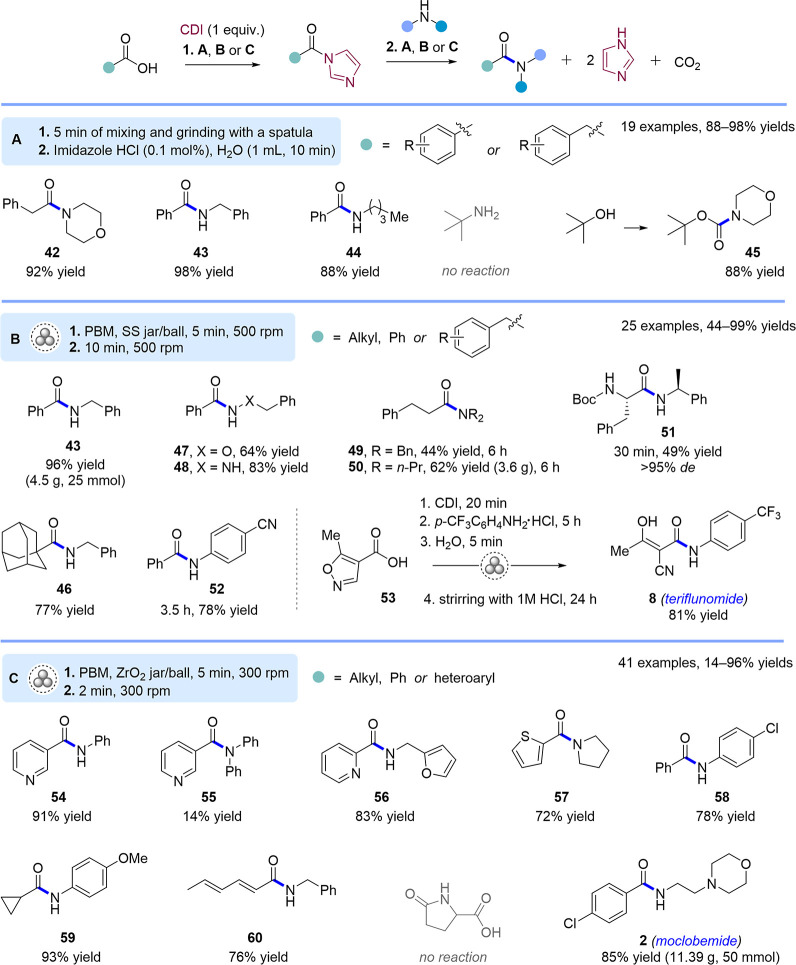
Synthesis of Amides via Prior Activation of Carboxylic Acids with CDI. Selected Examples

In the same year, Métro et al.^80^ reported a highly efficient mechanochemical method for amide bond formation that eliminates the need for any organic solvents (Scheme 18, B). Carboxylic acid activation was achieved by milling with CDI for 5 min, followed by the addition of the amine and further milling for 10 min. Subsequent addition of water to the milling jar and continued milling for another 5 min, followed by filtration, afforded pure amides in 44–99% yields. In terms of substrate scope, a variety of aromatic and aliphatic carboxylic acids were effectively coupled with benzylic and aliphatic amines. Notably, even sterically hindered substrates such as 1-adamantane carboxylic acid reacted efficiently, affording amide **46** in 77% yield. The coupling of benzoic acid with *O*-benzyl hydroxylamine and phenylhydrazine also proceeded smoothly, yielding products **47** and **48** in 64% and 83% yields, respectively.

However, dibenzylamine, a representative secondary amine, showed significantly slower reactivity, requiring 6 h of milling to produce amide **49** in only 44% yield. Importantly, no epimerization was observed in reactions involving enantiopure amines or carboxylic acids, including α-amino acids, as illustrated here with amide **51** (>95% *de*). Furthermore, weakly nucleophilic aniline derivatives were also successfully coupled with benzoic acid. For example, amide **52** was obtained in 78% yield from electron-poor 4-aminobenzonitrile after 3.5 h of milling. Despite the extended reaction time, this was nearly 40 times faster than the corresponding reaction in NMP solution, highlighting the remarkable rate acceleration achieved under solvent-free mechanochemical conditions.

The reactions were typically performed on a 1.5 mmol scale. However, one example was successfully scaled up to multigram quantities, affording 4.5 g of compound **43** in 96% yield. The developed strategy was also applied to the synthesis of the API teriflunomide (**8**). In this case, 5-methyl-4-isoxazolecarboxylic acid (**53**) was first milled with CDI for 20 min, followed by the addition of 4-(trifluoromethyl)­aniline and further milling for 5 h. Subsequent treatment with aq. HCl resulted in isoxazole ring cleavage and provided pure teriflunomide (**8**) in 81% yield, isolated by filtration.

In 2015, the same group reported a comprehensive study on the effects of milling materials and potential contamination from wear in CDI-mediated acylation reaction.^81^ Analysis of the metal content in amide **43** (88% yield, 1.35 mmol scale) using inductively coupled plasma mass spectrometry (ICP-MS) revealed only trace amounts of iron and chromium (366 and 169 ppm, respectively). However, when **43** was dissolved in ethyl acetate and filtered, no metal contaminants were detected. Transition from a planetary mill to a vibratory ball mill resulted in increased contamination with iron and chromium (1400 and 1000 ppm, respectively) due to the higher intensity of collisions between the balls and walls. Changing the jar material to zirconium oxide led to the formation of **43** in 86% yield, although with 12200 ppm of zirconium content. The lowest contamination resulting from wear was observed when using a planetary ball mill with agate jar and balls (less than 100 ppm silicon and 48 ppm iron). However, the yield of **43** was reduced in this case (67%). When a PTFE jar was used, a comparable 69% yield of compound **43** was obtained. However, the product was contaminated with 2400 ppm of PTFE particles. Remarkably, even soft milling material such as rubber can induce the mechanosynthesis of **43** (80% yield, contaminant content of less than 1000 ppm by gravimetric analysis). It was also confirmed that contamination from wear increased with milling time, underscoring the need for optimizing reaction time in ball mill-mediated synthesis. Interestingly, analysis of the metal content in teriflunomide **8**, despite a 5-h grinding time, showed low levels of iron (678 ppm). This was attributed to partial oxidation of metal particles during acidic aqueous treatment, which promoted solubilization and facilitated their removal during filtration. Moreover, additional examples with secondary amines were reported and the protocol was adapted for isolation of liquid amides, since efficient recovery with water was only possible when the product was solid. For example, the synthesis of liquid amide **50** (Scheme 18, B) was scaled up to a 25 mmol scale, and the product was purified by distillation, yielding 3.6 g (62%).

In 2023, Zhu et al.^82^ demonstrated the scalability of the method up to 25 mmol scale using a planetary ball mill (Scheme 18, C). A variety of amides was synthesized in 14–93% yields by coupling of nicotinic (**54**, **55**), picolinic (**56**), thiophene-2-carboxylic (**57**), benzoic (**58**), cyclopropane carboxylic (**59**) and sorbic acids (**60**) with benzylic, aliphatic amines and anilines. Amide coupling of poorly nucleophilic diphenylamine with nicotinic acid demonstrated low efficiency, affording amide **55** in low 14% yield, as well as no reaction was observed with DL-pyroglutamic acid. In the latter case, no amide formation was observed, even with excess amine or acid and prolonged reaction times. As an application, an antidepressant moclobemide (**2**) was synthesized by coupling of *p*-chlorobenzoic acid with 2-morpholinoethan-1-amine and isolated in 85% yield (11.39 g) after washing with water and filtration.

The versatility of CDI-mediated mechanochemical amidation has also been leveraged for the synthesis of more complex biomolecules. For instance, Wehbe et al.^83^ employed this strategy to prepare sericin-derived lipopeptide surfactants (Scheme 19).

**Scheme 19:**
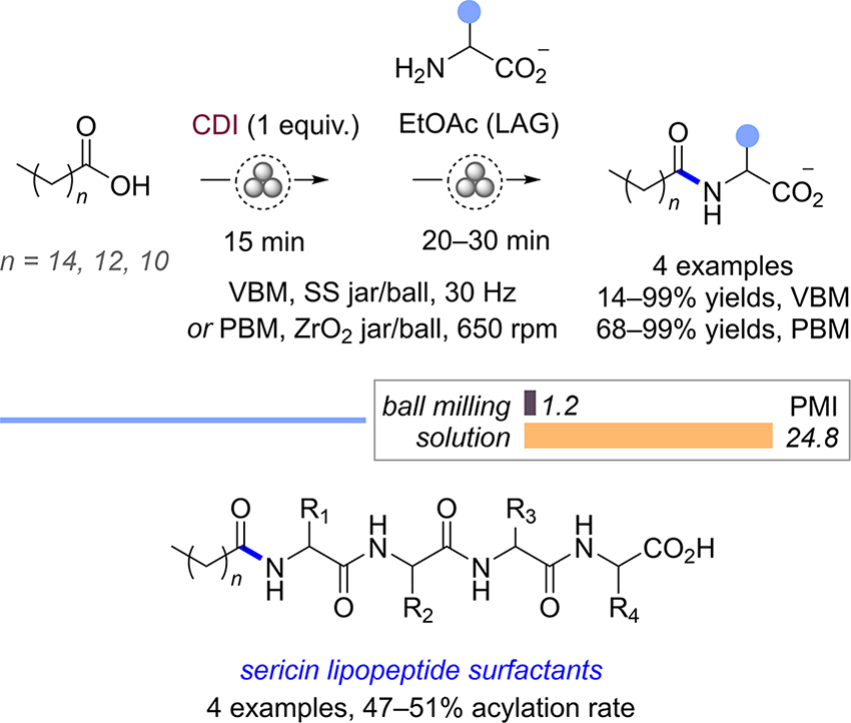
Synthesis of *N*-Acyl AAs and Lipopeptide Surfactants

The reaction conditions were first optimized using amino acids (leucine, glycine, serine, and aspartic acid) under vibrational and planetary ball milling, showing high 68–99% conversion rates to the corresponding *N*-acyl amino acids. Subsequently, the optimized protocol was extended to the acylation of silk sericin peptides, leading to the formation of lipopeptide surfactants with 47–51% acylation rate. Remarkably, the developed mechanochemical approach exhibited a significantly lower PMI compared to the conventional Schotten–Baumann acylation with acyl chlorides in aqueous solution (1.2 vs 24.8), advancing the greener synthesis of biomass-derived amphiphilic molecules.

Further extension of the CDI methodology toward synthesis of hydroxamic acid derivatives was presented by Mocci et al. (Scheme 20).^84^

**Scheme 20:**
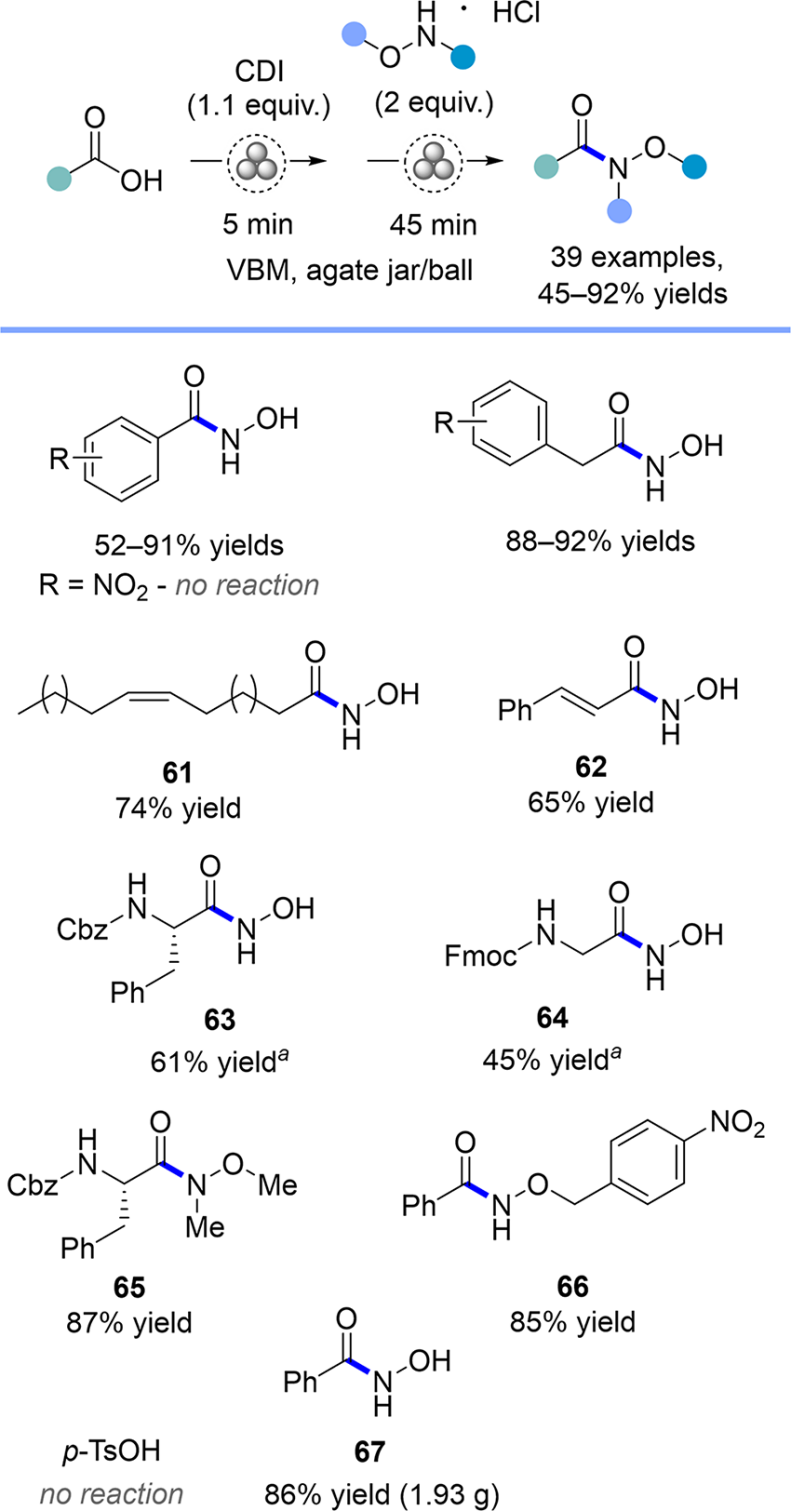
Synthesis of Hydroxamic Acid Derivatives. Selected Examples

a Milling time 200 min.

The mechanochemical approach allowed to overcome a solubility issue with hydroxylamine hydrochloride, which is almost insoluble in organic solvents. Additionally, a significant simplification of isolation protocol was demonstrated, involving an additional 3 min grinding of the final product for 3 min with silica gel, followed by fast and simple filtration on a short silica pad. Regarding the reaction scope, various aromatic acids bearing either electron-donating or electron-withdrawing groups on the aryl ring afforded hydroxamic acids in 52–91% yields. Cyano-substituted substrates showed reduced reactivity (52% yield), while nitro-substituted acids were unreactive. In general, high 88–92% yields were obtained in the reaction of phenylacetic acid derivatives with hydroxylamine. The protocol was applicable to oleic (**61**) and cinnamic acids (**62**). *N*-Protected amino acids required prolonged milling time (200 min) to afford hydroxamic acids **63** and **64** in 61% and 45% yields, but without loss of stereochemical integrity of the stereocenters. The synthesis of *O*-alkyl and *O,N*-dialkyl hydroxamates **65** and **66** proceeded smoothly, affording the products in 87% and 85% yields, respectively. Notably, no reaction occurred with *p*-toluenesulfonic acid (*p*-TsOH). The scalability of the developed methodology was demonstrated by the synthesis of 1.93 g of hydroxamic acid **67** in 86% yield.

Beyond the examples discussed, CDI has also been applied to the mechanochemical synthesis of cross-linked glycopeptides,^308^ hydantoins,^309–311^ carbamates,^312^ and ureas,^305,308^ as well as to the preparation of silicon-based biohybrid materials.^313^

In 2015, Duangkamol et al.^85^ reported alternative two-step mechanochemical methodology through activation of carboxylic acid with 2,4,6-trichloro-1,3,5-triazine (TCT) and a catalytic amount (10 mol %) of PPh_3_ (Scheme 21). The reaction was carried out in a glovebag filled with nitrogen by manual grinding of acid with TCT, PPh_3_, K_2_CO_3_ and dichloromethane as a LAG additive (*η* = 1.5 mL·mg^–1^) for 10–20 min, followed by addition of amine and additional manual grinding for 20 min. The authors proposed that activation proceeds through *in situ* formation of a triazinylphosphonium intermediate from PPh_3_ and TCT, which then reacts with the carboxylic acid to generate the activated 1,3,5-triazinol ester **A**.

**Scheme 21:**
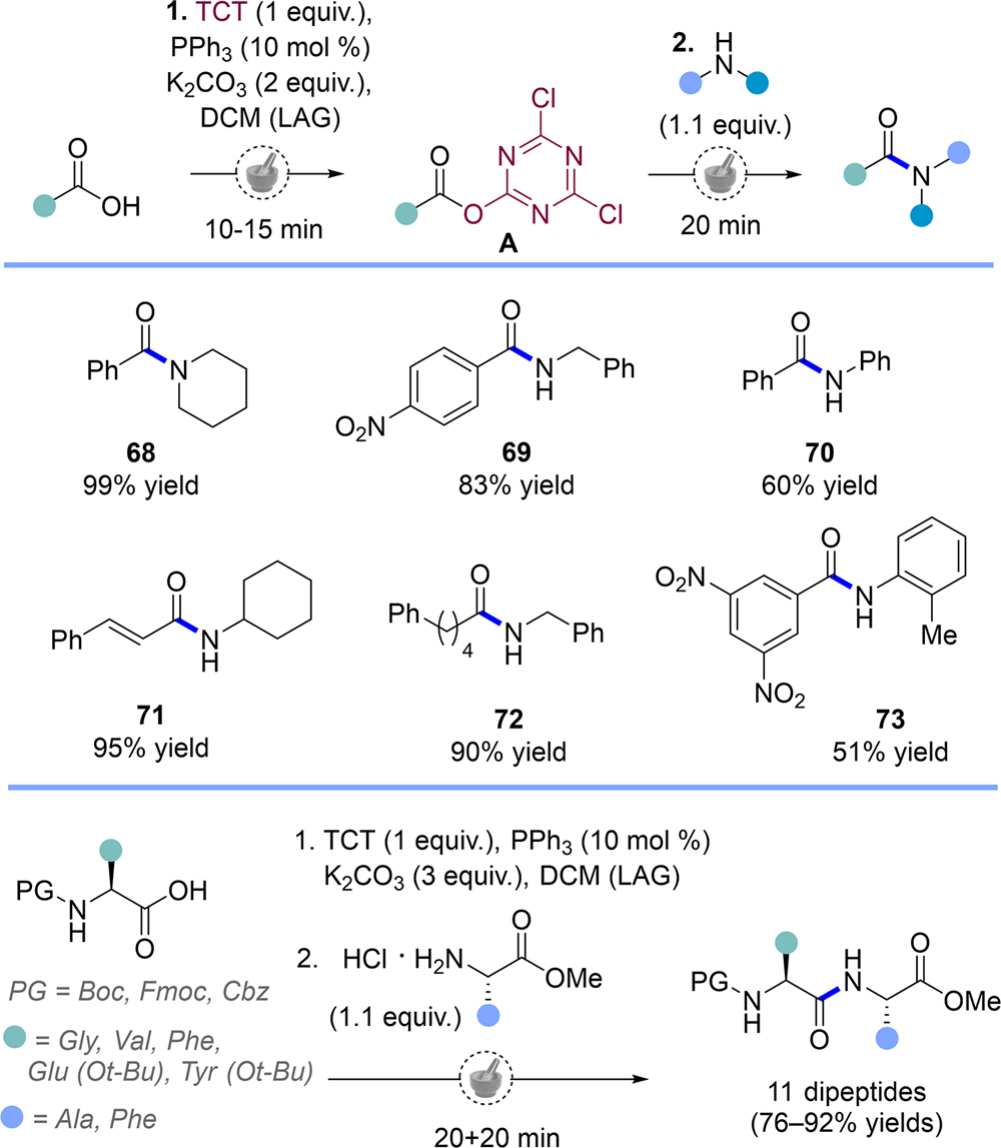
Synthesis of Amides and Dipeptides via TCT/PPh_3_-Mediated Activation of Carboxylic Acids

The reaction of aromatic acids with aliphatic (**68**) and benzylic (**69**) amines proceeded smoothly, while its coupling with the poor nucleophilic aniline was less effective (60% yield of **70**). For comparison, the same amide **70** was obtained in 93% yield by using the CDI-mediated approach.^80^ Cinnamic and 5-phenylpentanoic acid delivered high 95% and 90% yields of amide products **71** and **72**, respectively. However, the reaction of 3,5-dinitrobenzoic acid with sterically hindered 2-methylaniline led to a moderate 51% yield of the product **73**. The coupling of Fmoc, Cbz and Boc-protected α-amino acids with α-amino esters hydrochlorides was also successful, although required slightly longer milling time for the first step (20 min). As a result, 11 dipeptides were synthesized in 73–92% yields with no epimerization detected. Although the developed protocol allows to prepare a variety of amides and dipeptides in good yields, it requires the use of glovebox filled with nitrogen, as well as column chromatography purification of the amides from PPh_3_ and TCT byproducts, in contrast to readily separable side products derived from CDI reagent.

In 2024, Zhao et al.^86^ reported a two-step protocol involving the preactivation of carboxylic acids through the mechanochemical generation of acyl fluorides, using the thermally stable and nonexplosive reagent 1,1,2,2-tetrafluoroethyl-*N,N*-dimethylamine (TFEDMA, Scheme 22). The activation step is completed within 20 min, and the resulting acyl fluorides can either be isolated or used directly in a one-pot deoxyfluorination–coupling sequence. Notably, the reaction of acyl fluorides with amines proceeds efficiently without the need for an additional base. This efficiency is attributed to the proposed trapping of the generated HF by the carbonyl oxygen of the forming amide.

**Scheme 22:**
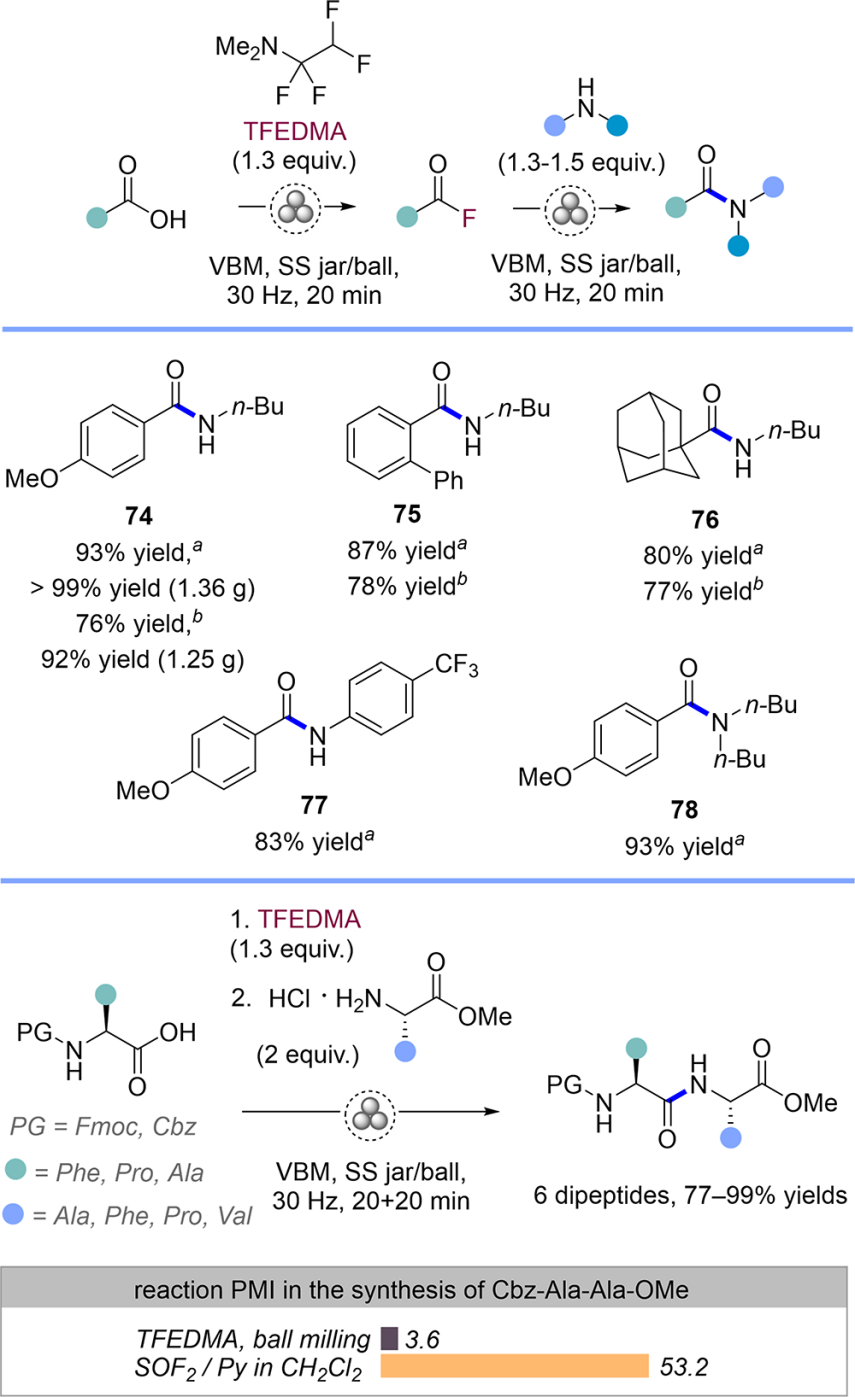
Synthesis of Amides and Dipeptides via TFEDMA-Mediated Deoxyfluorination of Carboxylic Acids, Selected Examples

a Synthesized from the corresponding acyl fluoride.
b Synthesized from the corresponding carboxylic acid in one-pot.

The coupling of *n*-butylamine with preformed acyl fluorides bearing aryl or sterically hindered groups afforded amides **74**, **75**, and **76** in high yields of 93%, 87%, and 80%, respectively. The same amides were also synthesized using the one-pot deoxyfluorination-coupling approach, though with slightly lower yields of 76%, 78%, and 77%, respectively. Additionally, a gram-scale synthesis of amide **74** was demonstrated using both protocols, yielding 1.36 g (99%) via the two-step process and 1.25 g (92%) via the one-pot method. Notably, the product was purified by recrystallization, eliminating the need for column chromatography. The developed mechanochemical conditions were further applied to reactions involving acyl fluorides, electron-poor aniline derivatives or secondary amines, successfully affording amides **77** and **78** in high yields of 83% and 93%, respectively. Moreover, TFEDMA-mediated conditions enabled the synthesis of various dipeptides in yields ranging from 77% to 99%, with no epimerization observed.

A comparative analysis between the mechanochemical approach and a conventional batch reaction in solution (Cbz-Ala-Ala-OMe synthesis using SOF_2_ and pyridine in dichloromethane) revealed advantages of the mechanochemical method. The latter offered a significantly shorter reaction time (40 min vs 2 h) and nearly 20-fold improvement in the reaction PMI (3.6 vs 53.2), although it showed a slightly lower atom economy (AE: 54% vs 39%) due to the lower molecular weight of SOF_2_ compared to TFEDMA. Nonetheless, isolation of the amide products generally required extractive workup with ethyl acetate or dichloromethane, or purification by column chromatography.

In 2024, Wróblewska et al.^87^ reported the use of uronium (aminium) coupling agents for the preactivation of *N*-Boc-protected amino acids in peptide synthesis (Scheme 23).

**Scheme 23:**
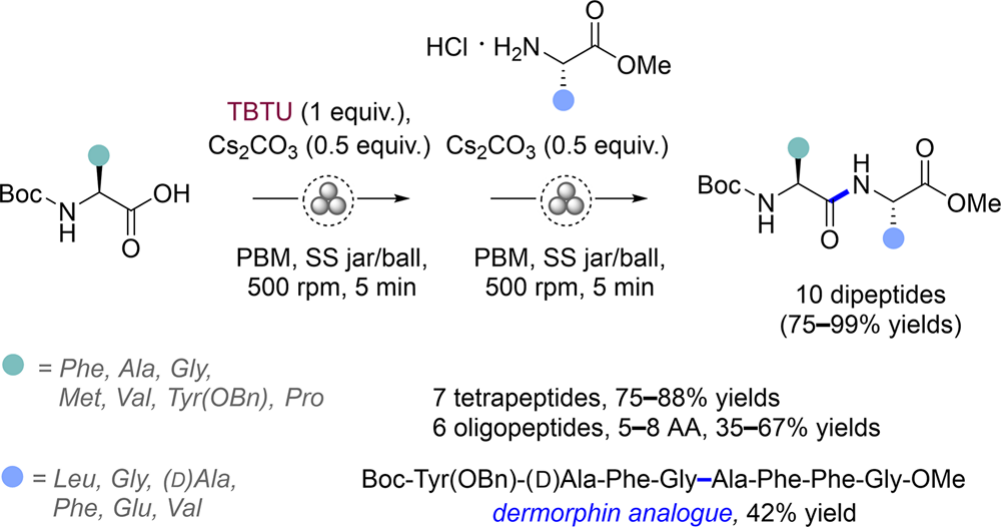
Synthesis of Peptides Using TBTU as a Coupling Reagent

In the model reaction between Boc-phenylalanine and leucine methyl ester, TBTU was identified as the most efficient coupling reagent, outperforming HATU and HDMB. Among the bases tested, Cs_2_CO_3_ delivered higher yields than organic base DIEA or K_2_HPO_4_. The optimized protocol involved milling the *N*-Boc amino acid with the coupling reagent and base for 5 min in a planetary ball mill, followed by the addition of the amino acid ester hydrochloride and the second portion of the base, with continued milling for another 5 min. Using this method, ten dipeptides were synthesized in yields ranging from 75–99%. The products were isolated via extractive workup and chromatographic purification.

Several tetrapeptides and longer oligopeptides (comprising 5–8 amino acids) were also prepared in 35–88% yields, including an analogue of dermorphin, a natural opioid peptide from South American frogs. These longer sequences required extended milling times of up to 40–60 min to achieve complete coupling. Diastereomeric purity of the tetrapeptides, particularly those formed via C-terminal residues like Phe, Val, Leu, and d-Ala, was assessed by HPLC, revealing notable epimerization with diastereomeric ratios ranging from 74:26 to 93:7. The role of Cs_2_CO_3_ was further explored using solid-state NMR, indicating that, beyond acting as a base, it also promotes activation of substrates and intermediates by enhancing the nucleophilicity of amino acid anions and forming CsBF_4_. It was noting that benzotriazole-based coupling reagents such as TBTU may be potentially explosive and pose health hazards.^88,89^

### 2.3 Use of Amide Coupling Reagents for *In Situ* Activation

The preceding section detailed mechanochemical strategies utilizing activated carboxylic acid derivatives that are either preformed or sequentially generated prior to reaction with amines. An alternative and attractive approach is the direct, *in situ* activation of carboxylic acids in the presence of amines by using stoichiometric amide (peptide) coupling reagents.^3,4,57^ An ideal coupling reagent must satisfy multiple chemical and practical criteria: it should exhibit broad substrate scope, effectively form challenging amide bonds (e.g., involving sterically hindered acids or weakly nucleophilic amines), and minimize epimerization, particularly crucial in peptide synthesis. Practical considerations include shelf stability, low molecular weight (to ensure high atom economy), low cost, generation of easily removable byproducts, and compatibility with process safety requirements such as low toxicity, thermal stability, and minimal shock sensitivity.^88^ The latter is especially important for reactions conducted in a ball mill, where mechanical impact can amplify safety risks.^90^ Given the extensive list of desired attributes, no single reagent can fulfill all criteria perfectly, leading to the development of numerous coupling reagents, primarily carbodiimides, uronium (aminium), and phosphonium salts, traditionally utilized in solution-phase chemistry. In this section, the mechanochemical adaptations of these widely employed coupling reagents are discussed according to their respective classes.

#### 2.3.1 Carbodiimide-Mediated Transformations

Carbodiimides are a widely used class of coupling reagents for amide bond formation, valued for their relatively low cost and ability to promote coupling without the need for an added base.^4^ Mechanistically, they activate carboxylic acids by forming a reactive *O*-acylisourea intermediate, which subsequently reacts with an amine to generate the desired amide. The reaction is driven by the formation of a stable urea byproduct, which is typically easy to separate from the amide. For example, the urea derived from 1-ethyl-3-(3-(dimethylamino)­propyl)­carbodiimide (EDC) is water-soluble and can be efficiently removed by aqueous workup. EDC is commonly supplied as its crystalline hydrochloride salt (EDC·HCl), which is shelf-stable, easy to handle, and preferred from a process safety standpoint.^88^ However, it should be handled with care, as carbodiimides are known as skin sensitizers and allergens.^91,121^ Due to its advantages, EDC is the most popular coupling reagent for large-scale (over 100 mmol) amide synthesis.^4^ Reflecting its prominence in solution-phase chemistry, it has also become the most frequently employed reagent in mechanochemical amide coupling.

The first mechanochemical amide coupling mediated by EDC was reported by Štrukil et al. in 2012 (Scheme 24).^92^

**Scheme 24:**
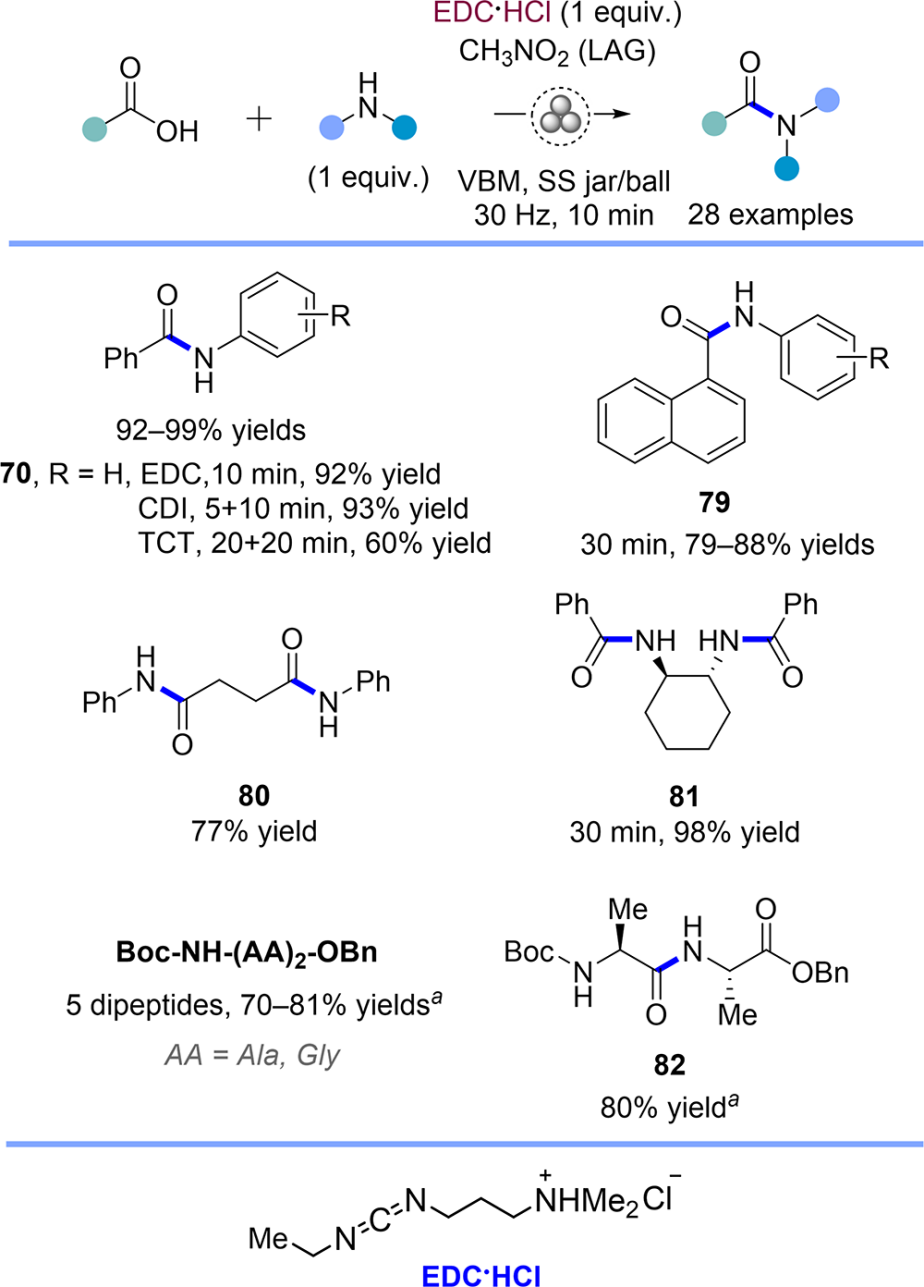
Synthesis of Amides and Dipeptides Using EDC as a Coupling Reagent. Selected Examples

a The reactions were performed in the presence of DMAP (2 equiv), NaCl (20 equiv), milling time 3 h.

A series of *N*-aryl benzamides was synthesized by milling benzoic or 1-naphthoic acids with aniline derivatives in the presence of stoichiometric EDC and nitromethane (η = 0.25 μL·mg^–1^) as a LAG additive. The reaction proceeded rapidly, reaching completion within just 10 min. Subsequent suspension of the crude mixture in water followed by stirring for 15 min led to precipitation of the pure amide products, which were isolated by filtration in 79–99% yields (**70**, **79**). For the synthesis of benzamide **70**, the EDC-mediated protocol was as efficient as the two-step CDI-based approach^80^ (92% vs 93% yield) and outperformed the TCT-mediated method^85^ (60% yield). The method was also successfully applied to the synthesis of bisamides **80** and **81**, starting from succinic acid and (1*R*,2*R*)-cyclohexyldiamine, respectively. The coupling of *N*-Boc-protected glycine or alanine with their benzyl esters required the addition of DMAP as a base and NaCl as a grinding auxiliary, as well as extended milling (3 h), to obtain the desired dipeptides in 70–81% yields (e.g., dipeptide **82**). No racemization was observed. Overall, the methodology is fast and features a straightforward isolation protocol. However, hazardous nitromethane as a LAG additive presents safety concerns. Moreover, the synthesis of dipeptides was demonstrated on a small scale (<100 mg), with extended reaction times (3 h), a relatively low milling load (ML < 83 mg·mL^–1^), and the use of toxic DMAP as a required additive.

In 2016, Porte et al. reported an improved methodology addressing these shortcomings.^93^ The procedure employed EDC in combination with ethyl cyanohydroxyiminoacetate (Oxyma) as an epimerization suppressant, nontoxic NaH_2_PO_4_ as a base, and ethyl acetate as a green LAG additive (Scheme 25). After 10 min of milling followed by extraction workup, various dipeptides were obtained in 63–91% yields. PTFE was selected as the material for both the milling jar and balls to avoid sparking that can occur with stainless steel, enabling the safe use of low-flash-point LAG additives. The presence of a liquid additive proved essential: in its absence, conversions dropped to 60% and standard deviation in the reaction mixture composition increased to 38%, indicating poor mixing. Polar aprotic solvents such as DMF, GVL, and ethyl acetate were the most effective additives, outperforming dodecane, water, and glycerol.

**Scheme 25:**
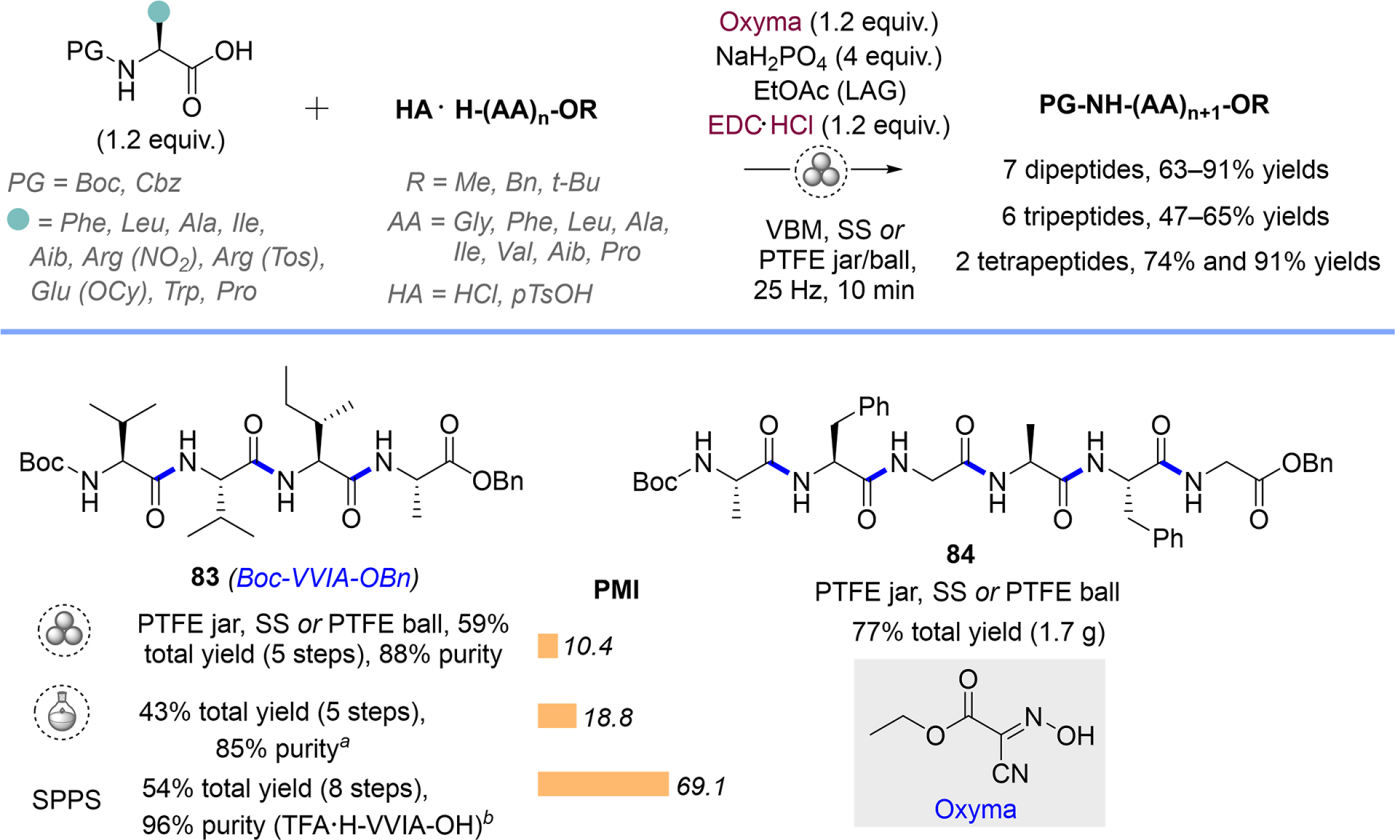
Synthesis of Di-, Tri-, Tetra- and Hexapeptides Using EDC as a Coupling Reagent

a Reaction conditions: Boc-AA–OH (1.2 equiv), Oxyma (1.2 equiv), DIEA (1.2 equiv), EDC (1.2 equiv), DMF, r.t.
b Reaction conditions: Fmoc-A-Wang resin, Fmoc-AA–OH (5 equiv), DIC (5.0 equiv), Oxyma (5.0 equiv), DMF, microwave heating or r.t.

The methodology also proved scalable. At a high milling load (ML = 432.5 mg·mL^–1^), 1.5 g of Boc-Phe-Gly-OMe was obtained in 66% yield, and 2.2 g of Boc-Leu-Leu-OBn was isolated in 88% yield. The successful synthesis of sterically hindered dipeptides, such as Cbz-Aib-Val-OMe and Cbz-Aib-Aib-OMe in 89% and 73% yields, respectively, highlighted the method’s efficiency with sterically hindered amino acids. To access longer peptides, Boc-dipeptides were first quantitatively deprotected using gaseous HCl, then coupled with Boc-protected α-amino acids to give tripeptides in moderate yields (47–65%). This strategy was extended to produce tetrapeptides Boc-Pro-(Leu)_3_-OBn and Boc-(Leu)_4_-OBn in 74% and 91% yields, respectively.

In 2017, the same group applied their mechanochemical methodology to the synthesis of the tetrapeptide Boc-VVIA-OBn (**83**), a potential inhibitor of amyloid β-protein relevant to Alzheimer’s disease therapy.^94^ The target compound was assembled through five alternating coupling and deprotection steps, yielding the tetrapeptide in 59% overall yield and 88% purity. For comparison, the tetrapeptide was also synthesized using two conventional approaches: solution-phase synthesis in DMF and solid-phase peptide synthesis (SPPS). In terms of overall yield, ball milling performed comparably to SPPS (59% vs 54%) and outperformed the solution-based method (43%). Moreover, the coupling reactions were significantly faster under mechanochemical conditions, requiring only 20 min per step, compared to 3 h for the solution-phase synthesis. In terms of environmental impact, the ball milling protocol generated considerably less waste than both the solution and solid-phase methods, with SPPS exhibiting the highest PMI. However, SPPS provided the highest purity (96%), while the ball-milled product achieved 88%. Another application was demonstrated in the gram-scale synthesis of hexapeptide **84**, achieved by coupling two tripeptide precursors, HCl·H-Ala-Phe-Gly-OBn and Boc-Ala-Phe-Gly-OH.^95^

The EDC/Oxyma protocol also proved effective for the challenging peptide couplings, such as synthesis of dipeptides **85**–**87** from sterically hindered l-proline and pyrrolidine-2,5-dicarboxylic acid derivatives (Scheme 26, A).^96^ Furthermore, the same system demonstrated high efficiency in peptide couplings involving epimerization-prone or sterically hindered amino acids at the C-terminus, such as phenylglycine, cysteine, and valine (Scheme 26, B).^97^ In all cases, excellent yields (84–98%) were obtained, with negligible epimerization observed (>99% *de*; a detailed discussion of stereochemical integrity is provided in Section 5).

**Scheme 26:**
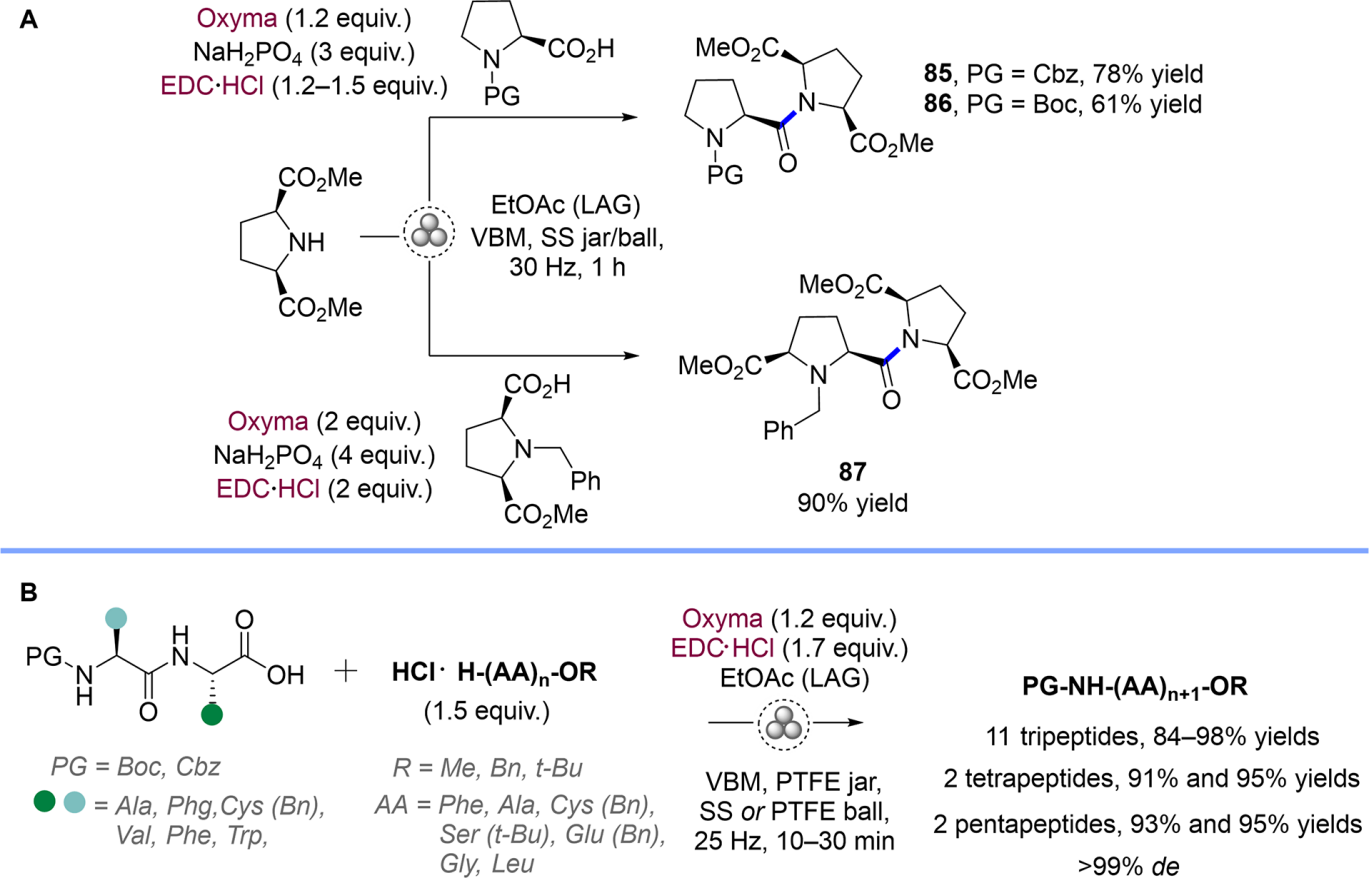
Challenging Peptide Couplings Using EDC/Oxyma

In 2017, Landeros and Juaristi^98^ introduced Mg–Al hydrotalcite (HT-S) as an efficient, inexpensive, and environmentally friendly inorganic base for dipeptide synthesis (Scheme 27, method A). By milling various amino acid methyl ester hydrochlorides with *N*-protected amino acids in the presence of EDC and hydroxybenzotriazole (HOBt) as an epimerization suppressant, a series of dipeptides was obtained in 70–89% yields. Although the catalytic performance of recycled HT-S declined after four cycles, it could be fully restored through calcination followed by rehydration. In 2020, a similar method employed nanocrystalline hydroxyapatite as a biocompatible and reusable inorganic base (Scheme 27, method B).^99^

**Scheme 27:**
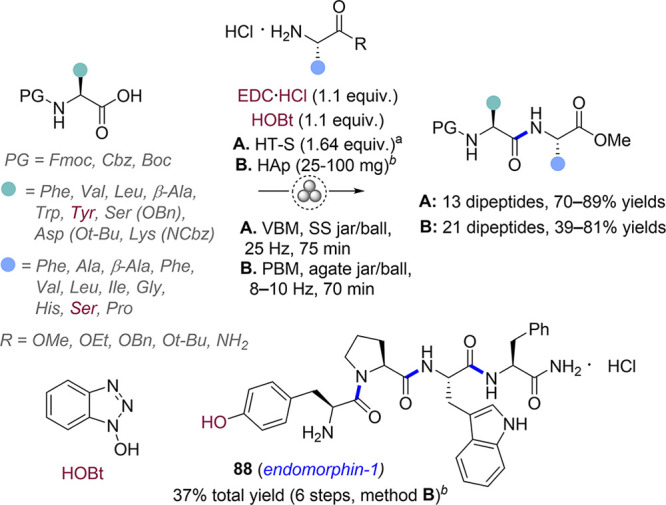
Synthesis of Dipeptides Using EDC/HOBt as a Coupling System^a^

a Reaction conditions: 0.25 mmol scale, EDC (1.2 equiv), HOBt (1.2 equiv), milling time 2 h, in the presence of Mg–Al hydrotalcite (HT-S, method A).
b Hydroxyapatite as a base (HAp, method B).

Notably, this protocol enabled the coupling of phenylalanine with tyrosine and serine, both bearing unprotected hydroxyl groups, yielding the corresponding dipeptides in moderate yields of 66% and 59%, respectively. To demonstrate the practical applicability of the developed methodology, the bioactive tetrapeptide endomorphin-1 (**88**), an endogenous ligand of the μ-opioid receptor, was synthesized via six alternating coupling and deprotection steps. The target peptide was isolated in 37% overall yield with excellent purity (94%).

In 2021, Shou et al.^100^ reported a notable application of the EDC/HOBt-mediated method in the final step of synthesizing the antiparasitic API (*R*)-praziquantel (**90**, Scheme 28). After evaluating several amide coupling reagents and additives, the combination of EDC and HOBt delivered the best result, affording compound **90** in 84% yield with excellent preservation of enantiomeric purity (99.7% *ee*). The protocol involved a preactivation step in which cyclohexanecarboxylic acid was milled with EDC and HOBt for 30 min, followed by the addition of amine **89** and further milling for an additional 30 min. This sequence provided an efficient one-pot approach for the final construction of **90** without the use of basic additives. The methodology was successfully scaled to a 50 mmol reaction, yielding the API in 80% isolated yield and 99.3% *ee* after recrystallization.

**Scheme 28:**
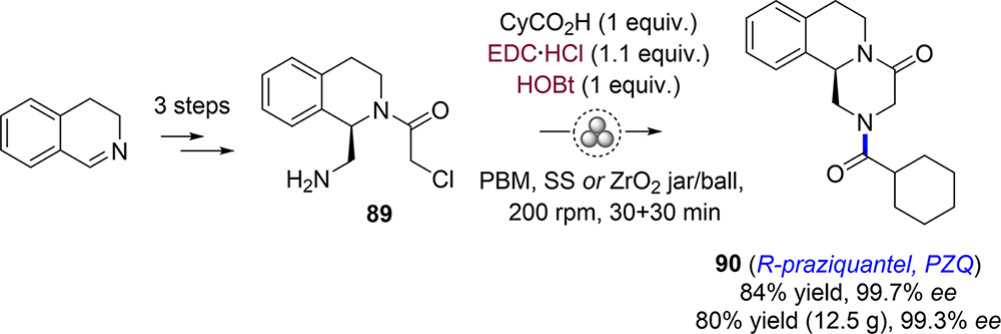
Synthesis of Antiparasitic Drug (*R*)-Praziquantel Using EDC/HOBt

A potential safety concern in these studies^98–100^ is the use of HOBt, which is known to exhibit explosive properties under mechanical stress.^101^ However, no accidents were reported.

The EDC-mediated approach exhibits excellent functional group tolerance and is well-suited for synthesizing amides from carboxylic acids and amines bearing unprotected hydroxyl groups (Scheme 29).^102^ In this respect, it outperforms more reactive uronium-based coupling reagents such as COMU and TCFH (see Section 2.3.2), which are prone to forming byproducts. This advantage was demonstrated through the successful coupling of 4-(hydroxymethyl)­benzoic acid, *N*-Boc-l-serine, *N*-Boc-l-tyrosine, and lithocholic acid with aromatic amines, *N*-Boc piperazine and phenylalanine methyl ester to yield dipeptides **91** and **92**. Notably, sterically hindered and poorly nucleophilic anilines gave lower yields of amides **93**, **94** compared to more reactive *N*-Boc-piperazine (amide **95**). In one example, selective acylation of the amino group in (4-(aminomethyl)­phenyl)­methanol with benzoic acid furnished amide **96** in 85% yield. The retention of unprotected hydroxyl groups in the resulting products enables straightforward functionalization in a step-efficient manner. This was exemplified by the mechanochemical synthesis of an anticancer drug imatinib (**98**). The target API was obtained in 86% overall yield and 99% HPLC purity via generation of intermediate **99** from 4-(hydroxymethyl)­benzoic acid. Importantly, this strategy bypasses the genotoxic benzylic chloride analogue of **99**, employed in solution-phase routes.

**Scheme 29:**
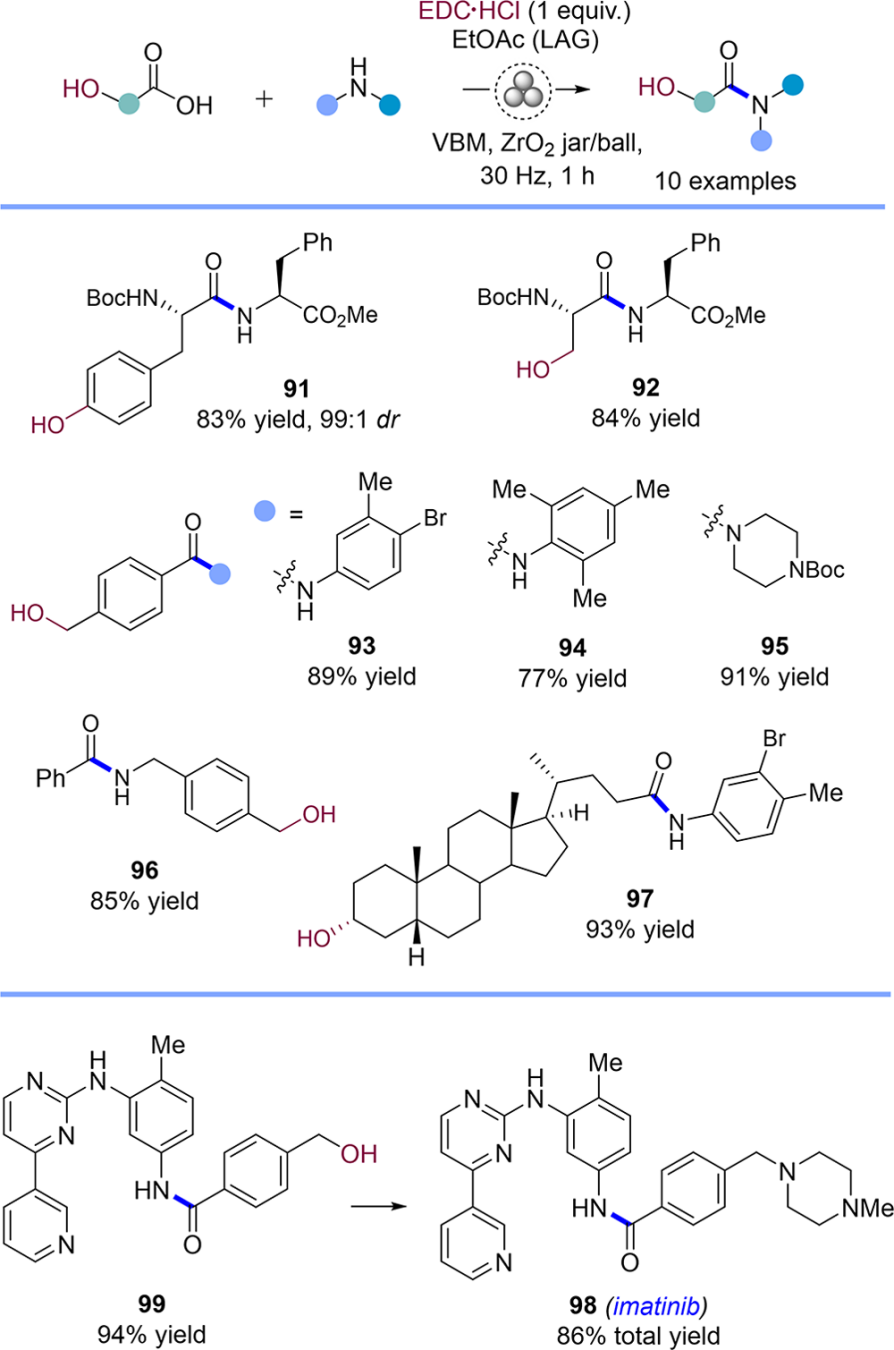
EDC-Mediated Synthesis of Amides and Dipeptides from Substrates with Unprotected Hydroxyl Groups, Selected Examples

In 2024, Cyniak and Kasprzak^103^ reported EDC-mediated synthesis of a polyaromatic amide **100** (Scheme 30).

**Scheme 30:**
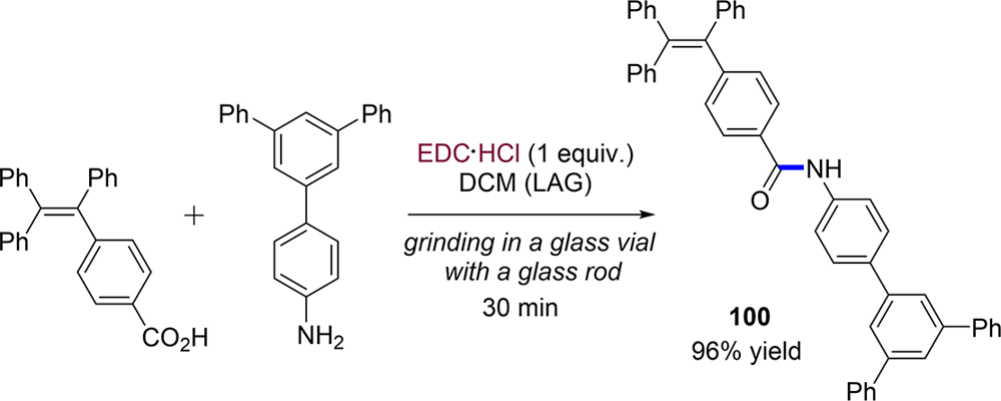
Synthesis of Polyaromatic Amide 100 Using EDC

Interestingly, the reaction was performed by manual grinding of reactants in a glass vial with a glass rod. The reaction was completed in just 30 min with a 96% yield, outperforming the solution-based method, which gave only 48% yield after 170 h. From a green chemistry perspective, the mechanochemical approach drastically reduced solvent use, lowering the PMI for both reaction and solvent by factors of 17 and 81, respectively.

In 2025, Bankar and Jadhav reported the mechanochemical synthesis of the API ivacaftor (**101**), approved for the treatment of cystic fibrosis (Scheme 31).^104^ The target compound was obtained in 70% yield by manual grinding of the starting materials with EDC hydrochloride. In this transformation, EDC outperformed CDI and HATU as coupling reagents, and the addition of methanol as a liquid-assisted grinding (LAG) additive was found to be crucial.

**Scheme 31:**
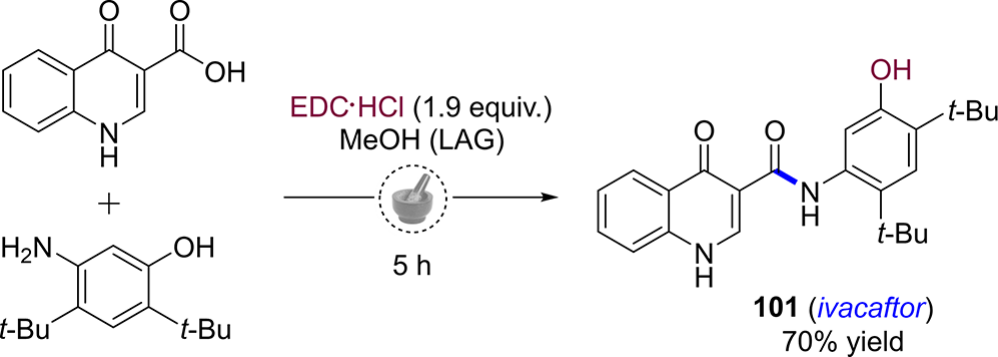
Synthesis of API Ivacaftor by Manual Grinding with EDC Reagent

In 2017, Wróblewska et al.^105^ reported a mechanistic study to elucidate the coupling behavior of EDC under solvent-free mechanochemical conditions (Scheme 32). Using solid-state NMR spectroscopy, X-ray crystallography, and quantum chemical calculations, they demonstrated that EDC exists exclusively in its cyclic form **A** in the solid state.

**Scheme 32:**
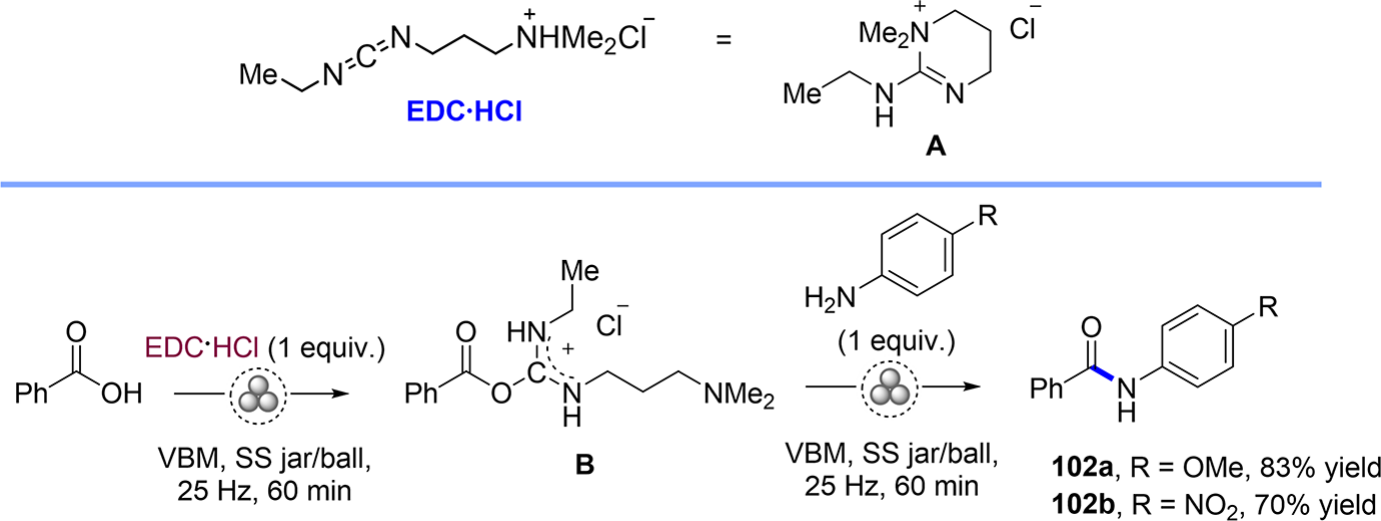
Mechanistic Investigation of the EDC-Mediated Amide Synthesis

Upon grinding with a carboxylic acid, this form undergoes ring opening to generate intermediate **B**. This transformation was linked to the formation of a low-melting phase, facilitating the mechanochemical reaction, as evidenced by differential scanning calorimetry and NMR. The resulting intermediate **B** was subsequently converted to amides (e.g., **102a**,**b**).

In 2023, Atapalkar and Kulkarni reported the adaptation of EDC-mediated amide coupling to extrusion technology, enabling scalable synthesis under continuous-flow conditions (Scheme 33).^106^

**Scheme 33:**
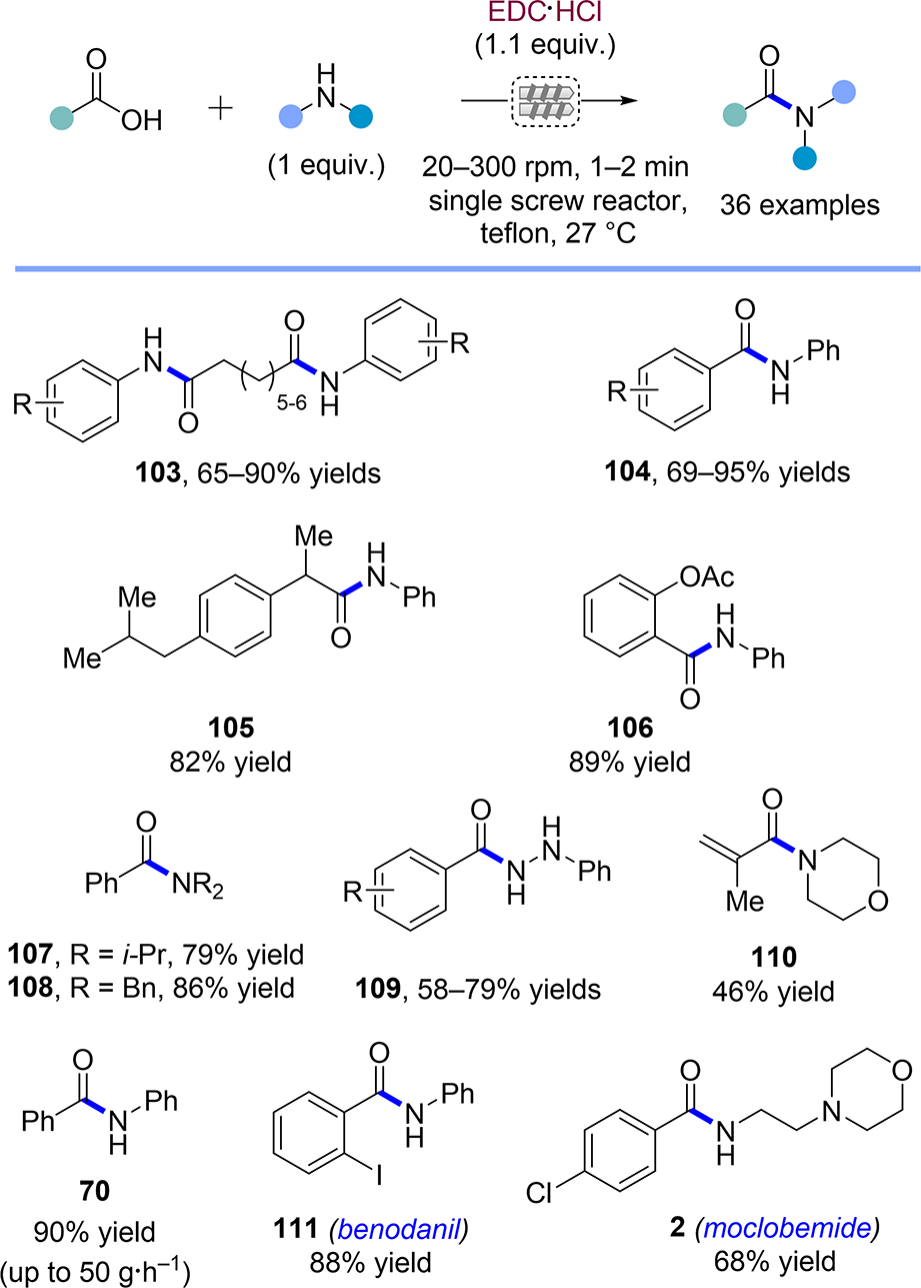
Synthesis of Amides Using EDC Coupling Reagent via Reactive Extrusion. Selected Results

A diverse set of 36 amides was synthesized without the use of solvents and under ambient temperature (27 °C), using a single-screw Teflon extruder with remarkably short residence times of 1–2 min. Various anilines were successfully coupled with long-chain dicarboxylic acids (azelaic and suberic acids), benzoic acid derivatives, ibuprofen, and aspirin, affording amides **103**–**106** in 65–95% yields. Secondary aliphatic amines also reacted efficiently, yielding benzamides **107** and **108** in 79% and 86% yields, respectively. Additionally, *N*-benzoyl phenylhydrazines **109** were prepared. Cinnamic and methacrylic acids were coupled with aniline and morpholine, although methacrylic acid yielded the corresponding amides (e.g., **110**) in only ∼ 50% yields. The utility of this method was further demonstrated through the synthesis of the pharmaceutical moclobemide (**2**) and the fungicide benodanil (**111**), obtained in 68% and 88% yields, respectively. The process was successfully scaled up to 50 g·h^–1^ throughput. For instance, 100 g of amide **70** was produced in nearly 90% isolated yield, an encouraging step toward industrial implementation of continuous mechanochemical manufacturing.

While EDC remains the dominant carbodiimide reagent in this field, in 2024 *N*,*N’*-diisopropylcarbodiimide (DIC) was also demonstrated as an effective coupling agent for continuous peptide synthesis using reactive extrusion (Scheme 34).^107^ Among the different reagents tested, combination of DIC and Oxyma provided quantitative conversion with the lowest percentage of epimerization. The optimized reaction conditions were then applied to the synthesis of a wide range of dipeptides, which were isolated in excellent 84–99% yields after recovery with ethyl acetate and aqueous washings. A chromatographic purification was required only in the case of trityl-protected amino acids. Remarkably, NO_2_-protected arginine methyl ester was almost unreactive when coupled to tryptophan and aspartic acid amino acids (3% and 32% conversions, respectively). To confirm the applicability of the developed approach to longer peptide fragments, two tripeptides, Boc-Ala-Phe-Val-OMe and Cbz-Ala-Phg-Ile-OMe, were synthesized in high 97% and 75% yields. Notably, no epimerization was detected in both cases, even for the coupling of phenylglycine and sterically hindered isoleucine. Metal contamination from the TSE to the product has been assessed, demonstrating much lower metal content than what was obtained in ball mills, and decreasing even lower after purification.

**Scheme 34:**
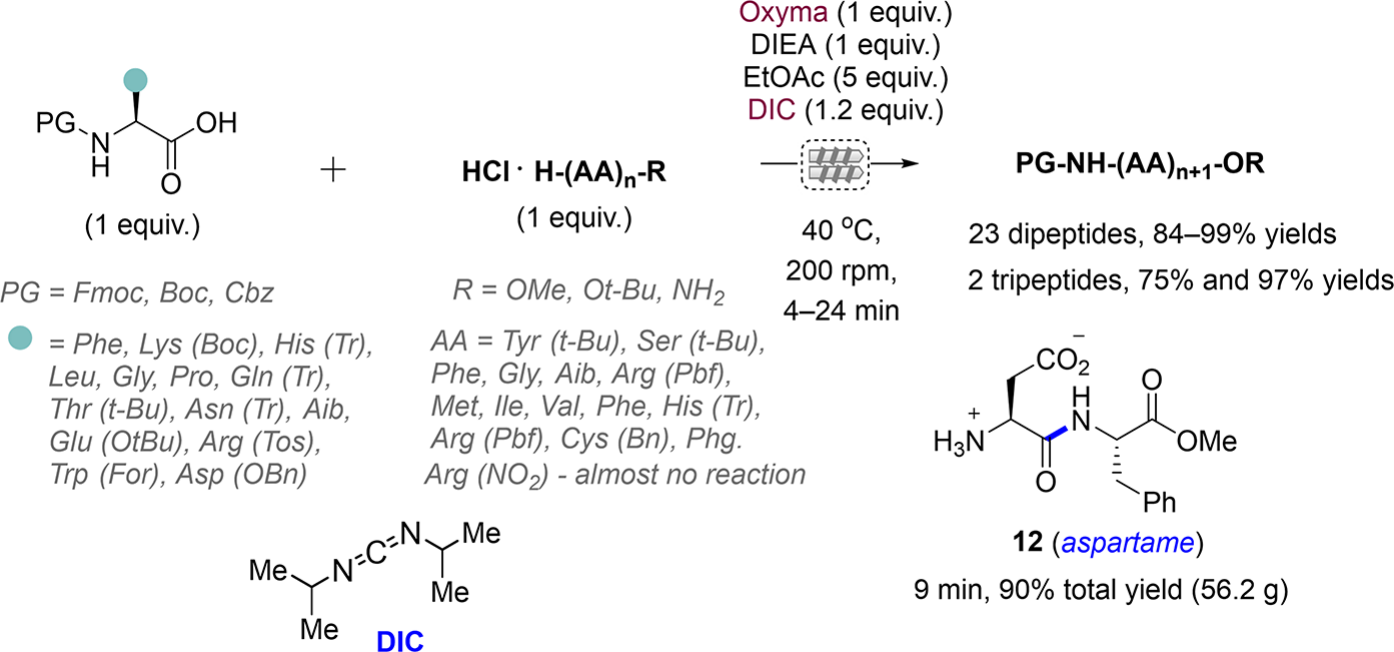
Synthesis of Di- and Tripeptides from Inactivated AAs by Reactive Extrusion

To demonstrate the applicability of the developed protocol, scale-up synthesis of aspartame **12** was performed. Previously, the synthesis of **12** was performed in both a ball mill^59^ and in an extruder,^62^ starting with activated amino acids. In the current work, **12** was synthesized by running the reaction of Boc-Asp­(O*t*-Bu)–OH with HCl·H-Phe-OMe in the extruder in a continuous mode during 8 min and 41 s. Following Boc-deprotection resulted in isolation of 56.2 g of pure aspartame **12** in 90% yield. Additionally, the space time-yield (STY), a metric utilized in examining reactor efficiency and process intensification of a reaction, was estimated to be equal to 4.7·10^6^ kg·m^–3^·day^–1^, which was the highest value obtained so far in flow mechanosynthesis of organic fine chemicals. During optimization, heating in the extruder to remove the Boc group led to formation of the corresponding diketopiperazine byproduct at temperatures above 60 °C. However, no diketopiperazine formation was observed in any of the dipeptides prepared when the temperature was maintained at 40 °C.

#### 2.3.2 Uronium (Aminium) and Phosphonium Reagents

Uronium reagents such as COMU^108^ and TCFH,^109^ which are more reactive than carbodiimides, were introduced to mechanochemistry in 2020 by Dalidovich et al.,^110^ with the aim of facilitating challenging couplings involving sterically hindered carboxylic acids and poorly nucleophilic amines, as well as enabling efficient functionalization of poly­(carboxylic acid)­s (Scheme 35). Both reagents outperformed EDC in the synthesis of amide **112** from Cbz-protected phenylalanine and weakly nucleophilic aromatic amine (benzocaine), achieving yields of 92–96% after 20 min of milling, compared to 87% with EDC. The optimized protocol included ethyl acetate as a LAG additive and K_2_HPO_4_, which functioned both as a base and a precursor for reactive acyl phosphate intermediates.

**Scheme 35:**
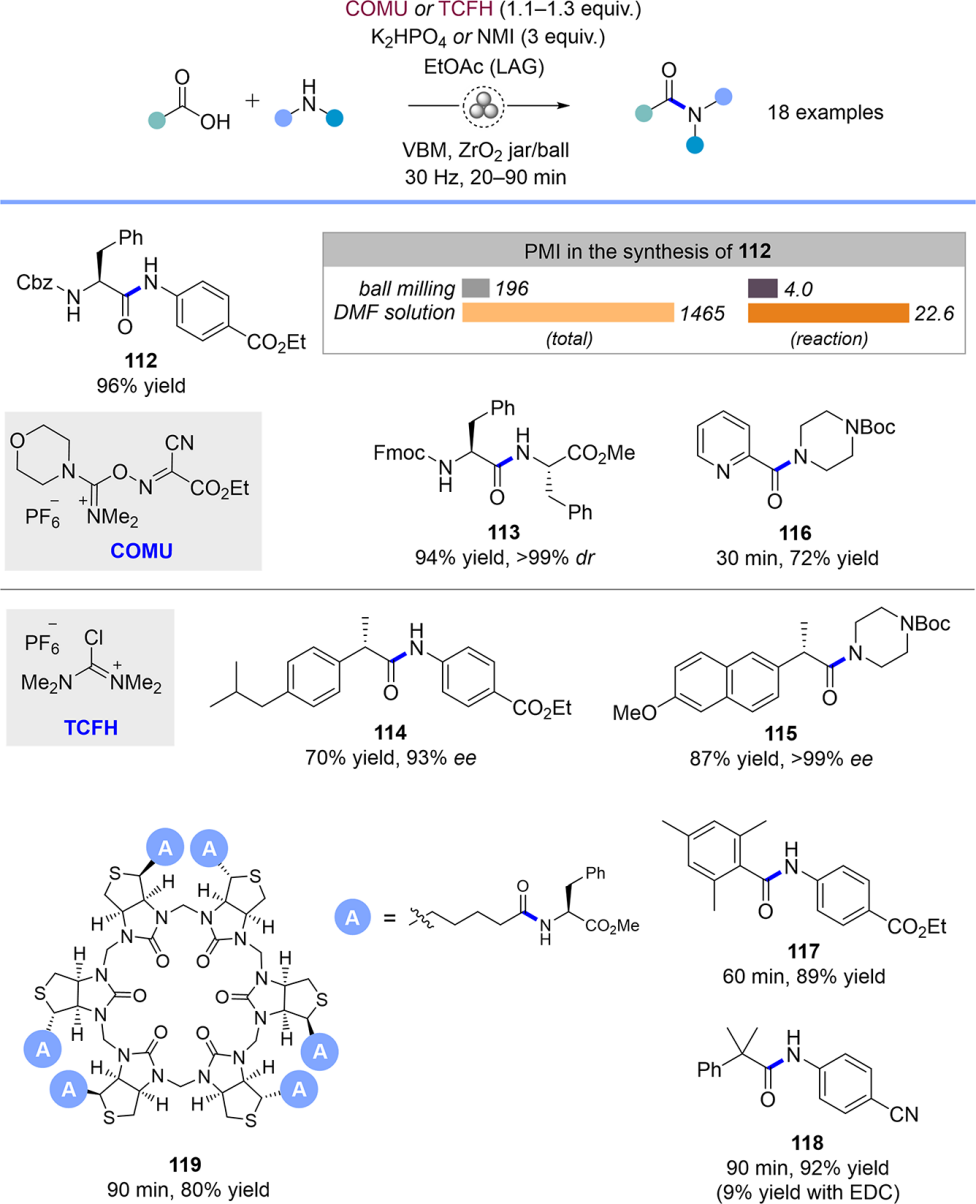
Synthesis of Amides Using COMU and TCFH as Coupling Reagents, Selected Examples

Using COMU and TCFH, a broad range of amides was synthesized in 70–96% yields, including dipeptides (e.g., **113**) and amides derived from pharmaceutically relevant substrates such as (*S*)-ibuprofen (**114**) and (*S*)-naproxen (**115**). No epimerization was detected for amides **113** and **115**, while a slight reduction in optical purity (93% *ee*) was observed for **114**. Solid amides were isolated via simple aqueous workup and filtration. However, oily products required chromatographic purification. The COMU-based protocol was successfully applied to in the synthesis of amide **116**,^111^ a precursor of the psychoactive drug piberaline. TCFH proved more reactive than COMU, especially effective in amidation of sterically hindered mesitoic acid (amide **117**), although requiring a longer reaction time (1 h).

The distinction in reactivity was especially evident in the synthesis of amide **118** from electron-poor 4-aminobenzonitrile and sterically hindered 2-methyl-2-phenylpropanoic acid. Here, the combination of TCFH with *N*-methylimidazole (NMI) provided superior performance, delivering amide **118** in 92% yield after 1.5 h of milling, significantly faster than the corresponding solution-phase reaction (21 h),^112^ and greatly outperforming EDC. As another demanding case, the coupling of biotin[6]­uril with methyl ester of phenylalanine required an exceptionally high coupling efficiency (>99% per step) to obtain the final hexa-amide product in useful yield and purity. Employing TCFH/NMI under prolonged milling (90 min) with ethyl acetate as the LAG additive, hexa-amide **119** was obtained in 80% isolated yield and 99% purity.

Green chemistry metrics highlighted the advantages of this mechanochemical method over solution-based approaches for the synthesis of **112**, including higher yield, much shorter reaction time, simpler isolation protocol, and significantly lower waste production (nearly an 8-fold reduction in total PMI, from 1465 to 196, excluding column chromatography purification in the former case). It also circumvents the instability of COMU solutions in DMF, which are prone to hydrolytic degradation. It is worth noting that both COMU and TCFH can form guanidine byproducts when reacting with amines,^111,102^ which should be considered in reaction planning and product purification.

Cyclic oligopeptides possess unique structural features and diverse biological activities, making them valuable targets in medicinal and material chemistry. However, their synthesis presents significant challenges, particularly due to the entropically disfavored nature of the cyclization step.^113^ Traditional methods often require high dilution conditions in solvents such as DMF to suppress unwanted intermolecular couplings, which leads to slow reaction rates and labor-intensive procedures.

Until recently, the mechanochemical synthesis of cyclopeptides remained elusive. Performing intramolecular cyclization under concentrated solid-state conditions appears counterintuitive compared to the high dilution approach and raises concerns about intermolecular side reactions. Despite this challenge, Duvnjak et al.^114^ demonstrated a successful strategy based on anion templating (Scheme 36).

**Scheme 36:**
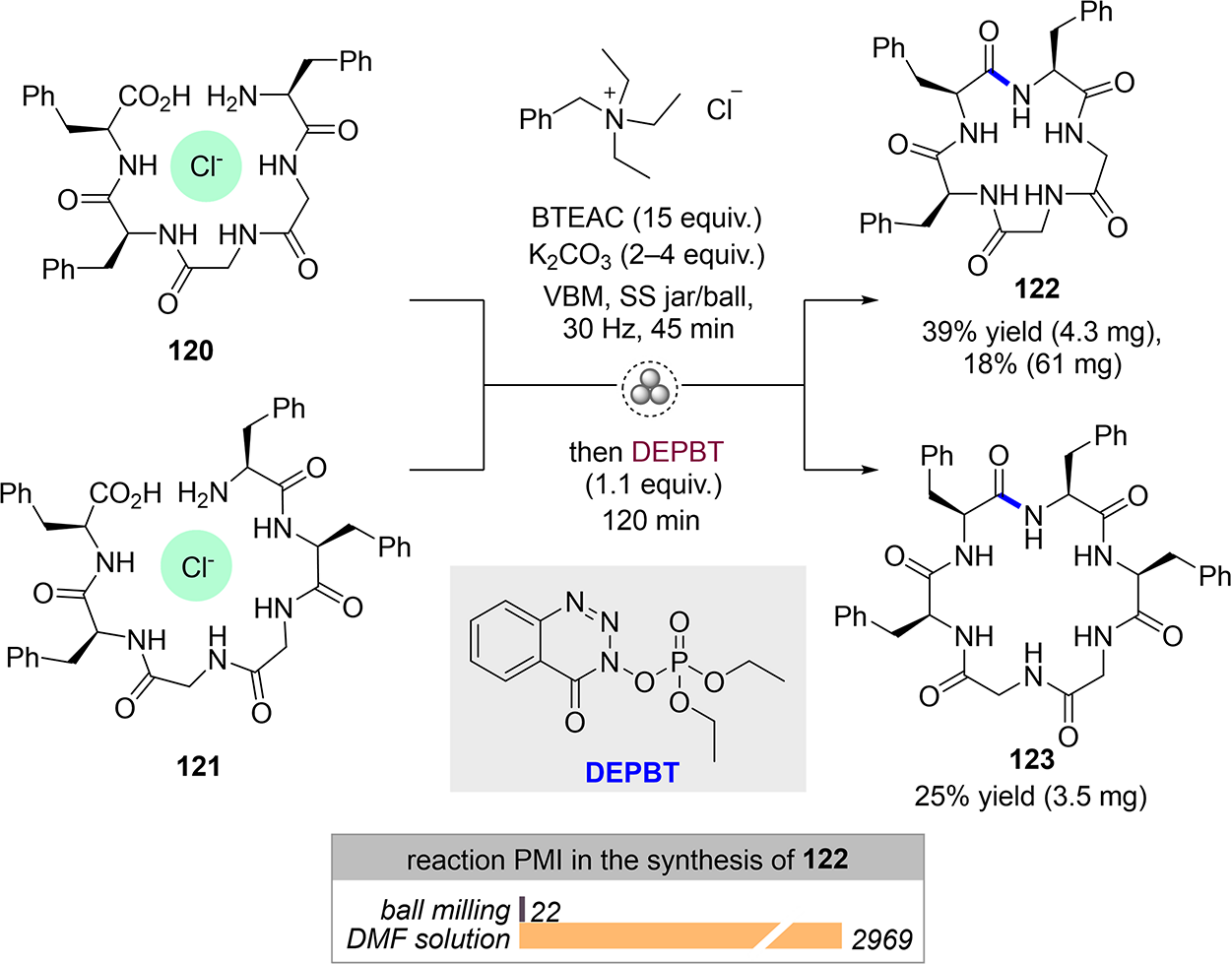
Chloride-Assisted Templated Mechanochemical Macrocyclization of Penta- and Hexapeptides

Two linear peptide precursors, NH_2_–Phe-Phe-Gly-Gly-Phe-OH (**120**) and NH_2_–Phe-Phe-Gly-Gly-Phe-Phe-OH (**121**), were cyclized using DEPBT as the coupling reagent, potassium carbonate as a base, and benzyltriethylammonium chloride (BTEAC) as a source of chloride anions. The templating effect of the chloride ion promoted a folded, quasi-cyclic conformation of the linear peptide, facilitating intramolecular cyclization. Tetraalkylammonium chlorides such as BTEAC were more effective templates than inorganic salts like KCl, NaCl, or CaCl_2_, whose higher lattice energies hampered participation of chloride ions in complexation. The mechanochemical method afforded lower yields of cyclic peptides **122** and **123** compared to synthesis in DMF solution (18% and 25% vs 35% and 58%, respectively). However, it offered several practical advantages, including a less laborious isolation protocol, avoidance of toxic solvents like DMF, and a dramatic reduction in reaction time from several days to minutes. In terms of waste-intensity, the mechanochemical process also demonstrated a significant improvement, reducing the reaction PMI value to 22 compared to the solution-based method (PMI = 2969).

#### 2.3.3 Miscellaneous Reagents for Single-Step Amide Coupling

In 2022, Casti et al.^115^ reported the mechanochemical synthesis of formamides and acetamides using imidazole or p-tosylimidazole (*p*-Ts-Im) as reagents (Scheme 37). Imidazole was found to be an effective promoter for the *N*-formylation of primarily aromatic amines, yielding the corresponding formamides in 52–90% yields. It was proposed that imidazole served a dual role in this transformation, both as a reactive promoter and as a solid grinding auxiliary.

**Scheme 37:**
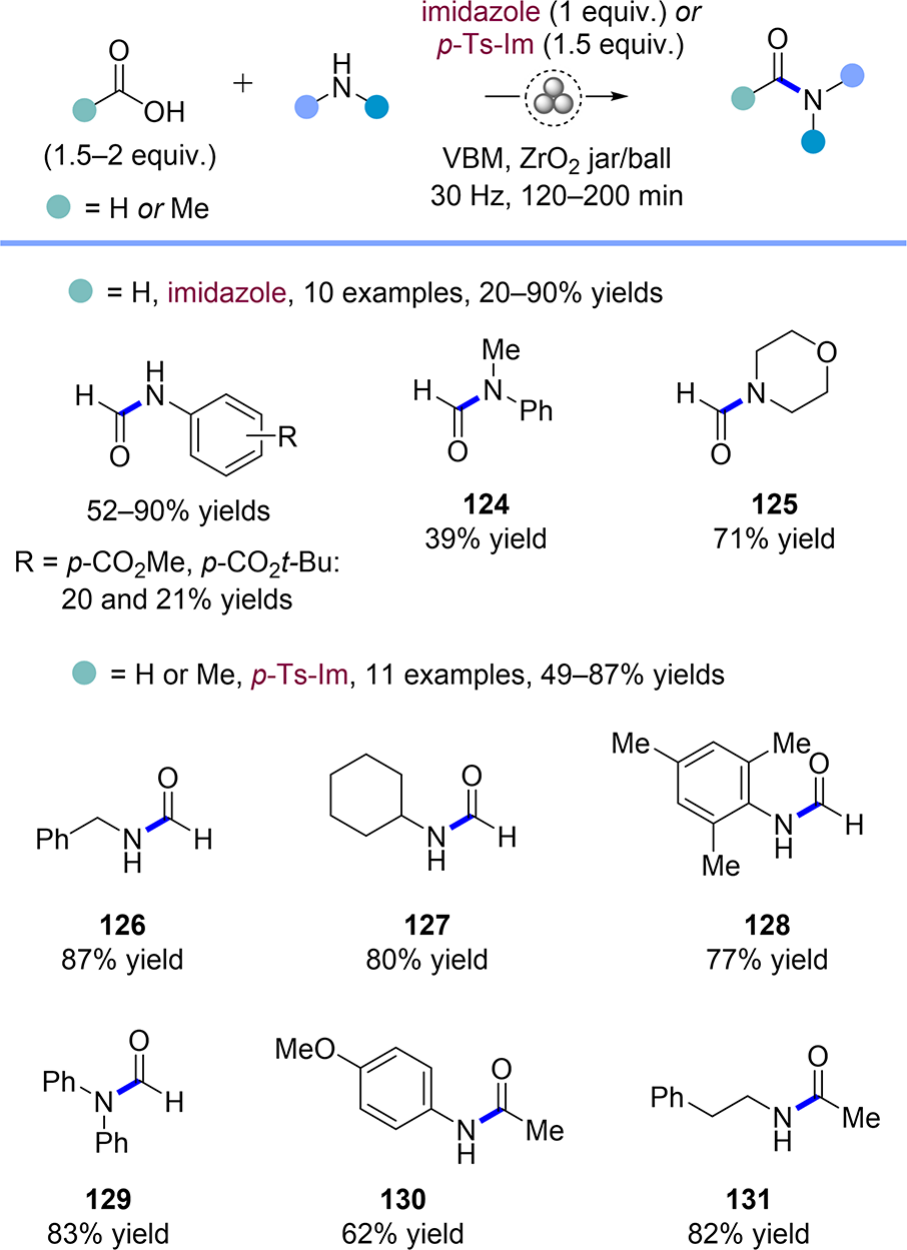
Synthesis of Formamides and Acetamides, Selected Examples

However, the method showed limitations: anilines bearing electron-withdrawing substituents such as *p*-alkoxycarbonyl gave poor yields (as low as 20%), and secondary amines like *N*-methylaniline afforded the product **124** in a reduced 39% yield. In contrast, *p*-Ts-Im proved to be a more effective activating agent, enabling the formylation and acylation of less reactive amines. For example, sterically hindered aniline derivatives and weakly nucleophilic diphenylamine were successfully converted into formamides **128** and **129** in 77% and 83% yields, respectively. The methodology was also extended to the acylation of both aromatic and aliphatic primary amines (e.g., for the synthesis of **127**, **130** and **131**). A noteworthy, though isolated, example of using inorganic compounds to promote amide coupling was reported by Zheng et al. (Scheme 38).^116^ The authors developed two reagent systems for ester synthesis under mechanochemical conditions. In the first system (A), a combination of iodine and potassium hypophosphite (KH_2_PO_2_) served as an organophosphorus-free alternative to traditional reagents such as triphenylphosphine. The second system (B) employed triethyl phosphite in combination with potassium iodide. In addition to enabling esterification, both systems mediated the amide coupling of benzoic acid with aniline.

**Scheme 38:**
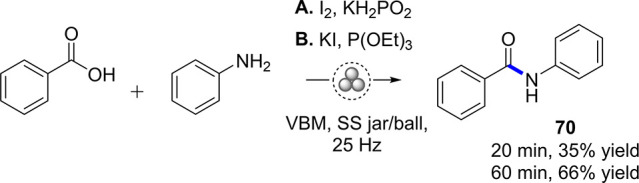
Amide Coupling of Benzoic Acid and Aniline Mediated by Triethyl Phosphite and Potassium Hypophosphite

### 2.4 Carboxylic Acids as Amide Precursors: Comparison of the Methods

In 2023, Lavayssiere and Lamaty^51^ reported the use of reactive extrusion for the synthesis of moclobemide **2** and teriflunomide **8**. The latter was obtained via hydrolytic ring opening of an isoxazole precursor **132**, which was first prepared through a mechanochemical coupling step (Scheme 39).

**Scheme 39:**
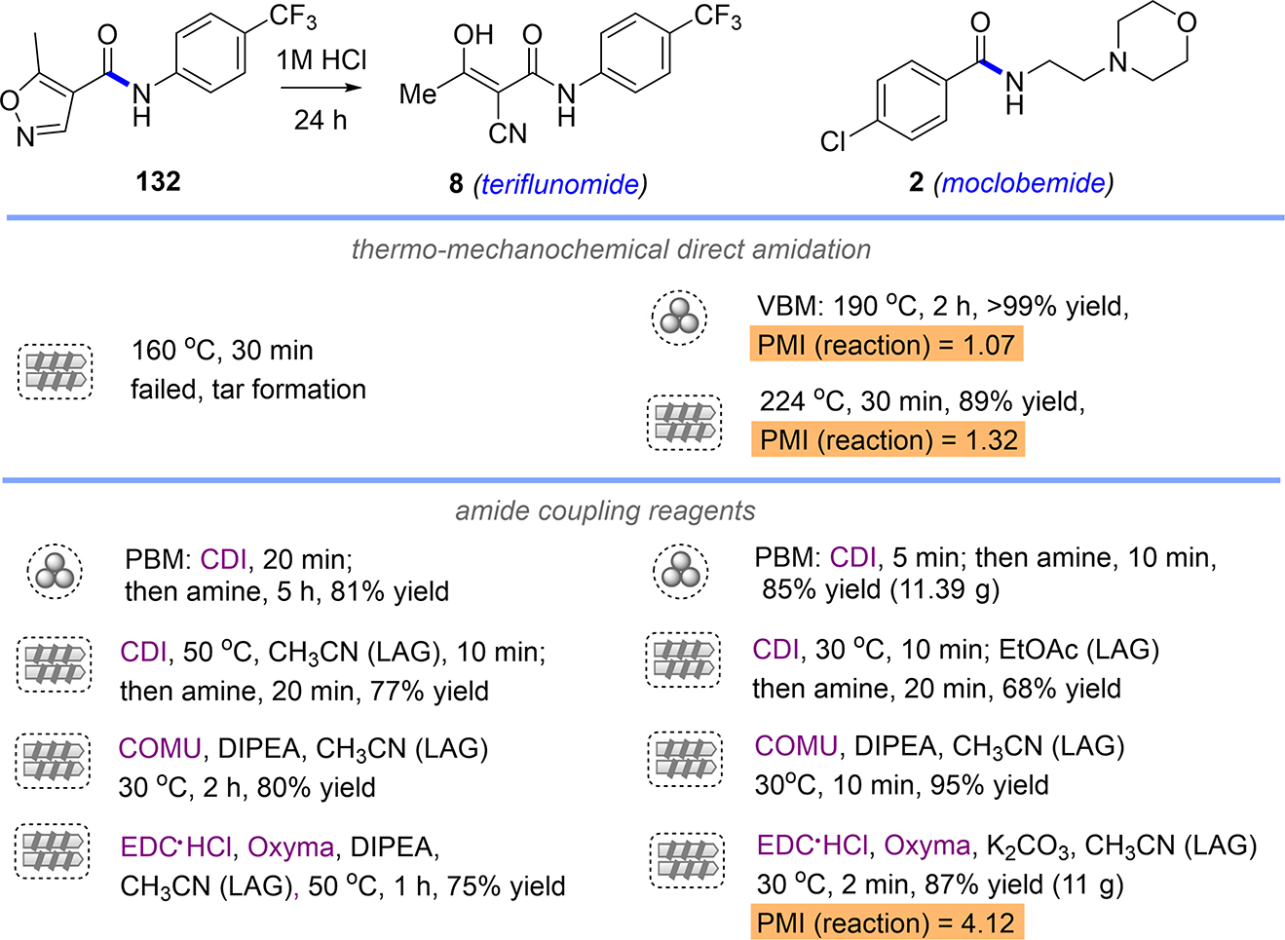
Mechanochemical Synthesis of Teriflunomide **8** and Moclobemide **2**. Comparison of Different Methodologies

During the optimization studies, various amide coupling reagents were evaluated in 0.5–1 g scale experiments. As both compounds have been prepared similarly using a range of mechanochemical techniques,^80,82,106^ including thermally promoted direct amide coupling,^49,50^ the data collected in this and other studies enable a meaningful comparison of these methods in terms of efficiency and suitability for scaled-up production of these pharmaceutically important targets.

Given that precursor compound **132** was previously prepared using CDI in a planetary mill,^80^ comparable yields of **8** (75–80%) were achieved in the TSE setup using CDI, COMU, and EDC as coupling reagents for the synthesis of **132**. Notably, the reaction time in TSE was shorter (30 min to 2 h), although elevated temperatures (up to 50 °C) were required. The EDC-mediated coupling proceeded more efficiently in the presence of Oxyma, but required recrystallization to remove byproducts. In contrast, thermally induced direct amide coupling failed, resulting in tar formation within the extruder, likely due to thermal decomposition of 5-methylisoxazole-4-carboxylic acid precursor.^50^

In the synthesis of **2**, previously prepared by thermally promoted direct amidation (in both TSE and VBM)^49,50^ and using CDI in a planetary mill on a scale of up to 11.39 g,^82^ the highest yields were obtained using COMU (95%) and EDC (87%), while CDI gave a lower yield of 68%. Notably, the combination of EDC with Oxyma and K_2_CO_3_ significantly accelerated the reaction, achieving complete conversion within 1 min. This outcome was successfully reproduced at scale, yielding 11 g of moclobemide in just 2 min. The pure product was isolated through extraction followed by recrystallization. This highlights TSE as a promising method for scale-up and positions the EDC/Oxyma system as a highly effective coupling strategy for amide bond formation at ambient temperatures (30 °C), albeit with the trade-off of a nearly 3-fold increase in the reaction PMI compared to thermally promoted direct amide coupling.

The comparative evaluation of coupling reagents and mechanochemical techniques for the synthesis of APIs such as teriflunomide and moclobemide underscores the diversity and complementary nature of available methodologies. The different methodologies offer distinct advantages and limitations depending on the specific context. The selection of an appropriate approach is influenced not only by the intrinsic suitability of the coupling reagents to promote amide coupling in a specific amine/carboxylic acid pair, but also by factors such as operational simplicity, compatibility with scale-up, and green chemistry considerations.

Building on these insights, we now turn to a broader comparative analysis of available methods for mechanochemical amide bond formation. Table 2 summarizes the frequency of application of various coupling reagents and activated carboxylic acid derivatives in mechanochemical amide synthesis. Among them, EDC holds prominence as the most frequently applied coupling reagent, especially for peptide synthesis, closely followed by activation with CDI, which is mainly used in the synthesis of nonpeptidic amides. Other approaches are comparatively less frequently employed (e.g., use of anhydrides or acid chlorides) or remain less developed with only a seminal publication reported, or serve a supplementary function to compensate for specific drawbacks of the dominant systems.

**Table 2:** Mechanochemical Amide and Peptide Synthesis: Number of Reported Examples by Activation Strategy^a^

Approach or coupling reagent	Number of publications	Estimated number of reported examples, including peptides^b^
EDC·HCl	15​	172 (95)
CDI	14​	187 (3)
Anhydrides, acid chlorides	12​	60 (0)
UNCA and NCA	5​	47 (47)
NHS esters	6​	47 (19)
TCT	1​	30 (11)
*N*-Acyl benzotriazoles	1​	29 (25)
*N*-Acyl saccharins	1​	50 (0)
DIC	1​	27 (27)
TFEDMA	1​	25 (6)
TBTU	1​	23 (23)
Imidazole or p-Ts-Im	1​	21 (0)
TCFH	1​	10 (0)
COMU	2​	9 (2)
Thermally driven amidation^c^	2​	9 (0)
HATU	4​^d^	3 (2)
DEPBT	1​	2 (2)

a Based on publications available before September 2025.

b Refer to the number of successful preparations, excluding optimization studies unless noted, and hydantoins. Number of prepared peptides is given in parentheses. Repeated syntheses of the same compound in different reports are counted separately.

c Using unactivated carboxylic acids.

d The reagent was used in optimization studies only.

The following overview outlines the current methodological landscape, providing a concise evaluation of the scope, advantages, and limitations of key approaches to support informed method selection for both small- and large-scale applications. While the defining features of the most prominent and representative methods are discussed below, a comparative summary is presented in Tables 3–5. These tables compile essential information on mechanochemical amidation from carboxylic acids, including the ranges of yields reported in key publications, the mechanochemical techniques and their main operational parameters, scalability, substrate scope and limitations, workup and purification strategies, green chemistry aspects, and other relevant details. In particular, Table 3 summarizes methods based on preactivation of carboxylic acids, including thermally driven direct amidation. For completeness, the *t*-BuOK-mediated amidation of carboxylic esters is also listed.^118^ Although this approach formally uses unactivated esters and is described later in Section 3.1.1, it follows the same retrosynthetic disconnection and is therefore included. Table 4 summarizes the use of amide coupling reagents, covering both stepwise and *in situ* activation strategies. Table 5 compares the atom economy of the various methods, calculated for the synthesis of moclobemide (**2**) from the corresponding carboxylic acid or derivative and the amine, excluding stoichiometric base and other additives. The safety aspects of the coupling reagents and methods are summarized in Table 6.

**Table 3:** Comparative Summary of Mechanochemical Amidation Methods Using Preactivation of Carboxylic Acids

Method	Technique and key parameters	Additives, LAG (η, μL·mg^–1^)	Scale	Time (min)	Yield (%)^a^	Workup and purification	Green chemistry aspects	Substrate scope, advantages	Limitations	Ref
Thermal	VBM, SS jar/ball, 30 Hz, 190 °C *or* TSE, 200 rpm, 164–252 °C	–	∼1–2 g	120–180 (VBM), 19–96 (TSE)	83–94	Collected pure or extracted. Purified by CC or recrystallization	Solvent free, high AE and low reaction PMI ≈ 1	Narrow, limited to thermally stable compounds	Necessitates high temperatures, incompatible with thermally unstable and volatile substrates or products	^49,50^
α-UNCA	VBM, SS jar/ball, 30 Hz	NaHCO_3_ (base)	∼0.5 g	60–300	70–100	Aqueous workup and extraction (EtOAc)	Solvent-free	Synthesis of α,α-dipeptides and tripeptides with excellent stereopreservation	Low yields due to low reactivity of specific α-UNCA derivatives or inefficient recovery from the reaction vessel	^59^
β-UNCA	VBM (Nylamid, 3800 rpm), SS ball	–	0.1 mmol	120	76–99	Aqueous workup and extraction (EtOAc)	Solvent-free	Synthesis of α,β- and β,β-dipeptides	β-UNCAs are less reactive than α-UNCAs	^63^
NCA	VBM, ZrO_2_ jar/ball, 30 Hz	GVL (2)^b^	0.2 mmol	30–180	75–99^c^	Collected by centrifugation (EtOH) followed by acidic treatment	Green solvent as a LAG additive, no *N*-protection needed	α-AAs, chemoselective *N*-acylation for AAs with unprotected side chains (Gln, Tyr)	High purity of NCA is critical to minimize polymerization byproducts	^64^
UNCA/NHS	VBM, SS jar/ball, 30 Hz	EtOAc, *t*-BuOAc (1.1–1.4)	∼1 g	5–60	61–99	Aqueous workup and extraction (EtOAc)	High Ecoscale score (84), green solvent as a LAG additive	Protected AAs (incl. hindered) for peptides with up to five AA residues. High milling load	Some substrates require longer milling time. Lower η values result in lower yields.	^60^
NHS esters	VBM, SS jar/ball, 28 Hz	EtOAc (0.25)	0.1–0.3 g	10–120	65–94	Aqueous workup and extraction (EtOAc), purified by CC	Green solvent as a LAG additive	Various amines and AAs, chemoselectivity (*N*- over *O*-acylation)	NHS-esters are inclined to hydrolysis (promoted by strong base and high milling frequency)	^65^
HOSu ester/UNCA	TSE, 50–150 rpm, 40 °C	Acetone (0.15)	∼10 g	1.5–10	81–92	Aqueous workup and extraction (EtOAc)	High STY (>100x vs batch)	Protected AAs	Validated for di- and tripeptides only, LAG is essential	^62^
RCOCl or (RCO_2_)_2_	VBM, TSE, manual grinding	No additives, or silica, K_2_CO_3_ (for RCOCl)	∼1 mmol to kg/day	5–120	23–99	Aqueous workup and extraction (EtOAc); Addition of base and filtration; Collection in MeOH and filtration	Solvent-free, continuous	Wide range of amines, key for crystalline 2D aromatic polyamides	Synthesis of PDI is less effective with sterically hindered anilines	^69–70,74^
*N*-Acyl benzotriazoles	VBM, SS jar/ball, 30 Hz	EtOAc (15 μL)	∼1 mmol	90–180	20–98	Precipitation in water	PMI(process): 304–536, RME: 34–49%	Protected AAs, synthesis of di- and tripeptides	Lower yields for longer peptides, risk of Fmoc deprotection	^75^
*N*-Acyl saccharins	VBM, ZrO_2_ jar/ball, 30 Hz	–	∼1 mmol	30–180	75–98	Treatment with EtOAc and filtration	Solvent free, safe and recyclable activator, PMI (process): 27.9	Wide range of amines: *N*-formyl, *N*-acetyl, and *N*-propionyl derivatives	Poorly nucleophilic amines require longer milling	^77^
Me or Et esters	VBM, SS jar/ball, 30 Hz *or* TSE, 25–200 rpm, 50 °C	*t*-BuOK	0.1–3 g (VBM), up to 500 g (TSE)	60–120 (VBM)	11–98	Aqueous workup and extraction, optional CC	PMI(reaction): < 2, good AE	Broad, includes various aliphatic and aromatic amines and carboxylic esters, lactones. Applicable to API synthesis	Sterically hindered and poorly nucleophilic amines, sterically hindered mesitoic esters. Competing processes promoted by a strong base, including complete racemization of base-sensitive stereocenters	^118,120^

a Yields of isolated products unless stated otherwise.

b Amount of GVL per mg of NCA.

c Yield determined by HPLC.

**Table 4:** Comparative Summary of the Preparative Use of Amide Coupling Reagents under Mechanochemical Conditions

Coupling reagent	Technique and key parameters	Additives	Scale	Time (min)	Yield (%)^a^	Workup and purification	Green chemistry aspects	Substrate scope, advantages	Limitations	Ref.
CDI^b^	PBM, 500 rpm	–	1–10 g	15–360	14–96	Addition of water and filtration	Solvent-free	Broad, especially in the synthesis of nonpeptidic amides, hydroxamic acids, ureas, carbomates, hydantoins, applicable to API synthesis, no significant epimerization	Sterically hindered amines, electron-deficient amines require extended milling times	^79–312^
TCT^b^	Manual grinding	K_2_CO_3_ (base), DCM (LAG), PPh_3_ (10 mol %)	1 mmol	30–40	51–95	Extraction, CC	–	Protected AAs and aliphatic amines, aromatic and aliphatic carboxylic acids	Less effective for weakly nucleophilic amines; requires inert atmosphere, laborious purification.	^85^
TFEDMA^b^	VBM, SS jar/ball, 30 Hz	–	∼1 g	20–40	71–99	CC, or recrystallization	PMI(reaction): 3.6	Wide range of acids and amines (incl. secondary and electron-poor), no significant epimerization	Requires CC for purification, one-pot protocol delivers reduced yields	^86^
TBTU^b^	PM, SS jar/ball, 500 rpm	Cs_2_CO_3_ (base)	1 mmol	10–150	35–99	Extraction, CC or recrystallization	–	Narrow, currently limited to protected AAs	Significant epimerization, likely promoted by strong base	^87^
EDC·HCl	VBM, 30 Hz *or* Single screw extruder, 20–300 rpm, 27 °C	DMAP or NaH_2_PO_4_ (base),^c^ Oxyma or HOBt,^d^ CH_3_NO_2_ or EtOAc (LAG)^e^	0.1–2 g (VBM) up to 100 g (extr.)	10–180 (VBM) 1–2 (extr.)	39–99	Addition of water and filtration	PMI(reaction): ∼4	Broad, including synthesis of peptides. Sterically hindered AAs, like Aib. Tolerates unprotected OH, validated in the synthesis of APIs, the lowest recorded epimerization level (with Oxyma)	Poorly nucleophilic amines, poly(carboxylic acid)s, sterically hindered carboxylic acids may deliver low yields	^92–106^
DIC	TSE, 200 rpm, 40 °C	DIEA (base), Oxyma,^d^ EtOAc (LAG)	2–56 g	4–24	75–99	Extraction, CC or precipitation and filtration	Very high STY	Narrow, currently limited to the synthesis of di- and tripeptides, no significant epimerization	Plausible DKP formation above 60 °C	^107^
COMU	VBM, ZrO_2_ jars/balls, 30 Hz	K_2_HPO_4_ (base), EtOAc (LAG)	0.4 mmol	20	86–96	Addition of water and filtration, or extraction and CC	PMI(reaction): 4	Includes poorly nucleophilic amines, suitable for peptide coupling, low epimerization levels	Guanidine byproducts, may require CC. Low AE	^110,111^
TCFH	VBM, ZrO_2_ jars/balls, 30 Hz	K_2_HPO_4_, NMI (bases), EtOAc or CPME (LAG)	0.4 mmol	20–90	70–96	Addition of water and filtration, or extraction and CC	PMI(reaction): 3.7	Includes poorly nucleophilic amines, efficient for sterically hindered and poly(carboxylic acid)s	Guanidine byproducts, may require CC, notable epimerization for 2-arylpropionic acids	^110,111^
DEPBT	VBM, 30 Hz, *or* PM, 650 rpm, SS jar/balls	BTEAC (template), K_2_CO_3_ (base), DMF (LAG)^f^	3.5–61 mg	120	18–25	CC	Avoids high dilution in DMF	Narrow, templated macrocyclization of penta- and hexapeptides	Low preparative yields	^114^
Imidazole, *p*-Ts-Im	VBM, ZrO_2_ jars/balls, 30 Hz	–	1 mmol	120–200	50–92	Extraction, optional CC	–	*N*-formylation and *N*-acylation of amines, including weakly nucleophilic and sterically hindered amines (with *p*-Ts-Im)	Electron-poor and *N*-methyl anilines (with imidazole)	^115^

a Yields of isolated products.

b The reagent used in two-step manner (activation, followed by reaction with amine).

c Addition of base is optional and case-depended (e.g., required for the reactions of amine salts). Other bases, besides the mentioned, can be used (Mg–Al hydrotalcite, hydroxyapatite).

d Epimerization suppressor.

e Other LAG additives, besides the mentioned, can be used.

f LAG additive is optional.

**Table 5:** Comparison of Atom Economy for Different Methods^a^

Starting material, method	AE (%)
RCO_2_H, direct condensation	94
RCO_2_Et, amidation of ester	85
RCOCl (RCO_2_H + phosgene)	70
RCOCl (RCO_2_H + SOCl_2_)	66
RCOCl (RCO_2_H + oxalyl chloride)	65
RCO_2_H, DIC	65
RCO_2_H, TFEDMA	62
RCO_2_H, CDI	60
RCO_2_H, TCT	57
RCO_2_H, EDC·HCl	56
RCO_2_H, TCFH	47
RCO_2_H, TBTU	44
RCO_2_H, HATU	40
RCO_2_H, COMU	38

a Calculated for the synthesis of moclobemide (**2**) from *p*-chlorobenzoic acid (R = *p*-ClC_6_H_4_) or its respective derivative and the corresponding amine, excluding stoichiometric base and other additives.

**Table 6:**
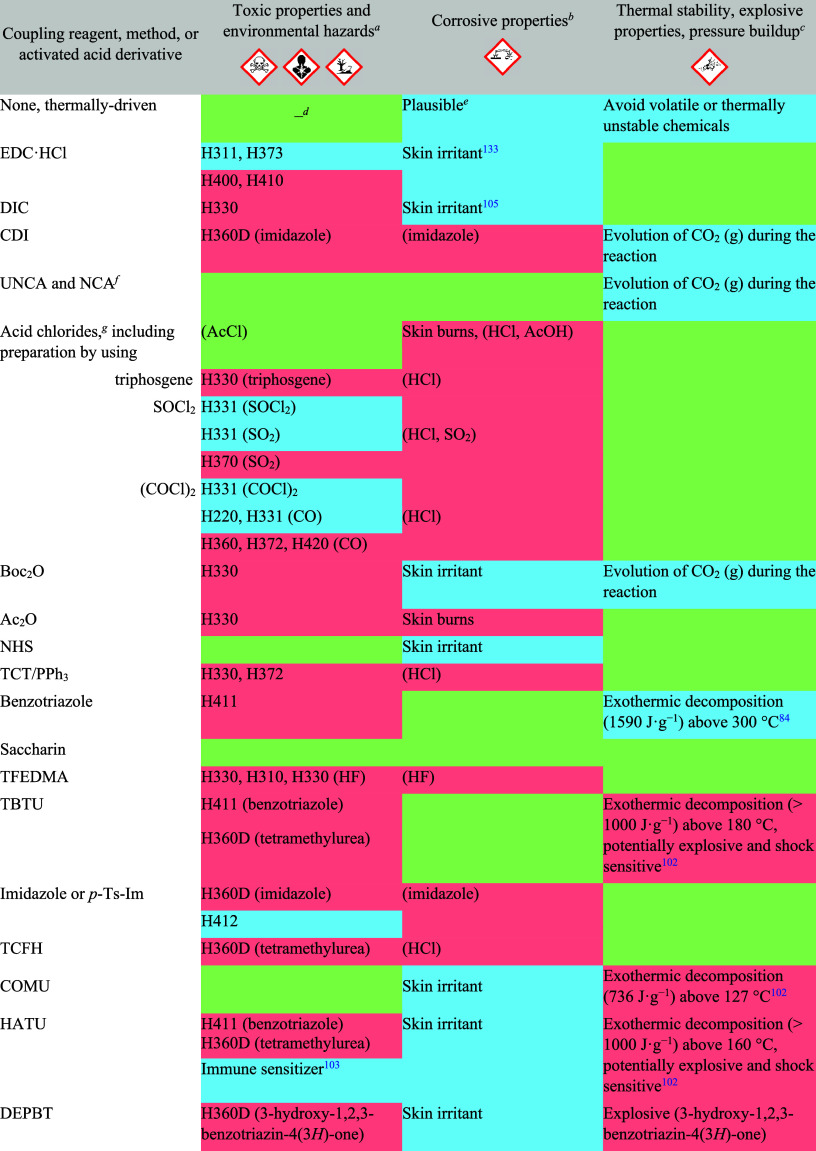
Health, Environmental and Safety Hazards of Amide Coupling Reagents and Activation Methods

a Hazard statements according to SDSs (https://www.sigmaaldrich.com) or ECHA database (https://echa.europa.eu/)^122^ for reagents used in amide coupling or in the preparation of activated carboxylic acid derivatives, and their respective reaction byproducts (indicated in parentheses). Safety categories (red, blue, and green) are assigned according to the CHEM21 ^43^ green chemistry toolkit criteria: red – high concern; blue – problematic; green – no significant known hazards. Physical hazards: H220 – extremely flammable gas. Health hazards: H300 – fatal if swallowed, H310 – fatal in contact with skin, H311 – toxic in contact with skin, H330 – fatal if inhaled, H331 – toxic if inhaled, H360 – may damage fertility or the unborn child, H360D – may damage the unborn child, H370 – causes damage to organs, H372 – causes damage to organs through prolonged or repeated exposure, H373 – may cause damage to organs through prolonged or repeated exposure. Environmental hazards: H400 – very toxic to aquatic life, H410 – very toxic to aquatic life with long lasting effects, H411 – toxic to aquatic life with long lasting effects, H412 – harmful to aquatic life with long lasting effects, H420 – harms public health and the environment by destroying ozone in the upper atmosphere.

b Corrosive properties of the reagents or their respective byproducts (indicated in parentheses) according to SDSs (https://www.sigmaaldrich.com) or ECHA database (https://echa.europa.eu/). Color safety codes: red – corrosive properties present, causes skin burns; blue – plausible corrosive properties or skin irritant; green – no corrosive properties known.

c Thermal stability data according to refs 76, 88, and 123 and available safety data (https://echa.europa.eu/). Color safety codes: red – exhibit high thermal decomposition energy or potentially explosive; blue – use with caution; green – most preferred, no known hazards.

d Hazards arising from the starting materials and/or the amide product should be considered.

e Starting materials (acid and/or amine) can exhibit corrosive properties.

f Excluding preparation by using triphosgene.

g Based on properties of acetyl chloride as a representative reagent.

These considerations include, first, hazards to human health and the environment, such as acute or environmental toxicity.^121^ Second, corrosive properties are noted, as these may pose risks of skin burns or irritation, and may also contribute to wear of mechanochemical equipment (for example, stainless steel vessels), potentially leading to metal leaching and product contamination. Third, information on thermal stability, potential explosiveness (shock sensitivity), and the risk of pressure build-up in a closed reaction vessel is presented.

EDC-mediated amide coupling has emerged as the most frequently employed method in mechanochemical amide and peptide synthesis. This prominence can be attributed to its practical advantages and well-documented performance across both peptidic and nonpeptidic systems. EDC has been proven effective with a wide range of substrates, including sterically hindered amino acids such as α-aminoisobutyric acid (Aib),^93^ as well as a broad variety of primary and secondary amines and carboxylic acids, showing reduced efficiency only in challenging cases involving poorly nucleophilic amines and sterically hindered carboxylic acids.^110^

In terms of functional group tolerance, EDC is notably more chemoselective than uronium-type reagents, often enabling amide bond formation in the presence of unprotected hydroxyl groups without undesired side reactions.^102^ When combined with additives such as Oxyma, EDC-mediated peptide couplings exhibit particularly low levels of epimerization,^97^ outperforming other common coupling systems in this regard. Notably, this coupling system was used in the sequential formation of several peptide bonds in a tetrapeptide.^94^

From a practical perspective, EDC-based couplings typically proceed under ambient conditions without requiring the addition of an external base and in a single step, exhibit fast reaction kinetics, and involve a simple workup through aqueous washing, as the urea byproducts are water-soluble. Its scalability has been demonstrated in twin-screw extrusion (TSE) setups,^51,106^ enabling multigram synthesis with high efficiency. Couplings mediated by DIC and other carbodiimide-type reagents can deliver comparable performance^92^ but remain significantly less explored in mechanochemical applications to date.^107^

Activation with CDI, which proceeds via the formation of acyl imidazolium intermediates, offers a robust and versatile alternative to EDC, particularly in the synthesis of nonpeptidic amides. This two-step protocol is characterized by a broad substrate scope and straightforward purification procedures and has been shown to deliver high yields even on multigram scales. It is suitable for amide coupling with poorly nucleophilic, electron-deficient anilines, although such reactions typically require extended milling times.^80^ However, its efficiency tends to decrease when applied to sterically hindered substrates, such as secondary or tertiary aliphatic amines. In addition to conventional amide bond formation, CDI has also been successfully employed in the mechanochemical synthesis of hydroxamic acids.^84^ Despite its utility and low cost, CDI is used only rarely for peptide synthesis under mechanochemical conditions, with only a few reported examples.

Carboxylic acid anhydrides and acid chlorides, though classical and historically first-used acylating agents,^301,72^ have found only moderate application in mechanochemical amide synthesis. Their relatively frequent use is primarily linked to their commercial availability or ease of preparation, rather than broad methodological development. In comparison to EDC or CDI, their application is approximately three times less prevalent. Nonetheless, they serve as practical and efficient reagents in specific contexts, particularly where the acylating agents are readily available. These include amino group protection strategies,^67,68^ the synthesis of pharmaceutically relevant targets such as procainamide and paracetamol on gram scales,^69,307^ and the preparation of functional polyamides and polyimides for materials science applications.^70–74^ Several of these transformations have also been adapted to reactive extrusion, further demonstrating their utility in scaled-up mechanochemical processes.^74^

Historically, the earliest mechanochemical methods based on NCAs and NHS esters demonstrated high efficiency in peptide synthesis,^59,60^ including longer peptide chains containing up to five amino acid residues. These approaches proved especially suitable for couplings involving sterically demanding natural amino acids and have been successfully scaled up using twin-screw extrusion technology. *N*-Acyl benzotriazoles have also been employed for the synthesis of di- and tripeptides, achieving yields ranging from 20–98%.^75^ These reagents have shown particular utility in biomolecule functionalization but tend to deliver lower yields with sterically hindered substrates. *N*-Acyl saccharin derivatives can be regarded as safe-to-handle and recyclable acyl transfer reagents suitable for the synthesis of formamides, acetamides, and propionamides.^77^ However, broader applicability of all these methods is limited by the need to presynthesize the activated intermediates in a separate step, making them less competitive today compared to more streamlined one-step protocols such as EDC-mediated coupling.

Other coupling methodologies are developed to overcome specific limitations of established systems. Uronium-type reagents, such as COMU and TCFH, were introduced to tackle “difficult” amide couplings involving poorly nucleophilic amines and hindered carboxylic acids, as well as to enable efficient polyamidation of multifunctional substrates.^110^ However, their use with more nucleophilic amines can lead to undesired guanidine byproducts, and high molecular weight results in low AE values (Table 5). TFEDMA-mediated activation presents an alternative that combines high yields with improved atom economy compared to the uronium coupling reagents. It is also effective for less reactive amines and does not require the use of bases, although it operates more efficiently via a two-step protocol.^86^

Some coupling reagents have been applied in more niche contexts. For example, DEPBT has been used to achieve template-assisted peptide macrocyclization,^114^ while imidazole and *p*-Ts-Im have been employed for efficient formylation and acylation reactions.^115^ Other methods offer synthetic flexibility but present limitations that hinder their broader adoption. TBTU-mediated peptide coupling, while efficient in terms of yield, suffers from significant levels of epimerization.^87^ The TCT-mediated coupling method is applicable to both peptides and nonpeptidic amides, but requires manual grinding under an inert atmosphere, involves a laborious workup and isolation procedure.^85^

Lastly, thermally promoted direct amide coupling represents a highly attractive approach due to its highest AE (producing only water as a byproduct) and nearly “ideal” reaction PMI ≈ 1.^49^ This method has also been adapted to TSE-based scale-up.^50^ However, it remains underexplored (only a few reported examples to date) and is limited to substrates and amide products that are thermally stable and nonvolatile.

Although carboxylic acids represent abundant and versatile feedstocks for amide synthesis, and the methods discussed above offer considerable scope and practicality, none are without limitations. Challenges such as the need for activating agents and scope limitations continue to motivate the search for alternative approaches. These constraints have spurred the development and mechanochemical adaptation of nonclassical strategies that rely on alternative starting materials and distinct activation modes. These methodologies are detailed in the section 3.

### 2.5 Practical Guidelines and Troubleshooting

#### 2.5.1 Ball Milling

Defining a comprehensive practical guideline for mechanochemical amide coupling is challenging, because reaction performance depends on numerous variables, including the chemical nature and physical state of the substrates, reaction type and scale, and the intended application of the product. A full treatment of these aspects is beyond the scope of the present article, and dedicated publications offering practical guidance can be recommended for further reading.^27^ Here, we outline several general recommendations that may assist researchers in initiating and optimizing new amidation systems under mechanochemical conditions in a ball mill.

A typical starting point is the coupling of equimolar amounts of a solid carboxylic acid and an amine (or its hydrochloride salt in the presence of a base) at room temperature, on a ∼ 100 mg scale in a vibratory ball mill. Ideally, the reaction affords the amide product as a solid, sparingly soluble in water, requiring only aqueous washing and filtration for isolation. The choice of coupling reagent is critical. EDC·HCl is often the most general and reliable option, offering broad substrate scope, good functional group tolerance, and a low risk of epimerization, particularly when used with additives such as Oxyma. If EDC proves ineffective, alternative reagents such as CDI, COMU, or TCFH may be explored. COMU and TCFH are especially useful for less nucleophilic amines, sterically demanding carboxylic acids, and polycarboxylic substrates, with the TCFH/NMI combination being particularly reactive.

The choice of LAG additive should also be considered, as it can greatly influence reaction efficiency and the rheological behavior^124^ of the reaction mixture. Appropriate LAG conditions can improve mixing and prevent issues such as “snow ball” effect^125^ or ball immobilization due to formation of sticky pastes. Alternatively, solid grinding additives (e.g., NaCl, Na_2_SO_4_) may be used. Although the selection of solvent and the η value remains empirical and must be optimized for each system, polar aprotic solvents have generally proven effective in mechanochemical amide coupling. When selecting a LAG additive, it is advisible to prioritize greener solvent options (e.g., acetate esters, GVL, CPME, DMI).

Process selection could also be guided by the principles of green chemistry. In this case, health and environmental hazards associated with coupling agents and their byproducts (Table 6), the difficulty of removing residual reagents and byproducts, scalability and cost, and atom economy (Table 5) are all important factors to consider. The use of excess reagents should be minimized whenever possible. For base selection, inexpensive and nontoxic inorganic salts such as phosphates or carbonates are preferable. These bases also improve carbon atom economy compared to organic amine bases.

Water quench followed by filtration is generally the most attractive workup strategy, offering operational simplicity and a low total PMI. However, its applicability depends on the solubility and physical properties of both the product and the byproducts. When this approach is not feasible, more waste-intensive and laborious procedures, such as liquid–liquid extraction followed by optional column chromatography or recrystallization, may be unavoidable.

If amide coupling fails with the established coupling reagents, or if their use is undesirable, alternative activation modes or precursors may provide a suitable option, or a different coupling reagent can be explored. When establishing a new coupling system, it is advisible to begin with a substrate pair previously shown to couple successfully using widely applied reagents such as EDC or CDI, and to avoid combinations complicated by pronounced steric hindrance (on the acid) or very low nucleophilicity (of the amine). Initial reactions may be performed under neat grinding conditions (η = 0), and, if the outcome is unsatisfactory, various LAG additives (typically polar solvents) or bases can be introduced. Attention should be paid to the rheological behavior of the mixture during milling, as changes can indicate the need for adjusting the amount of LAG additive or introducing a solid grinding agent. Reaction kinetics should be assessed, including evaluation of the effect of milling frequency (for example, 30 vs 10 Hz). Where possible, the mechanochemical process should be compared with a corresponding solution-phase benchmark, including green chemistry metrics such as total PMI, to substantiate any sustainability advantages.

Analysis of crude reaction mixtures can be carried out using readily available *ex-situ* techniques such as NMR or HPLC, provided that the reaction is quenched to prevent further conversion in solution. The use of an internal standard can improve the reliability of quantitative data, but should be approached with caution, as single-point sampling may lead to misleading results if the reaction mixture is not homogeneous.^126^

The substrate scope used to evaluate reaction performance may be concise, but it should be diverse and representative,^127^ demonstrating not only successful cases but also limitations, for example those arising from steric hindrance or low nucleophilicity of the amine. Peptide coupling should be examined, along with reactions involving both aromatic and aliphatic substrates, sterically demanding or poorly nucleophilic partners, and molecules bearing sensitive functional groups such as free hydroxyl groups. It is also useful to assess the influence of aggregate state (solid vs liquid substrates) on reactivity. Including a benchmark substrate widely explored by other methods, such as moclobemide (**2**) or another frequently synthesized amide, enables direct comparison. Epimerization levels should be monitored, particularly for sensitive stereocenters,^97^ such as those in phenylglycine or α-arylpropionic acids.

Selection of process parameters such as milling load (filling degree), milling frequency, number and mass of milling balls, jar dimensions, and vibration amplitude is important, as these factors collectively determine the mechanical energy transferred to the reactants.^128^ All such parameters should be reported transparently. For a typical 15 mL milling jar, an appropriate starting point is a low milling load (100–200 mg) with a single milling ball at 30 Hz, which is also a common operating maximum for many commercial vibratory ball mills. These conditions are frequently used for optimization and allow easier comparison across studies. Increasing the milling load typically leads to reduced yields. A drop in yield when the frequency is reduced below 30 Hz indicates that mechanical energy input is necessary to initiate and, plausibly, sustain the reaction. Conversely, if similar yields are obtained even at low frequencies, this may indicate that continuous mechanical agitation is not essential, which can be tested by manual mixing of the reactants in a vial. When reproducing literature results on a different instrument, matching the effective mechanical energy input^128^ can serve as a useful starting point. For optimization, scale-up, and fine adjustment of parameters, statistical tools such as Design of Experiments (DoE) or Bayesian Optimization (BO) are recommended instead of varying one factor at a time.

The choice of milling equipment and media is also important. Abrasion of the jar and balls is inherent to mechanochemical processing, and the extent of contamination depends on the chemical nature of the reactants, as well as milling intensity and duration. Stainless-steel jars may introduce trace metal impurities^129^ and should be avoided when highly corrosive reagents or byproducts are present (see Table 6). Zirconia jars and balls can undergo gradual wear, releasing fine ZrO_2_ particles, which then require removal by dissolving the product and filtering. Such issues must be evaluated on a case-by-case basis, with possible mitigation strategies including the use of more chemically resistant materials, avoiding excessive mechanical energy input, or applying additional purification steps. Acceptable impurity levels will depend on the intended application of the product, noting that metal impurities in APIs are strictly regulated.

#### 2.5.2 Reactive Extrusion: Operating Windows and Failure Modes

Reactive extrusion is a promising scale-up technology that has already demonstrated efficiency for continuous, multigram amide synthesis. Below, we outline the typical operating regimes used in selected case studies to add detail beyond the brief descriptions provided earlier. The key characteristics of these selected methods are summarized in Table 7. Furthermore, mitigation strategies for the most common failure modes are summarized in Table 8. These include thermally driven side reactions or degradation, hydrolysis or decomposition during prolonged residence times, and stalling, which is among the typical issues encountered at ambient temperature. Additional practical guidance lies beyond the scope of this article but can be found in the tutorial review cited.^34^

**Table 7:** Representative Operating Windows for Amide Synthesis in Screw Extruders

Platform	Method	Screw speed (rpm)	Residence time (min)^a^	Temperature set points and process aids	Throughput or scale	Key findings and limitations	Ref.
TSE (conical, 2 mL, SS)	NHS/UNCA peptide coupling	150	1.5–10	40 °C, acetone (η ≈ 0.15 mL·g^–1^)	∼10 g	High STY vs solution. Hydrolysis of activated ester at 100 °C. Extrusion fails without LAG (no reaction)	^62^
	Amide synthesis with CDI, EDC·HCl, COMU	200	1^b^	30–50 °C, EtOAc or MeCN (η ≈ 0.3–0.6 mL·g^–1^)	∼2 g	LAG is essential, poor flow without LAG prevents recirculation	^51^
	DIC/Oxyma peptide coupling	200	4–24^c^	40 °C, EtOAc	up to 56 g, 4.7 × 10^6^ kg·m^–3^·day^–1^	Low epimerization, DKP formation during in-extruder Boc deprotection attempts (60–80 °C)	^107^
TSE (corotating, SS)	Aminolysis of esters (with KO*t*-Bu)	200	420^d^	50 °C, Na_2_SO_4_ for liquid substrates	up to 0.5 kg, 70 g·h^–1^	Compaction/″Torque-out″ without GA. Hydrolysis of ester in the presence of hygroscopic GAs (LiCl)	^120^
TSE (conical, SS)	Thermally driven direct amide coupling	200	17–96	160–250 °C	∼1–2 g	Internal pressure retains volatile reactants. Decomposition and tar formation for thermally unstable substrates	^50^
Single-screw (PTFE)	Amide synthesis with EDC·HCl	100	0.5–2	27 °C	up to ∼100 g, 50 g·h^–1^	Solvent-free. Ambient temperature operation, short residence time	^106^

a In continuous mode unless stated otherwise.

b Up to 120 min in recirculation mode.

c Recirculation, continuous mode for scale-up.

d In an upscaled continuous run (0.5 kg scale), feed rate of starting materials 2.3 g·min^–1^.

**Table 8:** Failure Modes and Mitigation Strategies in Reactive Extrusion

Failure	Mitigation strategy	Example	Ref.
Thermal degradation	Use lower temperature, if possible, or switch to low-temperature synthetic method	Synthesis of teriflunomide fails at high temperature, forming tar in the extruder, but can be performed at lower temperature extrusion using amide coupling reagents	^51^
Thermally driven generation of byproducts	Use lower temperature, if possible, or switch to low-temperature synthetic method	DKP byproduct (13–30%) during attempted in-barrel Boc deprotection at 60–80 °C. No DKP formed when extrudate was poured into an aq. H_3_PO_4_ for Boc-deprotection. No DKP formation for peptide coupling at 40 °C	^107^
Thermally accelerated hydrolysis	Use lower temperature, if possible, or maintain anhydrous conditions	Peptide coupling at 100 °C caused partial hydrolysis of NHS ester. Lower temperature (40 °C) was used	^62^
Rheology-driven stoppages (compaction, stalling)	Introduce LA or GA to adjust the rheological properties	Viscous feeds can cause compaction and motor ″torque-out. Use of additives to adjust liquid/solid ratio solved the problem	^51,120^
Hydrolytic decomposition of starting materials	Replace hygroscopic additives, if any, maintain anhydrous conditions, or use less sensitive to hydrolysis starting materials	Hygroscopic GA (LiCl) resulted in recovery of more than half of starting materials, and ∼ 20% benzoic acid via ester hydrolysis. In contrast, NaCl and Na_2_SO_4_ did not exhibit ester hydrolysis	^120^
Decomposition from too long residence time	Tune feed rates and screw configuration to achieve the target residence time without overprocessing	Extended residence times under harsh conditions (strong base, *t*-BuOK) can erode yields in the amidation of esters	^120^

Across the selected reports, two regimes are evident: (i) mild-temperature processes (typically 25–50 °C) that use amide coupling reagents and achieve minute-scale residence times, provided the material is maintained as a flowable paste by adding liquid additives (LA) and solid grinding auxiliaries (GA);^51,62,106,107,120^ (ii) thermally promoted direct amidation (≥160 °C) that dispenses with coupling reagents, where temperature control, volatility, and pressure management become the primary process drivers.^50^ Interestingly, the screw-generated back-pressure can help retain amines near or above their boiling points, and evaporation of water at elevated temperatures contributed to shift the equilibrium toward amide formation.

From the case studies, a few practical recommendations relevant to amide synthesis can be formulated. First, the temperature regime should match substrate stability and volatility. Reagent-mediated TSE at moderate temperatures (around 40 °C) with minute-scale residence times is suitable for thermally labile compounds, including amino acids and peptides. However, the possible formation of diketopiperazine byproducts during peptide coupling should still be kept in mind, even at these temperatures. High-temperature direct amidation can be applied to more robust acid–amine pairs, particularly when the very low reaction PMI is a decisive advantage. Second, the reaction mixture should be engineered into a homogeneous extrudable paste. A small dose of a suitable solvent, supplemented when necessary, with a solid grinding auxiliary, can convert powders or powder–liquid blends into a cohesive, shearable paste suitable for extrusion.

## 3 Nonclassical Routes to Amides

This section provides an overview of nonclassical approaches^7^ to amide bond formation that do not rely on prior activation of carboxylic acids, or that generate amide products through alternative bond constructions. These methods employ different starting materials or reaction pathways that expand the synthetic toolbox for amide construction and therefore provide a complementary approach to the classical strategy.

The first group of methods (Section 3.1) involves the use of redox-neutral amine and carboxylic acid surrogates, in which the carbon and nitrogen atoms that form the amide bond retain their oxidation states throughout the reaction. Next, redox-based strategies are presented, including transition-metal-catalyzed C–H activation and other transformations involving organometallic intermediates (Section 3.2). Section 3.3 subsequently covers reductive C–N coupling, C–F bond activation, and the Leuckart reaction. Finally, Section 3.4 covers rearrangements and multicomponent reactions.

### 3.1 Use of Amine and Carboxylic Acid Surrogates

Recent advances in mechanochemical amide bond formation have demonstrated the utility of various surrogates as alternatives to traditional carboxylic acids and amines. These starting materials offer several advantages, such as greater stability, ready accessibility, and enhanced reactivity under solvent-free conditions. This section highlights key developments in the use of such surrogates, grouped into four categories: amination of unactivated esters and lactones (Section 3.1.1); transamidation of phthalimide (Section 3.1.2); conversion of nitriles to amides via hydrolysis or the Ritter reaction (Section 3.1.3); and ammonia surrogates for the synthesis of primary amides (Section 3.1.4).

#### 3.1.1 Unactivated Esters and Lactones

Methyl and ethyl esters of carboxylic acids are readily available starting materials that can be converted into amides under significantly milder conditions than their parent acids, while still offering high atom economy (see Table 5). In 2021, Nicholson et al.^118^ reported the adaptation of this approach to mechanochemical conditions (Scheme 40). The optimized protocol involved ball milling methyl or ethyl esters (1.2 equiv) with an amine and potassium *tert*-butoxide (0.85 equiv) in a VBM at 30 Hz for 1 to 2 h. A total of 78 amides was synthesized, with yields ranging from 11% to 98%. Notably, the method required no liquid additives, rendering it entirely solvent-free.

**Scheme 40:**
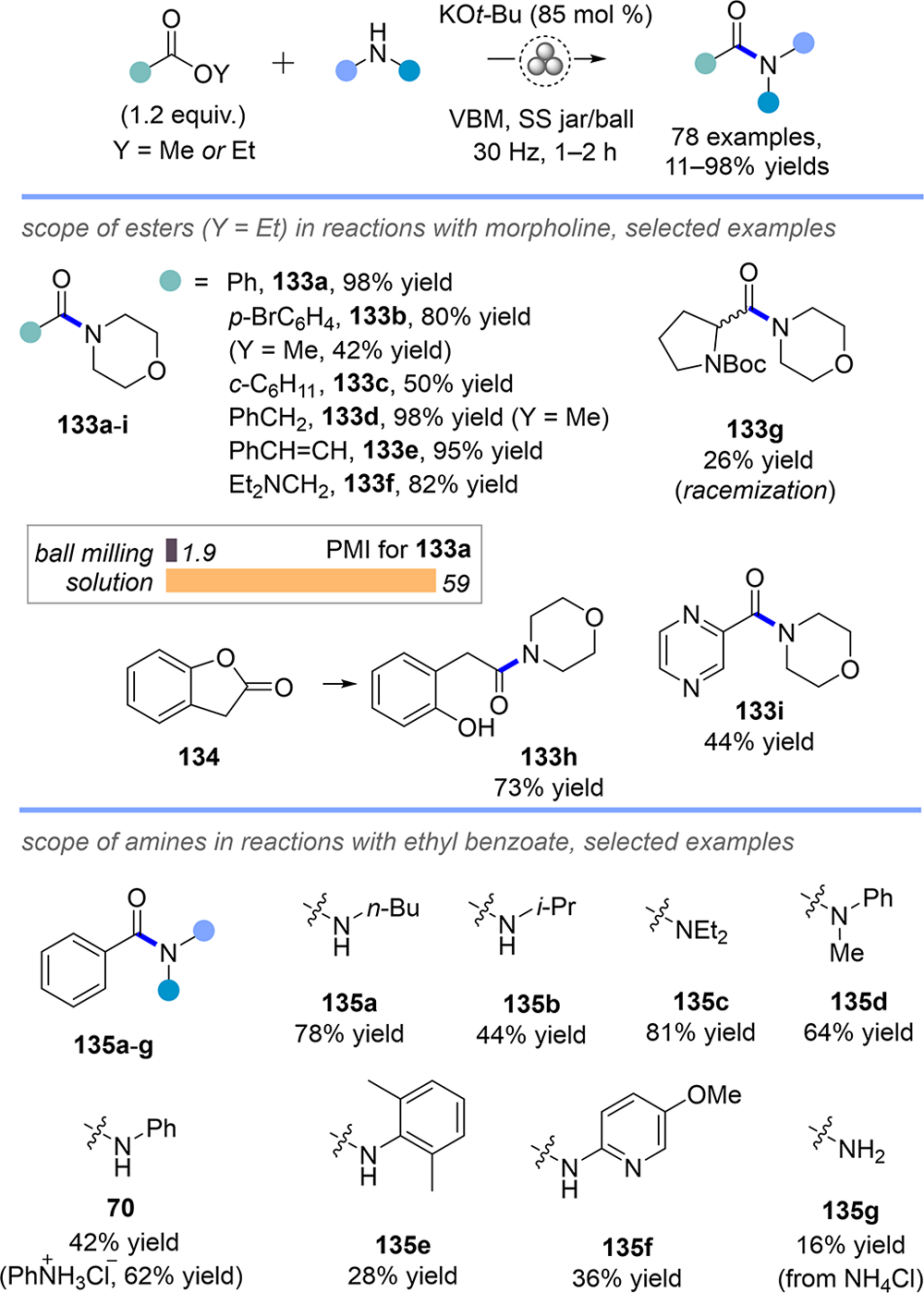
Direct Amidation of Unactivated Esters by Ball Milling, Selected Examples

In the model reaction between ethyl benzoate and morpholine yielding benzamide **133a**, potassium *tert*-butoxide was found to be critical for the reaction, outperforming all other tested bases, including a sibling sodium base. The optimal base loading of 0.85 equiv. gave the highest yield, although the mechanistic basis for this remains unclear. This value was validated across three independent laboratories. The nature of the ester leaving group strongly influenced reactivity: methyl, ethyl, benzyl, and phenyl benzoate esters gave high yields of **133a** (70–98%), whereas isopropyl esters were less efficient (42%), and *tert*-butyl and menthyl esters showed minimal reactivity (∼5%). These trends reflect classical steric and electronic effects.

Importantly, in contrast to solution-based methods, the aggregate state of the ester significantly affected reactivity: for instance, solid methyl 4-bromobenzoate afforded only 42% yield of the respective amide **133b** in reaction with morpholine, while its liquid ethyl analogue gave 80% under identical conditions.

In addition to esters of aromatic acids, the methodology was effective for a variety of unsaturated and aliphatic esters (examples: **133c**-**e**), including functionalized substrates such as ethyl *N*,*N*-diethylglycinate (amide **133f**). Ethyl Boc-l-prolinate yielded only 26% of the corresponding morpholide **133g** and underwent complete racemization. Heteroaromatic esters derived from pyridine, pyrazine, thiophene, and benzofuran were also successfully employed (e.g., **133i**). Furthermore, the method was extended to the ring-opening amidation of lactones (transformation of **134** into **133h**).

Amine reactivity followed conventional trends, with secondary amines being more reactive than primary ones, and alkyl amines outperforming anilines. Within the substrate scope, several aliphatic primary and cyclic secondary amines reacted efficiently with aromatic esters to give the corresponding amides **135a**-**g**. In contrast, sterically hindered amines and poorly nucleophilic aminopyridines required prolonged milling times (up to 2 h) and gave lower yields (in a range of 20–45%, e.g., **135b**, **135e**, **135f**).

Amine hydrochloride salts could be used in place of the free base, requiring an increased base loading (1.85 equiv of KO*t*-Bu). For instance, *N*-phenyl benzamide **70** was obtained in 62% yield from aniline hydrochloride, compared to 42% when using the free base. Additionally, ammonium chloride was tested as a surrogate for ammonia, affording benzamide **135g** in 16% yield under optimized conditions.

A highly valuable aspect of this study was the detailed reporting of substrate limitations (Scheme 41). Several compounds were found to be completely unreactive under the optimized conditions, even after extended milling times of up to 3 h or underwent undesired side reactions. Unreactive examples included sterically hindered substrates such as the methyl ester of mesitoic acid, 1-adamantylamine, 2,2,6,6-tetramethylpiperidine, and electron-deficient and weak nucleophiles, such as amides. Surprisingly, the nucleophilic and sterically unhindered *O*-methyl hydroxylamine was unreactive. Additionally, some esters underwent competing processes. For instance, ethyl 2-bromothiazole-5-carboxylate (**136**) reacted via S_N_Ar substitution, compound **137** decomposed upon addition of potassium *tert*-butoxide, and ester **138** underwent saponification followed by decarboxylation.

**Scheme 41:**
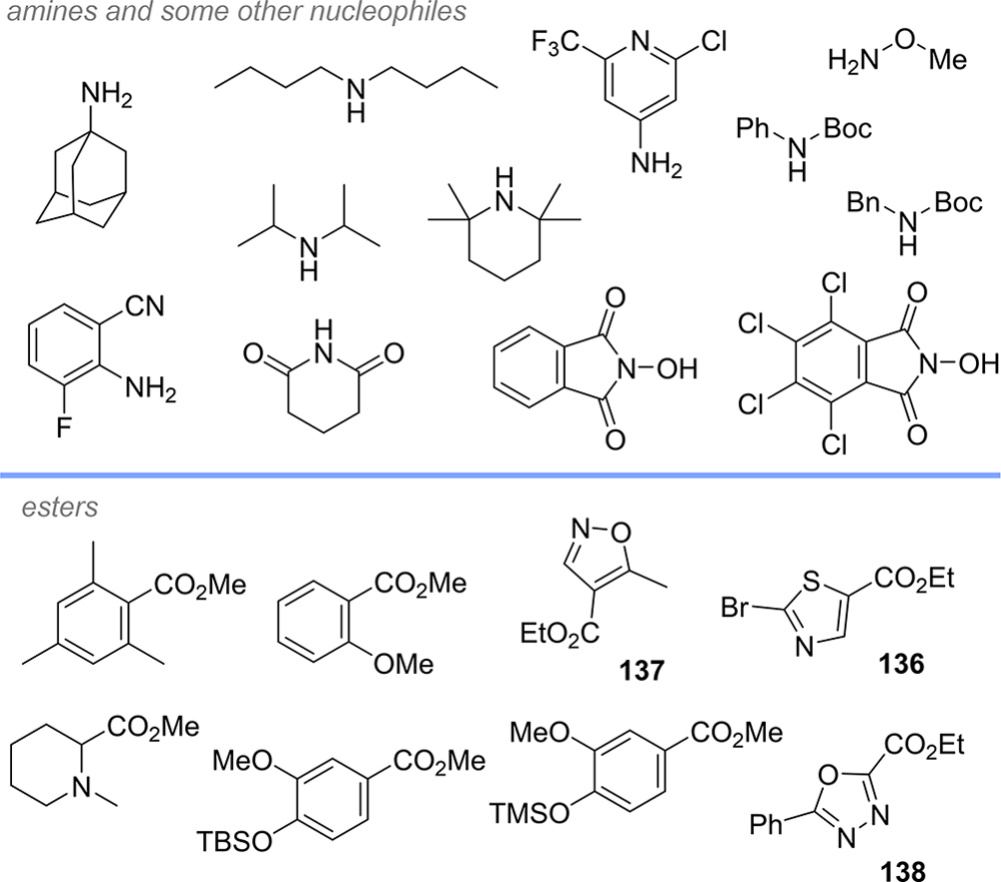
Direct Amidation of Unactivated Esters: Limitations

Beyond its broad substrate scope, the method has been successfully applied to the synthesis of several pharmaceutically and agriculturally relevant compounds (Scheme 42). These include the antidepressant moclobemide (**2**), the local anesthetic lidocaine (**139**), the respiratory stimulant coramine (**140**), the fungicide fenfuram (**141**), and an analogue of the MMP-13 inhibitor CL-82198 (**142**). In addition to its synthetic utility, the method demonstrated scalability. The preparation of moclobemide was scaled up 10-fold, yielding 2.4 g of the target compound in 90% yield using a 25 mL milling jar. A 20-fold scale-up of the model reaction between ethyl benzoate and morpholine was also achieved, affording 3.21 g of product **133a** in 83% yield after 2 h of milling. Additionally, in the synthesis of **133a** the method showed a reaction PMI of 1.94, significantly lower than a benchmarking solution-based method (reaction PMI = 59).

**Scheme 42:**
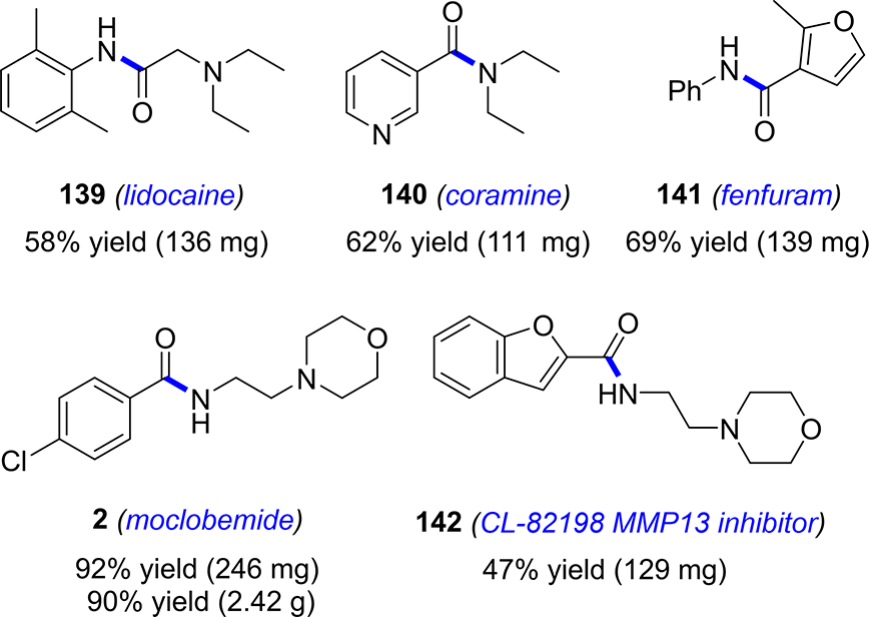
Direct Amidation of Unactivated Esters: Applications and Scalability

Subsequently, the method was successfully adapted to reactive extrusion (Scheme 43).^120^ Preliminary ball milling experiments showed that heating to 50 °C enabled a reduction in KO*t*-Bu loading from 85 to 60 mol %. The influence of the physical state of starting materials was systematically investigated by evaluating 36 ester-amine pairs under ball milling, encompassing all combinations of liquid and solid reactants (liquid–liquid, liquid–solid, solid–liquid, and solid–solid). Representative examples from each category (amides **133a**, **j-l**) were then translated to continuous extrusion. Notably, in combinations involving at least one liquid component, such as **133a** (liquid–liquid), **133j** (liquid–solid), and **133k** (solid–liquid), the addition of Na_2_SO_4_ as a grinding auxiliary significantly improved flowability within the extruder and enhanced material recovery. The scalability of the process was demonstrated through the continuous synthesis of amide **133l** on a process-relevant scale, achieving a throughput of 70 g·h^–1^ (1.68 kg·day^–1^). The product was isolated in 80% yield (490 g, 1.3 mol) following extraction and hot hexane filtration, comparable to the 81% yield obtained in a 50 mmol-scale extrusion run. Furthermore, it was shown that unreacted starting materials could be recovered via acid–base workup and potentially reused after purification.

**Scheme 43:**
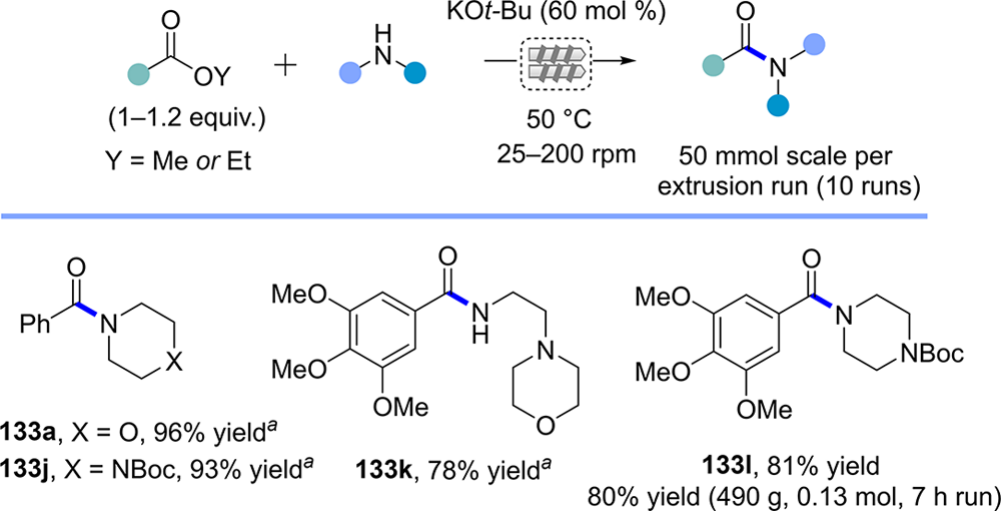
Mechanochemical Amidation of Esters Using Reactive Extrusion

a Na_2_SO_4_ (2 mass equiv) was used as a grinding additive.

Overall, potassium *tert*-butoxide-mediated amidation of esters exhibits a broad substrate scope, competitive atom economy, low reaction PMI (<2), and excellent scalability. The nearly half-kilogram production of amide **133l** marks the highest reported preparative scale for mechanochemical amide synthesis to date. This positions the method among the most advanced and industrially attractive mechanochemical approaches, offering a viable alternative to coupling-reagent-based methods. However, the method is unsuitable for base-sensitive or epimerization-prone substrates, which limits its broader applicability.

The ring-opening amidation of bioderived (*S*)-γ-hydroxymethyl-γ-butyrolactone (**143**, Scheme 44) in a planetary ball mill was described by Herrlé et al.^130^ For primary amines, the optimized reaction time was 47 min, achieved through 8 milling cycles of 5 min each, separated by 1 min pauses. The method demonstrated broad substrate scope, efficiently converting lactone **143** into the respective amides using primary alkylamines with chain lengths from C_10_ to C_19_. High conversions (88% to >97%) were obtained, with isolated yields ranging from 62% to 76%. The protocol was also extended to secondary amines following additional optimization. For example, the reaction with dimethylamine required 4 equiv of the amine and 71 min of milling to reach >97% conversion, affording a 77% yield.

**Scheme 44:**
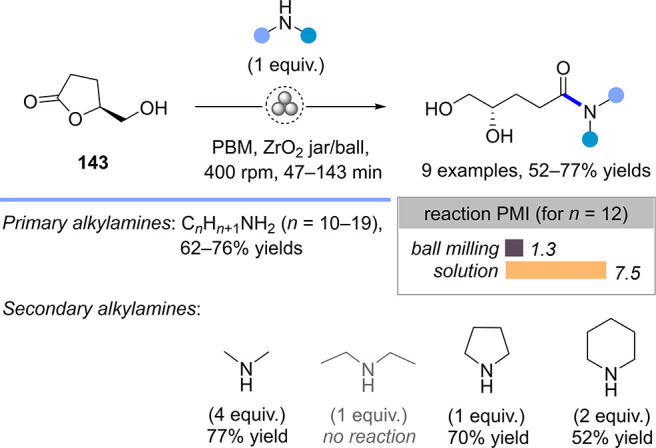
Mechanochemical Aminolysis of (S)-γ-Hydroxymethyl-γ-butyrolactone

The mechanochemical approach offered significantly improved reaction PMI values over the solution-based method (1.32–1.60 vs 6.94–8.1), no need for heating (room temperature vs 50 °C), and drastically reduced reaction times (47–143 min vs 24–48 h), while delivering comparable yields (62–76% vs 71–83%). Product isolation was also simplified: amides derived from longer-chain amines (C_16_–C_19_) were purified by straightforward recrystallization from ethanol, whereas shorter-chain analogues required flash chromatography. A similar approach was applied for the synthesis of aldonamides from unprotected glyconolactones^314^ and later employed by Crigna et al.^131^ to synthesize biobased amide surfactants through the ring-opening aminolysis of the γ-butyrolactone core in gluco-isosaccharinic acids derived from cellulose. The reactions were carried out with primary alkyl amines (C_12_, C_16_, C_18_) either by manual grinding or in a planetary ball mill, with water as LAG agent to promote homogenization.

Tomita et al.^132^ reported a single example of the one-pot mechanochemical synthesis of β-oxopropylcarbamate **145** via the ring-opening of cyclic carbonate **144a** with piperidine (Scheme 45). The carbonate intermediate was prepared through the silver-catalyzed fixation of gaseous CO_2_ with propargylic alcohol **144**.

**Scheme 45:**
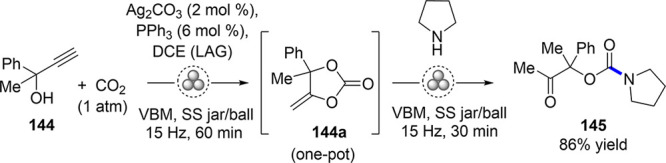
Mechanochemical Synthesis of Carbamate **145** via Fixation of Gaseous CO_2_ Followed by Ring-Opening

#### 3.1.2 Transamidation of Phthalimide

Among nonclassical approaches to amide bond formation, transamidation has emerged as a straightforward and versatile method for amide diversification.^133^ However, these transformations are kinetically and thermodynamically challenging because of the exceptional stability of the amide bond. Consequently, there several activating strategies are exploited, such as introducing electron-withdrawing substituents on the amide nitrogen, the use of strong bases to activate nucleophilic amine, or activation of the carbonyl group via acid catalysis.

In 2025, Morales-Manrique et al.^134^ reported a mechanochemical example of transamidation using various amines and phthalimide (Scheme 46), where reactivity was induced solely by mechanical energy without the need for catalysts, acids, or thermal activation. Ball milling phthalimide with primary or secondary amines in the presence of NaCl as a grinding auxiliary produced a series of monosubstituted phthalamides in 34–94% yields. Primary benzyl amines and 2-phenylethylamine consistently afforded the corresponding phthalamides (**146a**-**d**) in good to excellent yields (63–88%), whereas the sulfur-containing derivative **146e** was obtained in a modest 34% yield. Aliphatic amines and allylamine also reacted efficiently, producing phthalamides **146f**-**h** in 62–92% yields. Likewise, secondary amines performed well under the optimized conditions, delivering phthalamides **146i**-**l** in 39–98% yields. Importantly, the method demonstrated compatibility with structurally complex substrates, such as the drug trimetazidine, affording the corresponding phthalamide **146m** in a modest 36% yield. Moreover, the scalability of the protocol was confirmed by the gram-scale synthesis of **146a** (1.27 g, 71% yield). Notably, less nucleophilic aromatic amines (both primary and secondary), as well as sterically hindered aliphatic amines such as diisopropylamine, were found to be completely unreactive.

**Scheme 46:**
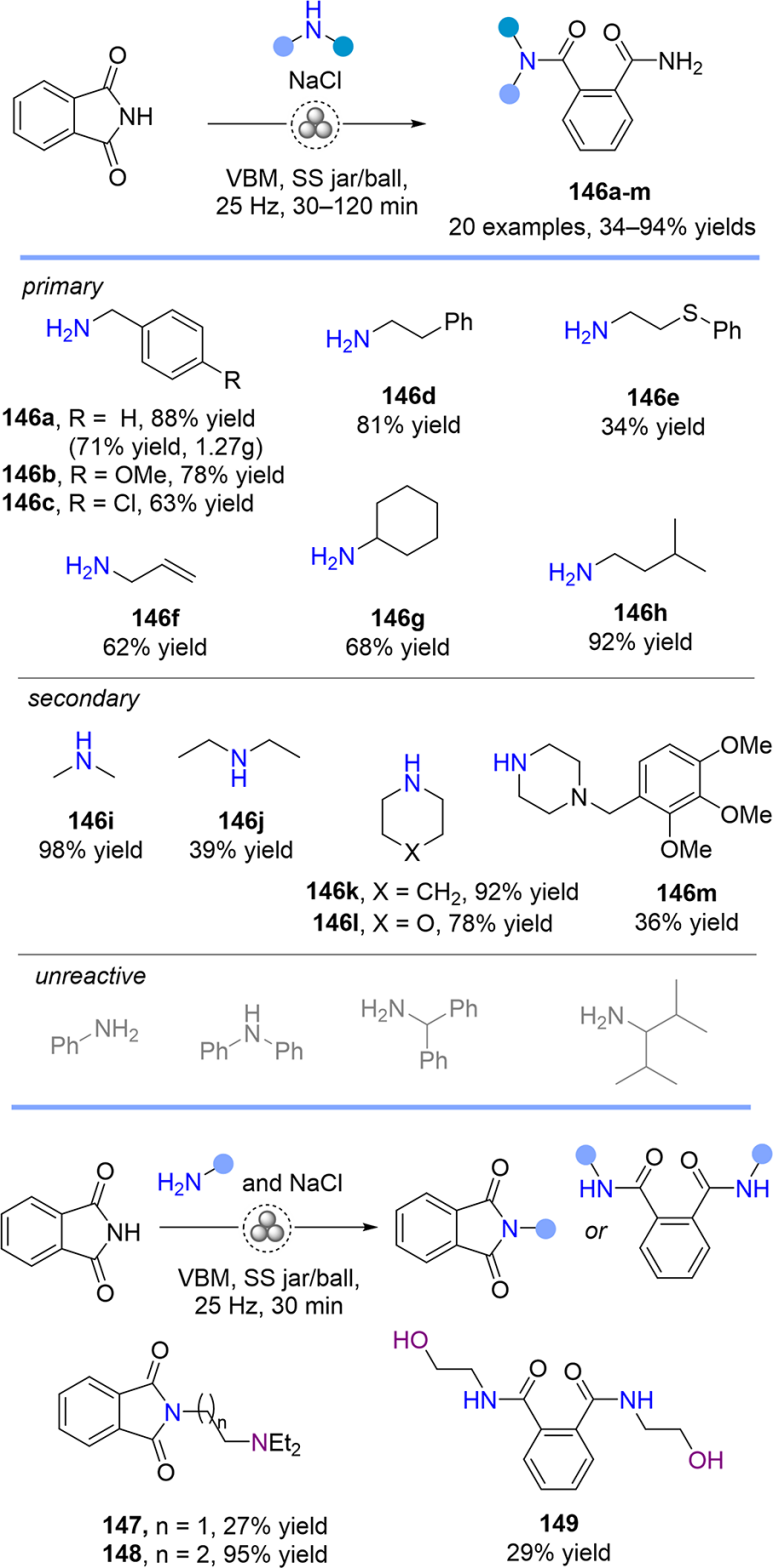
Mechanochemical Transamidation of Phthalimide

In contrast, the reaction of phthalimide with *N*,*N*-dimethylethane-1,2-diamine led to the formation of cyclic *N*-substituted phthalimide **147** as the main product, albeit in a modest 27% yield. When *N*,*N*-dimethylpropane-1,3-diamine was employed, the corresponding phthalimide **148** was obtained in an excellent 95% yield. In the crude reaction mixture, neither disubstituted phthalamides nor other products were detected. It was assumed that basic tertiary amino groups of the respective amines were responsible for the cyclizations leading to phthalimides.

The reaction with ethanolamine showed moderate conversion but produced exclusively the disubstituted phthalamide **149** in 29% yield, indicating selectivity toward nitrogen nucleophilic site rather than oxygen.

Overall, the developed protocol represents a room-temperature, catalyst-free, base-free and solvent-free mechanochemical transformation of phthalimide to mono-*N*-alkyl phthalamides. Notably, the formation of mono-*N*-alkyl phthalamides under mechanochemical conditions differs markedly with solution-based protocols, where *N*-substituted phthalimides are typically predominant products.

#### 3.1.3 Nitriles

The Ritter reaction forms amides through the reaction of nitriles with carbocation species, typically generated from tertiary alcohols or substituted olefins in the presence of a strong Brønsted acid catalyst.

In 2015, Dokli and Gredičak reported a mechanochemical adaptation of the Ritter reaction (Scheme 47).^135^ Sulfuric acid was identified as the most effective catalyst. In the reaction between benzonitrile and *tert*-butyl alcohol, the choice of milling materials significantly affected the conversion to benzamide **150**. The highest conversion (98% in 30 min) was achieved using a tungsten carbide ball in a Teflon vial. While corundum balls provided similarly high conversions (up to 94%), their rapid wear rendered them unsuitable. In contrast, a lighter Teflon ball led to significantly reduced efficiency, yielding only 73% conversion after 60 min.

**Scheme 47:**
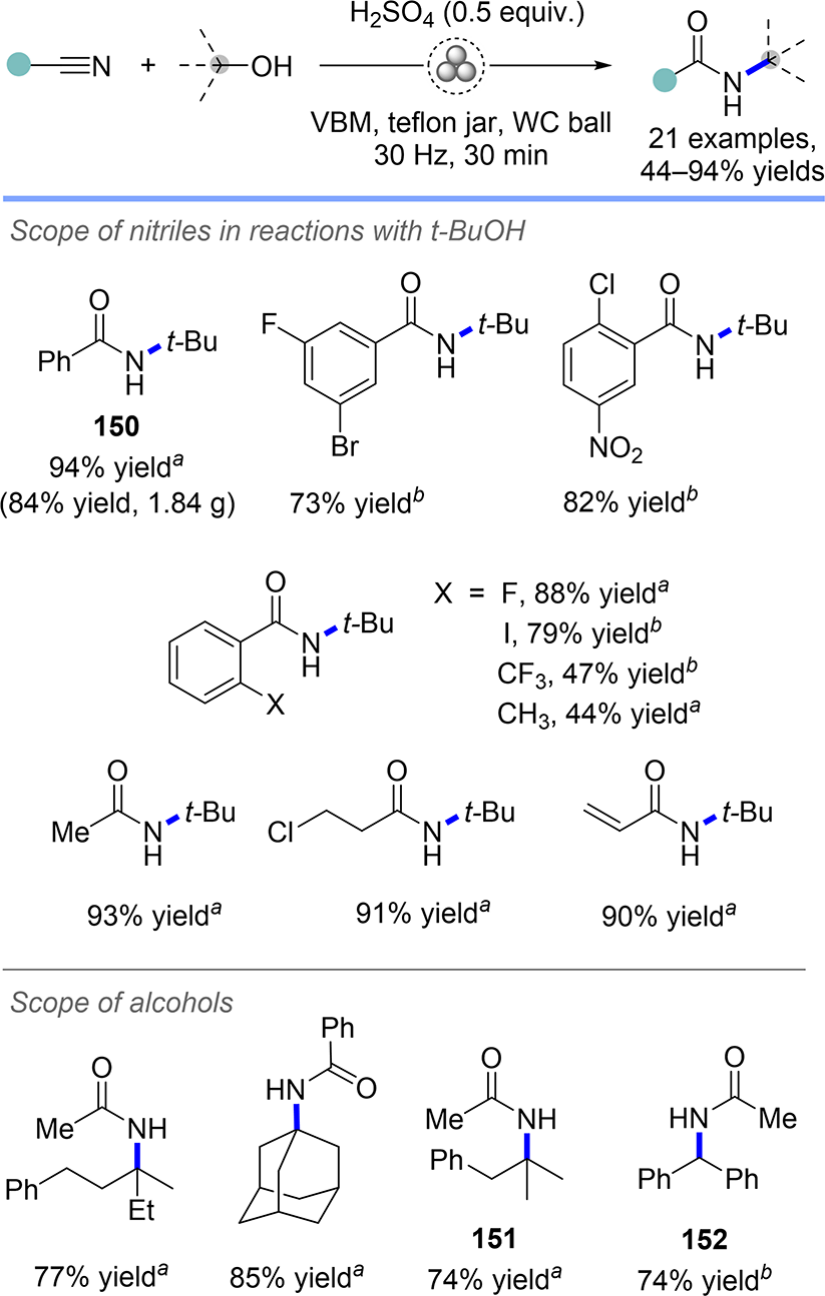
Mechanochemical Ritter Reaction. Selected Examples

a No LAG.
b LAG (MeNO_2_, 1 equiv).

The method was applied to the synthesis of 21 functionalized amides at room temperature, delivering yields of 44–94% within short reaction times (30–60 min). The substrate scope included various substituted benzonitriles, alkyl nitriles, and acrylonitrile, with most examples yielding over 70%. Exceptions included *ortho*-substituted benzonitriles bearing trifluoromethyl or methyl groups, likely due to steric hindrance. The use of a polar, non-nucleophilic LAG additive (nitromethane, η = 0.26 μL·mg^–1^) proved essential for solid substrates like 2-iodobenzonitrile, likely by stabilizing the carbenium ion intermediate.

As for alcohol partners, four tertiary alcohols were tested and generally gave good yields (above 74%). However, 2-methyl-1-phenylpropan-2-ol led primarily to olefin formation via elimination. Increasing the amount of acetonitrile to 5 equiv. afforded amide **151** as the sole product in 74% yield. Secondary benzylic alcohols that generate stabilized carbocations also reacted efficiently, exemplified by the formation of amide **152**. The gram-scale reaction between benzonitrile and *tert*-butyl alcohol afforded **150** in 84% isolated yield.

An interesting case of mechanochemically assisted hydrolysis of nitriles into amides was reported by Bolm et al.^136^ This study aims to model a prebiotic pathway for the formation of α-amino acid derivatives by demonstrating that mechanochemical activation of iron cyanide complexes can generate α-aminonitriles (e.g., **153**, Scheme 48), which can subsequently be hydrolyzed in a ball mill under plausible early Earth conditions to yield α-amino amides (e.g., **154**). This approach provides a dry-phase, impact-driven scenario for peptide precursor formation, potentially relevant to the origin of life.

**Scheme 48:**
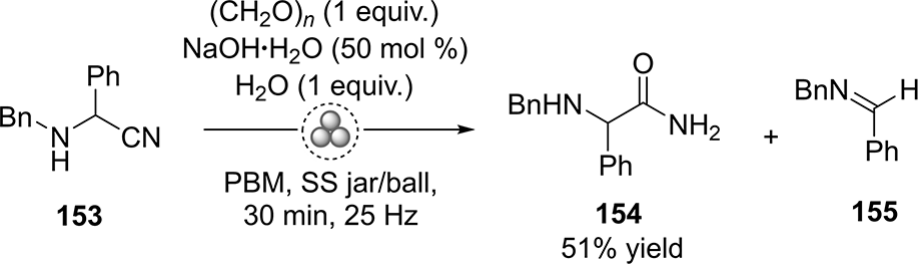
Mechanochemical Hydrolysis of an α-Aminonitrile as a Model for the Prebiotic Formation of α-Amino Amides

Initial attempts using basic hydrolysis with sodium hydroxide monohydrate or DBU, combined with urea-hydrogen peroxide complex (UHP), resulted in low yields (10–43%) of **154** and significant formation of the imine byproduct **155**. The breakthrough came with the use of paraformaldehyde, following a method originally developed for solution-phase chemistry. Ball milling a mixture of α-aminonitrile **153**, paraformaldehyde and NaOH·H_2_O for 30 min afforded the amide **154** in 51% yield.

#### 3.1.4 Surrogates of Ammonia

Preparation of primary amides requires ammonia, which is gaseous at ambient conditions. Although gaseous reactants can be used in mechanochemical reactions,^137^ it is more practical to use solid reagents that generate gases *in situ*. In 2021 Gómez-Carpintero et al.^138^ reported the conversion of ethyl esters into primary amides using ammonia generated *in situ* from the reaction of magnesium and calcium nitrides with ethanol (Scheme 49). The protocol was a mechanochemical adaptation of a previously published method that required harsh conditions, specifically heating in a sealed tube at 80 °C for 24 h.^139^

**Scheme 49:**
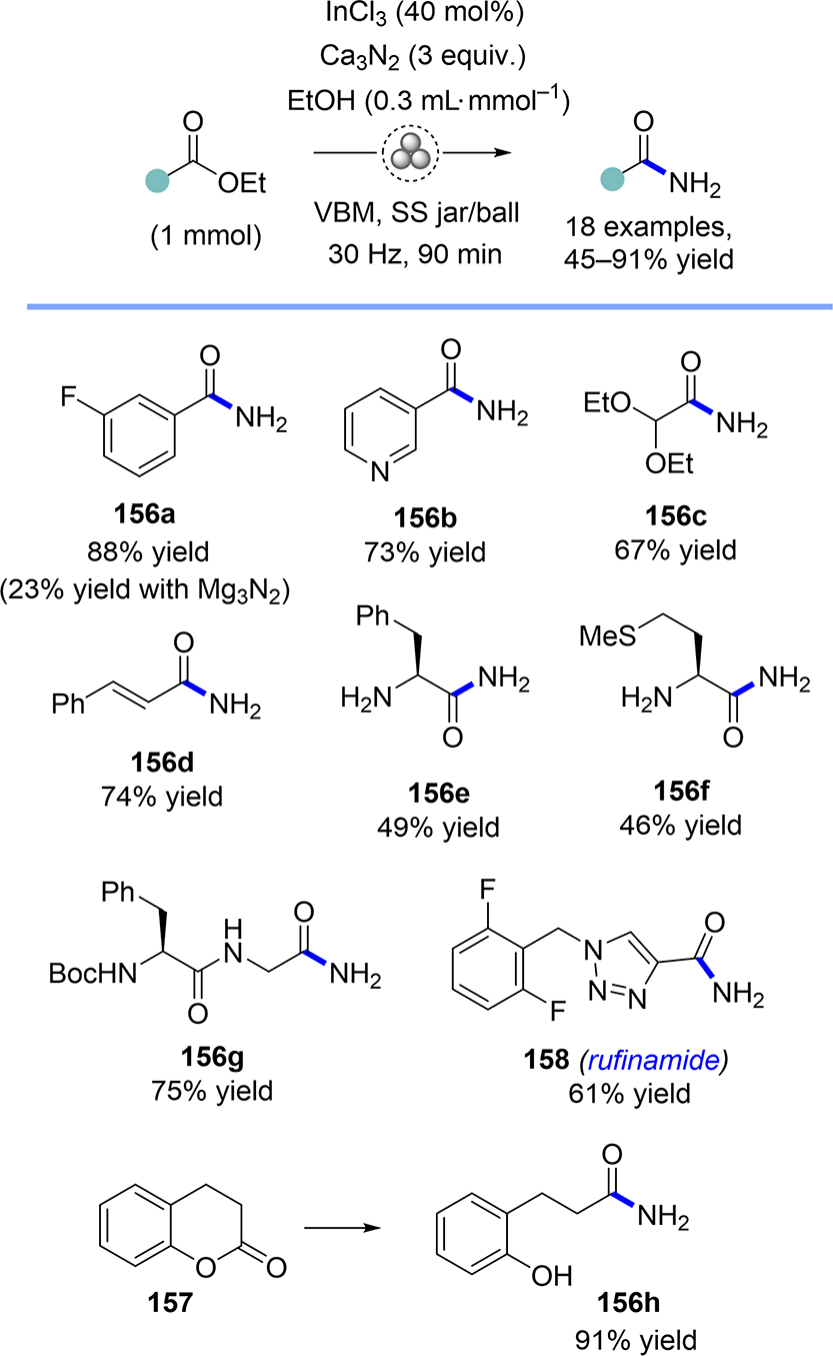
Mechanochemical Synthesis of Primary Amides from Ethyl Esters and Lactones Using Mg_3_N_2_ and Ca_3_N_2_ as Ammonia Surrogates

The reactions were performed in a VBM at room temperature and a milling frequency of 30 Hz. In the reaction of ethyl 3-fluorobenzoate with Mg_3_N_2_ (5 equiv) to give amide **156a**, the presence of a Lewis acid catalyst was found essential. ZnCl_2_ and InCl_3_ (both at 20 mol %) were the most effective, achieving conversions of 85% and 91%, respectively. Notably, replacing the nitride with alternative ammonia sources such as ammonium chloride or acetate resulted in no product formation. Although high conversions were observed with Mg_3_N_2_, the generation of magnesium salts complicated the isolation of product **156a**, resulting in only 23% yield. Replacing the magnesium compound with the calcium analogue proved beneficial and formed the basis of the final protocol, which involved ball milling the ester, Ca_3_N_2_ (3 equiv), and InCl_3_ (40 mol %) in a 10 mL stainless steel jar with a single 15 mm stainless steel ball at 30 Hz for 90 min, using ethanol (0.3 mL per 1 mmol of ester) as the liquid additive. The workup consisted of treatment with water and extraction with ethyl acetate.

The preparative scope of the method was demonstrated through the conversion of various ethyl esters into primary amides with yields ranging from 45% to 91%. The scope included primary amides of benzoic acids (3 examples, such as **156a**), heteroaromatic acids (3 examples, e.g., nicotinamide **156b**), aliphatic and functionalized aliphatic acids (5 examples, e.g., **156c**), cinnamic acid (**156d**), amino acids (phenylalanine and methionine, **156e** and **156f**), and *N*-Boc dipeptides with C-terminal glycine esters (3 examples, e.g., **156g**). The preservation of stereochemical integrity of α-stereocenters was demonstrated by measuring the optical rotation of **156e**, which matched published values.

Lactone **157** was also converted into the corresponding amide **156h** in 91% yield. Finally, the authors applied their method to the synthesis of the antiepileptic drug rufinamide (**158**), achieving a 61% yield under optimized conditions, highlighting the potential of the method for preparing pharmaceutically relevant compounds.

Although not encountered in this particular study, the use of magnesium nitride for converting esters into amides in methanol at 80 °C has been reported to cause actual explosions, especially at a relatively large scale (with ∼ 1 g of Mg_3_N_2_).^140^ This safety hazard, combined with the need for excess nitride beyond stoichiometric amounts, may limit the broader applicability and scalability of the method.

The use of ammonium salts as amine surrogates in mechanochemical amidation has been demonstrated in both fine chemical synthesis and materials science. Thus, Nicholson et al.^118^ employed ammonium chloride for the amidation of ethyl benzoate, as previously discussed (see Scheme 40). In materials science, Qu et al.^141^ developed a mechanochemical method for the surface functionalization of nanodiamonds (NDs) using ammonium chloride as the amidation reagent and sodium chloride as a grinding auxiliary (Scheme 50). Initially, potassium permanganate was used in solution to oxidize the NDs surface, introducing carboxyl groups. The resulting material was then ball-milled with NH_4_Cl and NaCl in a 1:10:5 weight ratio using a 100 mL zirconium oxide milling chamber.

**Scheme 50:**
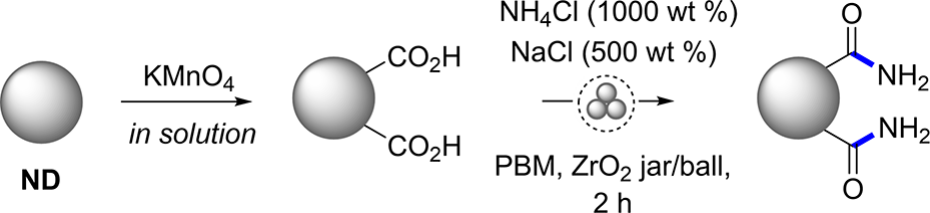
Mechanochemical Amidation of Nanodiamonds

This protocol enabled efficient amidation within 2 h, achieving high surface functionalization levels (up to 173.7 μmol·g^–1^) without compromising the crystalline structure of the NDs. XRD confirmed retention of the characteristic diamond peaks for the [111] and [220] planes, and TEM analysis showed no significant change in particle size or morphology, with the amidated NDs displaying uniform dispersion in colloidal solutions. The process was successfully scaled up to the hundred-gram scale.

### 3.2 Metal-Mediated Transformations

A number of studies have demonstrated the mechanochemical preparation of amides by employing stoichiometric organometallic reagents or transition metal catalysis, primarily via C–H activation reactions. These strategies rely on fundamentally different bond disconnections compared to the traditional amide synthesis route involving coupling of amines with carboxylic acids or their derivatives, enabling alternative routes to amide products. Consequently, these mechanochemical approaches are complementary to classic methods, offering valuable alternatives in cases where traditional routes encounter limitations due to functional group incompatibility, low reactivity, or challenging substrates. Moreover, transition-metal catalysis under solvent-free mechanochemical conditions often provides improved green chemistry characteristics, operational simplicity, and reduced reaction times, further highlighting the attractiveness of this synthetic strategy.

#### 3.2.1 Transition Metal-Mediated C–H Activation

Direct C–H functionalization offers a conceptually elegant strategy for forming C–N bonds by bypassing the need for substrate prefunctionalization. The adaptation of transition metal-catalyzed C–H activation approaches to mechanochemical conditions has emerged as a particularly fruitful area of research. These methods exploit the ability of transition metals to selectively activate otherwise inert C–H bonds, enabling efficient and atom-economical construction of amide linkages. To date, successful mechanochemical C–H amidation has been demonstrated using iridium, rhodium, cobalt, and iron catalysts, with dioxazolones or organic azides serving as nitrogen sources (Scheme 51).

**Scheme 51:**
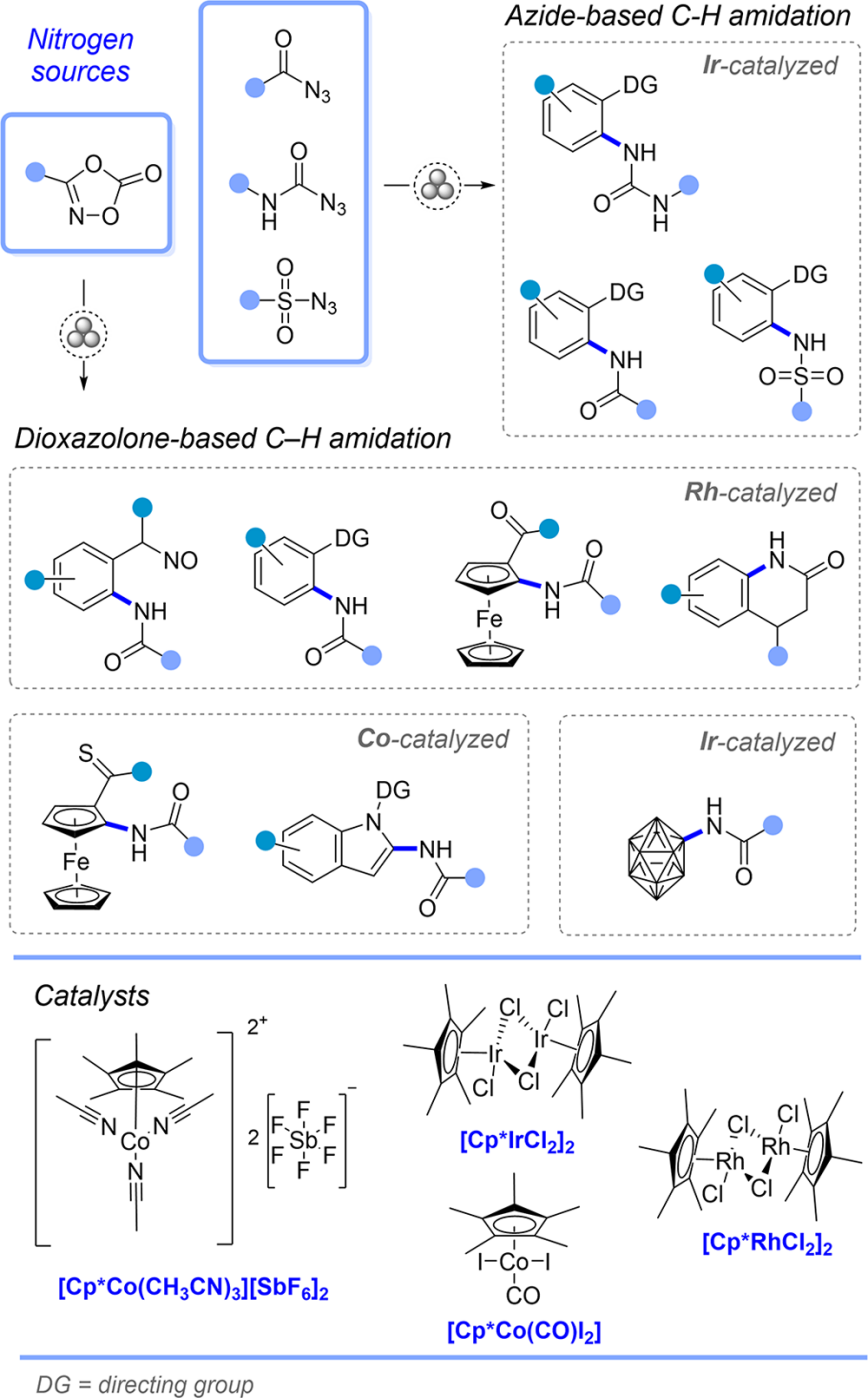
Synthesis of Amides via Mechanochemical Transition Metal-Mediated C–H Activations

The first example of aromatic C–H amidation with dioxazolones **159** as a nitrogen source under mechanochemical conditions was reported by Hermann and Bolm in 2017 (Scheme 52, A).^142^ The adaptation of solution-based conditions^143^ to a ball mill gave a satisfactory 76% yield of the *ortho*-amidated product **160** (DG = CONH*t*-Bu, R^1^ = Me) in the reaction between *N*-(*tert*-butyl)­benzamide and methyl-1,4,2-dioxazol-5-one **159** (R^1^ = Me). With further optimization, ball milling of [Cp*RhCl_2_]_2_ (2.5–5 mol %) and AgSbF_6_ (10 mol %) in combination with AgOAc (10 mol %) as an additive for 99 min at 30 Hz using a 25 mL ZrO_2_ vessel charged with a single ZrO_2_ milling ball, enabled the synthesis of versatile amides **160** from the reaction of dioxazolones with arenes. Using methyl-1,4,2-dioxazol-5-one **159** (R^1^ = Me), a total of 12 amides were synthesized in yields ranging from 38% to >99%, with three additional dioxazolones **159** (R^1^ = Et, *t*-Bu, Ph) affording 50–58% yields. Interestingly, Ir and Ru catalysts were inactive in the reaction. Scaling up to the gram scale (10 mmol) resulted in 84% yield of **160** (DG = CONH*t*-Bu, R^1^ = Me). Preliminary mechanistic investigations confirmed that the mechanism is similar to the analogous process in organic solvents. However, compared to the parent solution approach, the method delivered high yields at similar catalyst loadings at much shorter reaction times (99 min vs 12 h), avoiding external heating.

**Scheme 52:**
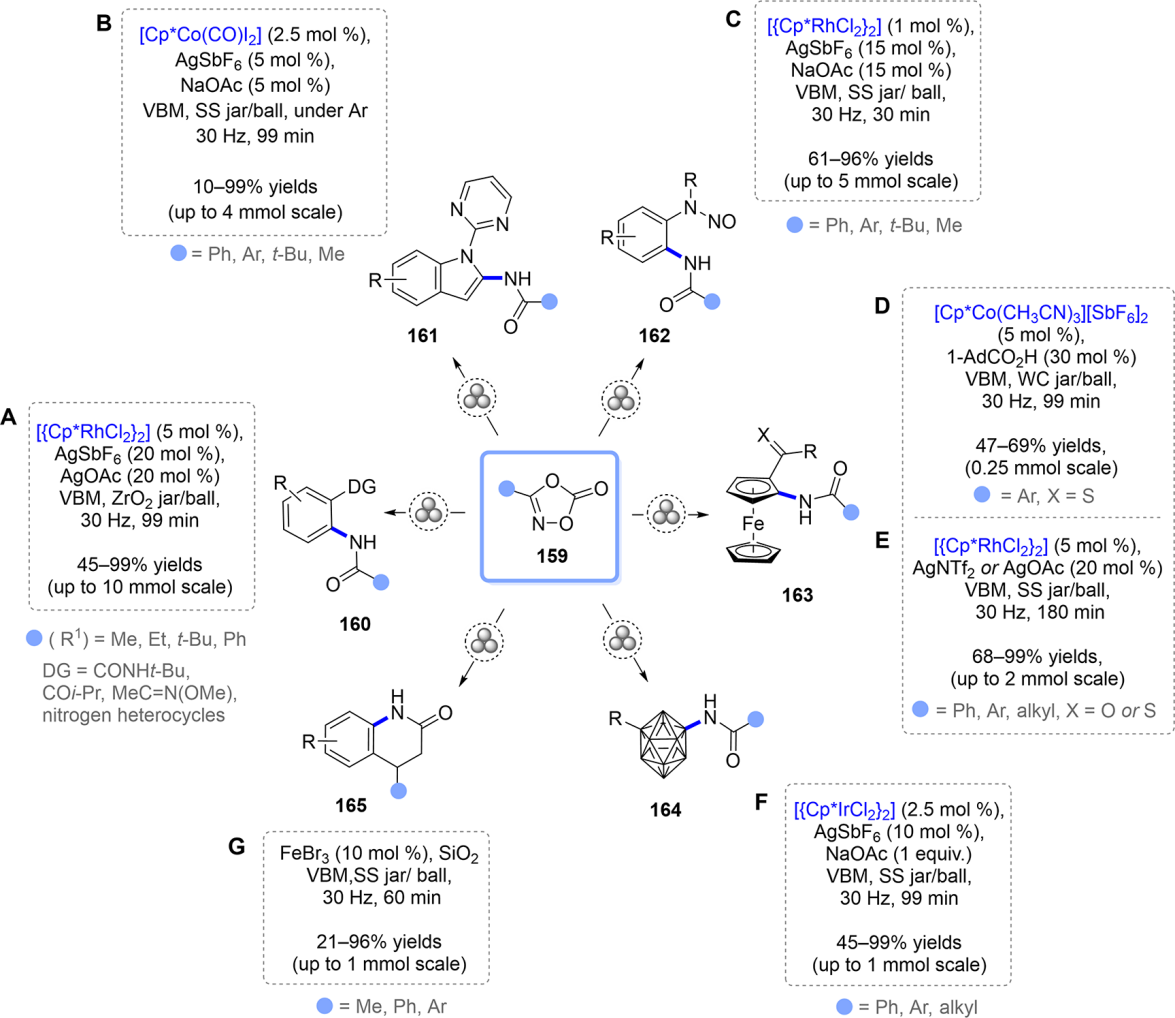
State-of-the-Art Mechanochemical Transition Metal-Mediated C–H Activations for the Synthesis of Amides Using Dioxazolones **159** as a Nitrogen Source

In 2018, the same group reported a cobalt-catalyzed, pyrimidine-directed C–H amidation for the synthesis of functionalized indole scaffolds **161** (Scheme 52, B).^144^ The synthetic utility was demonstrated through the successful functionalization of 20 indole derivatives, using [Cp*Co­(CO)­I_2_] (2.5 mol %) as the catalyst in the presence of AgSbF_6_ (5 mol %) and NaOAc (5 mol %) as additives. A temperature increase of up to 35 °C was recorded on the outer wall of the milling vessel, which was suggested to contribute to the reaction efficiency. The process was scalable, delivering 83% yield on the gram scale. As with previous examples, the mechanochemical approach offered significantly shorter reaction times compared to the corresponding solution-based methods.

Rhodium­(III)-catalyzed C–H amidation of *N*-nitrosoanilines under ball-milling conditions was reported by Li and Wang in 2018 (Scheme 52, C).^145^ During optimization, it was found that Ru, Ir, Co and Pd catalysts were inactive, and that reducing the Rh catalyst loading from 2.5 mol % to 1 mol % had minimal impact on the yield. However, further reduction to 0.5 mol % led to a significant decrease in product formation. Using various dioxazolones **159**, a range of *ortho*-amidated products **162** was prepared (25 examples, 61–96% yields). The reaction showed good functional group tolerance, accommodating both electron-donating and electron-withdrawing substituents in *N*-nitrosoanilines. Scaling up to the gram scale (5 mmol) resulted in a yield of 89%. Compared to the conventional solution approach, this method allowed the synthesis of 2-aminoanilides in high yields at lower catalyst loadings, avoiding external heating and reducing the reaction time from 6 h to 30 min. The products could be further converted to pharmaceutically valuable benzimidazole derivatives through a one-pot two-step synthesis under ball-milling conditions.

Mechanochemical C–H amidation with dioxazolones was further extended to the synthesis of functionalized ferrocenes **163** (Scheme 52, D and E). Yetra et al.^146^ employed a cobalt catalyst (5 mol %) with 1-adamantanecarboxylic acid (30 mol %) as an additive to achieve amidation of thiocarbonyl-substituted ferrocenes. The reactions were conducted in a 10 mL tungsten carbide jar using a single 10 mm ball of the same material. Although the protocol was originally developed in solution and not specifically optimized for mechanochemical conditions, six amidated ferrocenes **163** were successfully synthesized in 47–69% yields. Notably, the mechanochemical method eliminated the need for chlorinated solvents (DCM, DCE) used in the solution-phase counterpart.

Li et al.^147^ reported a related transformation employing a rhodium catalyst in combination with AgOAc (20 mol %) as an additive (Scheme 52, E), affording 20 examples of amido-functionalized ferrocenes **163**. The method demonstrated broad substrate scope, accommodating both electron-donating and electron-withdrawing substituents on the dioxazolone ring, as well as aliphatic dioxazolones. Notably, iridium and cobalt catalysts were found to be inactive under these conditions. The protocol was successfully scaled up to a 2 mmol reaction, delivering the target product in 94% yield.

In 2021, Han et al.^148^ applied a mechanochemical Ir­(III)-catalyzed process to achieve B–H amidation of *o*-carboranes with dioxazolones **159** (Scheme 52, F). The protocol afforded 34 amidated carboranes **164** in 45–99% yields, showing broad compatibility with both aryl and aliphatic dioxazolones and variously substituted carborane substrates. Notably, Rh catalysts led to undesired decarboxylation, and NaOAc was essential to suppress side reactions and enable selective B–H activation. Mechanistic studies supported the formation of an iridacycle intermediate. The method outperformed analogous reactions in solution or under microwave conditions, and was scalable to the 1 mmol scale with a 93% yield.

In 2020, Shi et al.^149^ used readily available and inexpensive iron­(III) bromide as a catalyst (10 mol %) to perform intramolecular C–H amidation under mechanochemical conditions (Scheme 52, G). Milling of dioxazolone substrates with silica and FeBr_3_ enabled the preparation of dihydro-2­(1*H*)-quinolinones **165** in yields up to 96%. Copper and nickel catalysts, as well as added ligands, proved ineffective. Electron-donating substituents in the aromatic ring improved reactivity, with the 4-methoxy substrate giving the highest 96% yield. Mechanistic studies revealed two competing cyclization pathways, electrophilic spirocyclization and S_E_Ar-type reaction.

Acyl and sulfonyl azides (**166**–**168**) represent alternative nitrogen sources known to perform directing group-controlled *ortho*-C–H amidation of aromatic compounds under ball milling conditions (Scheme 53).

**Scheme 53:**
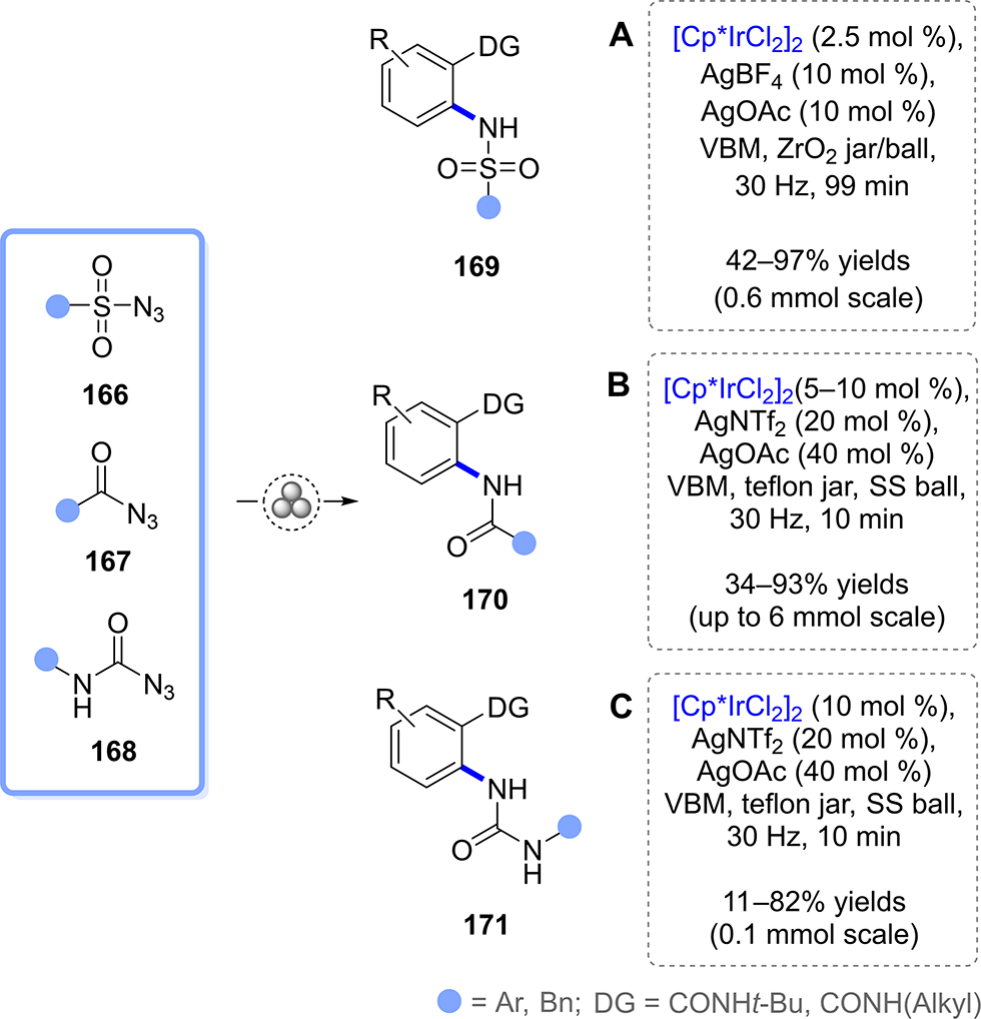
Mechanochemical Transition Metal-Mediated C–H Activations for the Synthesis of Amides Using Acyl and Sulfonyl Azides Nitrogen Sources

In 2016, Hermann et al.^150^ reported a seminal study on iridium-catalyzed ortho-amidation of benzamides with sulfonyl azides **166** under solvent-free mechanochemical conditions (Scheme 53, A). The reaction proceeded efficiently in a ball mill, affording *ortho*-amidated products **169** in high yields (42–97%) across a broad substrate scope. Substituents on the benzamide core had minimal impact on reactivity with the exception of strongly electron-withdrawing groups like trifluoromethyl, which gave moderate yields. Various *N*-substituents in the directing group, including *tert*-butyl, *n*-butyl, isopropyl, and cyclohexyl, were well tolerated. The method offered notable advantages over the solution-based counterpart, such as solvent-free operation, elimination of external heating, and significantly shorter reaction times (99 min vs 12 h).

This methodology was further extended in 2021 to include acyl and carbamoyl azides (Scheme 53, B and C).^151^ Ball milling at 30 Hz for just 10 min enabled the synthesis of 12 *ortho*-functionalized amides **170** from acyl azides **167** in 34–93% yields, and a successful gram-scale reaction was demonstrated. Notably, the use of carbamoyl azides **168** revealed an unexpected increase in reactivity under mechanochemical conditions, consistently affording higher yields of **171** (11–82%, 8 examples) than reactions performed in DCE solution.

In summary, mechanochemical C–H activation approaches represent more efficient and sustainable alternatives to traditional solution-based methods, as they provide the same or better yields while excluding harmful halogenated solvents, do not require heating, and proceed much faster than solution-based analogues, shortening the reaction time from 6–12 h to 10–180 min. Mechanistic insights suggest that these solid-state reactions often proceed via similar pathways to their solution-phase analogues, typically involving cyclometalated intermediates. Nevertheless, current limitations persist, including reliance on silver salts or similar additives, dependence on rare and costly transition metals such as rhodium and iridium, and operation predominantly at small scale. In this regard, the demonstration of cobalt-catalyzed transformations, and especially the use of earth-abundant and inexpensive iron, are notable. As such, most mechanochemical C–H amidation protocols reported to date should be viewed as proof-of-concept demonstrations. Further research, particularly in terms of scalability, catalyst optimization, and additive minimization, is necessary before these methods can reach the maturity.

#### 3.2.2 Miscellaneous Metal-Mediated Transformations

Apart from the mainstream C–H activation strategies, other metal-mediated methodologies remain currently limited to a few seminal examples presented here, involving functional group interconversion and nucleophilic addition of a stoichiometric organometallic reagent.

Mechanochemical synthesis of *N*-aryl amides via Ru-catalyzed dehydrative cross-coupling of unprotected phenols with primary amides was demonstrated in 2025 by Mkrtchyan et al.^152^ (Scheme 54).

**Scheme 54:**
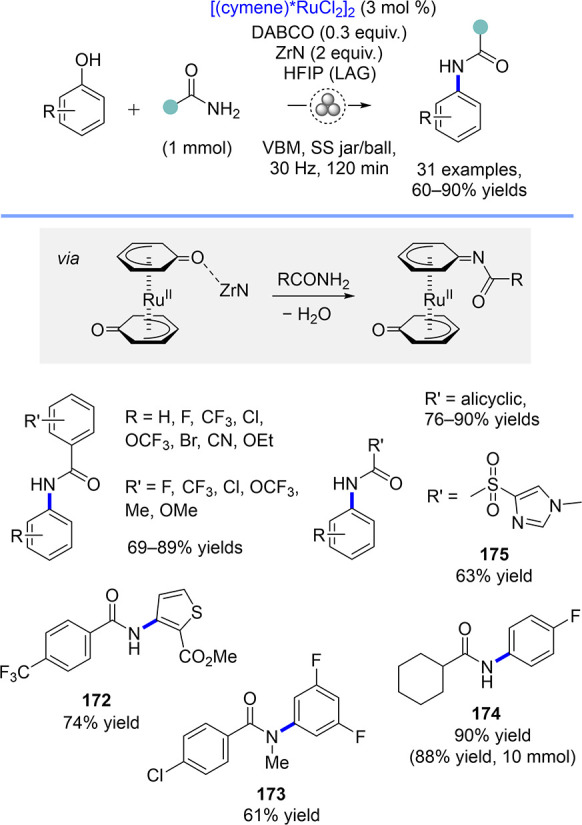
Mechanochemical Synthesis of *N*-Aryl Amides via Ru-Catalyzed Dehydrative Cross-Coupling of Unprotected Phenols with Primary Amides. Selected Examples

Phenols are renewable and sustainable aromatic feedstocks, readily available from sources such as lignin biomass. cross-coupling reactions involving phenols are notably challenging due to the high bond dissociation energy (BDE > 450 kJ·mol^–1^) of the C­(sp^2^)–O bond. This obstacle can be overcome by forming transient *η* ^5^-phenoxo-Ru complexes that activate the phenolic substrate.^153^ Building on this strategy, ball milling of phenols with primary amides in the presence of a Ru catalyst (3 mol %), DABCO as a basic additive, and zirconium nitride as a highly oxophilic solid promoter enabled formal nucleophilic substitution of the phenolic hydroxyl group. Phenols and amides bearing electron-donating and electron-withdrawing substituents in various positions on the aromatic ring furnished a broad range of *N*-aryl amides in 69–89% yields. The method was further extended to include heterocyclic (e.g., compound **172**, 74% yield) and alicyclic amides (76–90% yields). Reactions with less reactive secondary amides gave the corresponding tertiary *N*-aryl amide **173** in 61% yield, as well as an *N*-aryl sulfonamide analogue **175** was synthesized in 63% yield. Scale-up of the reaction to 10 mmol provided compound **174** in 88% yield.

Later, the same group developed mechanochemical methods to generate highly reactive acylpyridinium intermediates from pyrylium tetrafluoroborate.^154,155^ These acylpyridinium salts were subsequently applied in trifluoromethylation reactions^154^ and in the synthesis of biaryl ketones.^156^ The latter work is notable as it demonstrates carbonylation with Mo­(CO)_6_ to form the amide bond in acylpyridinium intermediates **176** generated *in situ* (Scheme 55). This example illustrates the feasibility of amide synthesis via metal-mediated carbonylation using solid CO surrogates under mechanochemical conditions.

**Scheme 55:**
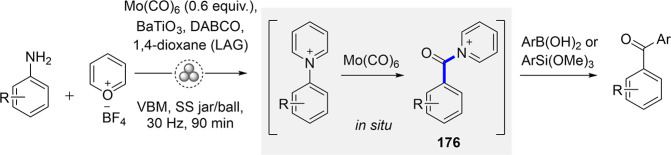
Generation of Reactive Acyl Pyridinium Salts via Carbonylation with Mo­(CO)_6_

Mechanochemical activation of metals enables the generation of organometallic reagents that are more resistant to atmospheric oxygen and moisture, allowing their preparation without the need for an inert atmosphere.^157^ In 2023, Takahashi et al.^158^ reported a mechanochemical method for generating arylmanganese nucleophiles from aryl halides and unactivated manganese metal in air. These manganese-based carbon nucleophiles were subsequently applied in one-pot addition reactions with electrophiles such as phenyl and cyclohexyl isocyanates, yielding a variety of amide products (Scheme 56).

**Scheme 56:**
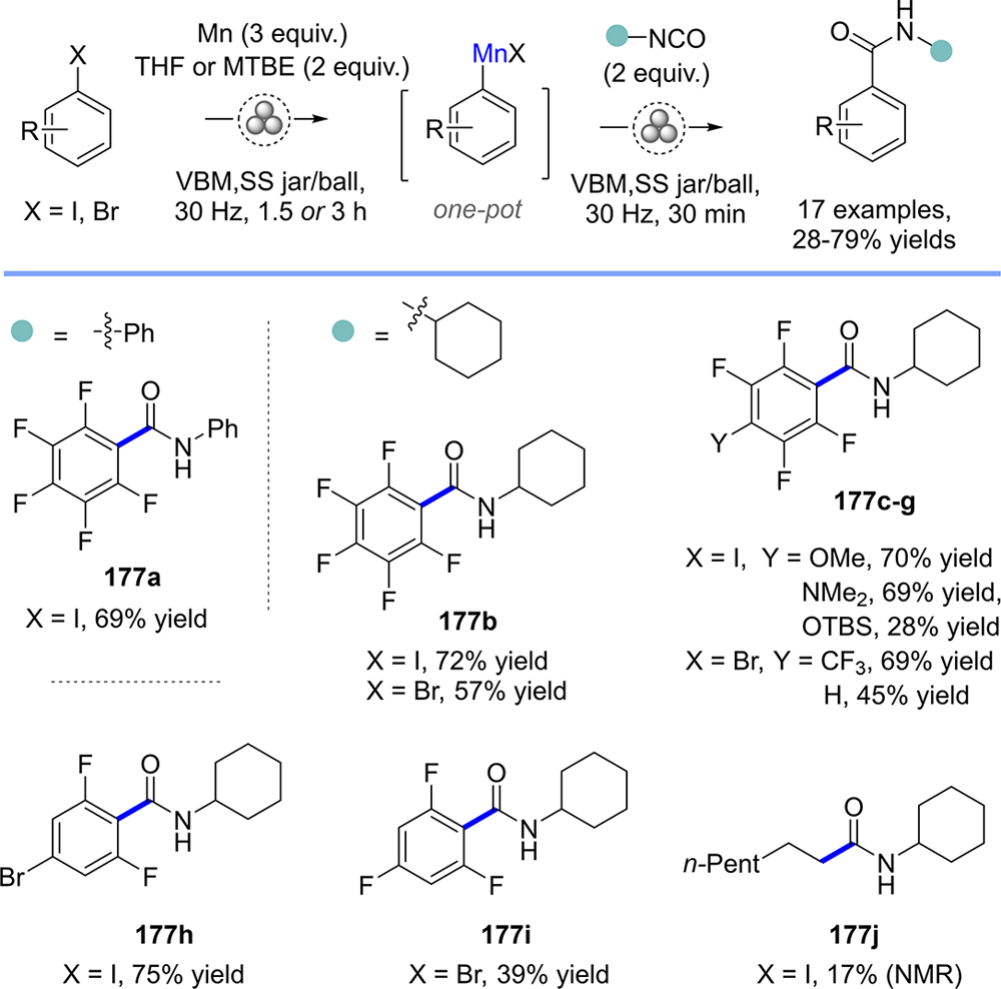
Mechanochemical Synthesis of Amides via the Addition of Arylmanganese Nucleophiles to Isocyanates. Selected Examples

The protocol showed the highest efficiency with (poly)­fluorinated aryl halides, providing the corresponding amides **177a**–**g** in 28–70% yields. Selectivity for aryl iodides over bromides was demonstrated by the selective formation of amide **177h** in 75% yield. Notably, this study also reported the first direct generation of an alkylmanganese nucleophile, leading to amide **177j**, albeit in a low 17% yield.

### 3.3 Redox Transformations

Redox-based transformations provide another nonclassical avenue, enabling access to amides from starting materials in different oxidation states, a direction recently pioneered in mechanochemistry. While C–H functionalization discussed above also involves changes in oxidation state, the methods presented in this section include redox transformations that do not involve transition-metal-mediated activation of C–H bonds. Thus, reported to date mechanochemical methods for the synthesis of amides (where the nitrogen is in oxidation state – 3) utilize either hydroxamic acids (oxidation state – 1)^159^ or nitro compounds (oxidation state +3) as precursors.^160,161^ These transformations generally require catalytic assistance from transition metal or lanthanide-based catalysts. Additionally, formamides can be generated through the reductive amination of carbonyl compounds.^162^

Early work by Wang and Gao^315^ demonstrated the preparation of amides by oxidative amidation of aldehydes with anilines in a mixer mill using Oxone and MgSO_4_.

The use of hydroxamic acids for the synthesis of *N*-aryl amides via mechanochemical adaptations of Ullmann-type (Scheme 57, method A) and Chan-Lam-type (method B) couplings was pioneered by Broumidis et al.^159^

**Scheme 57:**
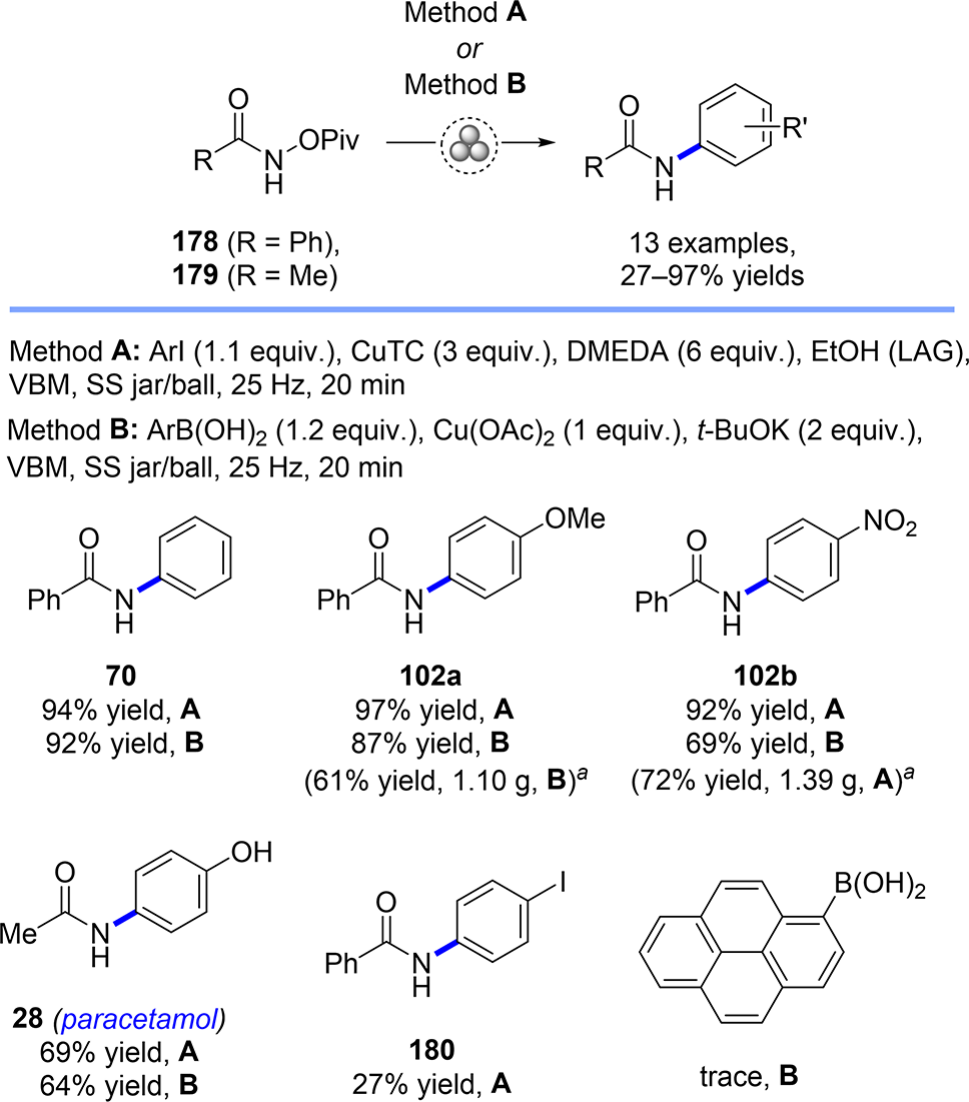
Synthesis of Amides from O-Pivaloylhydroxamic Acids. Selected Examples

a Upscaled reactions were performed in methacrylate milling jars, 20 Hz, 90 min.

This work aimed to address the limitations of analogous solution-phase protocols, which typically require elevated temperatures, toxic solvents (such as DMF or DCM), inert atmosphere conditions, and extended reaction times.

In method A, copper-mediated coupling of aryl iodides with pivaloyl-protected phenyl- and methyl-hydroxamic acids **178** and **179** was achieved under ambient conditions by ball milling in stainless steel reaction vessels. Copper­(I) thiophene-2-carboxylate (CuTC) was used in excess (3 equiv) as the promoter, with *N*,*N*′-dimethylethylenediamine (DMEDA) as the copper ligand. Ethanol served as a crucial liquid-assisted grinding (LAG) additive (*η* = 0.16 μL·mg^–1^), as neat grinding produced amide **70** in a reduced 65% yield. Complementarily, method B was conducted under neat grinding of copper­(II) acetate, hydroxamic acid and aryl boronic acid with potassium *tert*-butoxide. Both approaches afforded rapid reactions (20 min) and consistently higher yields than comparable solution-based processes.

Using these protocols, the synthesis of 13 diverse *N*-aryl amides was demonstrated in yields ranging from 27% to 97%, including 7 examples synthesized via both methods. In addition to amide **70**, representative products included paracetamol (**28**), and benzamides of both electron-rich (*p*-OMe) and electron-poor (*p*-NO_2_) anilines (**102a** and **102b**), all obtained in good to excellent yields. Interestingly, treatment of 1,4-diiodobenzene under method A conditions gave exclusively monoamide **180** in 27% yield, with no trace of diamide detected. Moreover, isolated **180** was found to be unreactive under the same coupling conditions. Pyrene-1-boronic acid afforded a complex reaction mixture containing only trace amounts of the amide product and pyrene, the latter isolated in 13% yield. The formation of pyrene was attributed to protodeboronation occurring under mechanochemical conditions, possibly catalyzed by copper­(II) acetate, similar to the process previously observed in aqueous ethanol solution.^163^

All reactions were conducted in stainless steel jars, and while iron leaching was observed, control experiments confirmed that iron salts had no catalytic influence. PTFE jars were also suitable. Notably, gram-scale syntheses of amides **102a** and **102b** were successfully performed using custom-made, 3D-printed methacrylate milling jars.

Mkrtchyan et al. investigated the use of nitroarenes as precursors for amide synthesis under mechanochemical conditions.^160,161^ In 2021, they reported a unique transformation that combined reductive amidation with C–F bond activation in trifluoromethylarenes **181**, employing ytterbium­(III) oxide as a promoter and elemental silicon as a reducing agent (Scheme 58, A).^160^

**Scheme 58:**
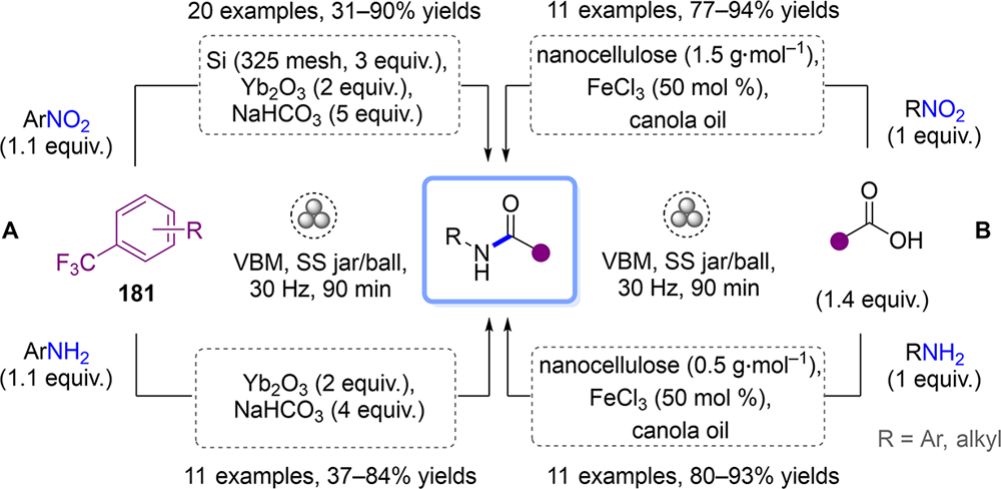
Amide Synthesis from Nitroarenes and Anilines via C–F Bond Activation (A) and Nanocellulose/FeCl_3_-Promoted Reductive Coupling (B)

A total of 20 amides were synthesized from nitroarenes and 11 examples from corresponding amines, with isolated yields ranging from 31% to 90% after 90 min of milling at 30 Hz in a 5 mL stainless steel jar. The methodology exhibited broad substrate scope for trifluoromethylarene derivatives bearing alkyl, aryl, halogen, alkoxy, or 3-pyridyl substituents. However, nitro or amino substrates bearing highly electron-withdrawing groups such as CF_3_, CF_2_H, OCF_3_, or OCF_2_H failed under the same reaction conditions. Additionally, three amide products were successfully synthesized on a 10 mmol scale. In the same study, a related protocol was developed for the synthesis of imines from trifluoromethyl- and nitroarenes, using hexamethyldisilane as the reductant and KF as an additive, further showcasing the versatility of the strategy under mechanochemical conditions.

In 2023, the same group reported the synthesis of aryl and alkyl amides from nitroarenes and carboxylic acids by a FeCl_3_-catalyzed reductive amidation, using nanocellulose as both stoichiometric reducing agent and reaction medium (Scheme 58, B).^161^ In addition, a lubricant (canola oil) was found to be crucial for the reaction. A total of 11 examples that contained both aryl and alkyl moieties were prepared in yields between 77 and 94% (1 mmol scale) after 90 min of milling at 30 Hz in a stainless-steel jar. Concerning the scope of the reaction, nitroarenes bearing halogen, CF_3_ or alkyl moieties were tolerated in this protocol. As carboxylic acids, aromatic (bearing halogen, alkyl or OMe moieties) and alicyclic substrates were successfully used. Furthermore, three examples were scaled up to the 10 mmol scale in 74–91% yield using a 35 mL stainless steel jar. Impressively, the acylation of aromatic amines, including electron-deficient CF_3_-substituted substrates, was accomplished without the need for prior activation of the carboxylic acid. A mechanistic hypothesis was proposed to explain this observation, suggesting FeCl_3_-mediated formation of a carboxylic acid anhydride, which subsequently reacts with the amine to afford the amide. Notable, the reductive amidation process failed to proceed under reflux in various organic solvents even after 24 h, highlighting the clear advantage of mechanochemistry in enabling this transformation.

The synthesis of formamides via the Leuckart reaction using TSE (Scheme 59) was reported by Zorzetto et al.^162^ The developed method operates at an elevated temperature (150 °C), at which ammonium formate decomposes into formic acid and ammonia, with formic acid acting as the active hydride donor. The reaction proceeds remarkably fast, achieving complete conversion of aromatic aldehydes to the corresponding benzylic-type formamides **182** within only 5–15 min of residence time, giving 95–99% yields. The protocol was successfully scaled up for the synthesis of vanillyl formamide (**182a**) on a 25 mmol scale, affording 99% conversion and a space–time yield (STY) of 2.74 kg·L^–1^·h^–1^. However, the reaction scope is limited to solid and thermally stable aromatic aldehydes. Compared with conventional solution-phase protocols, the reactive extrusion approach offers the advantages of significantly shorter reaction times (5–10 min vs hours) and a much lower excess of ammonium formate (3-fold versus up to 100-fold).

**Scheme 59:**
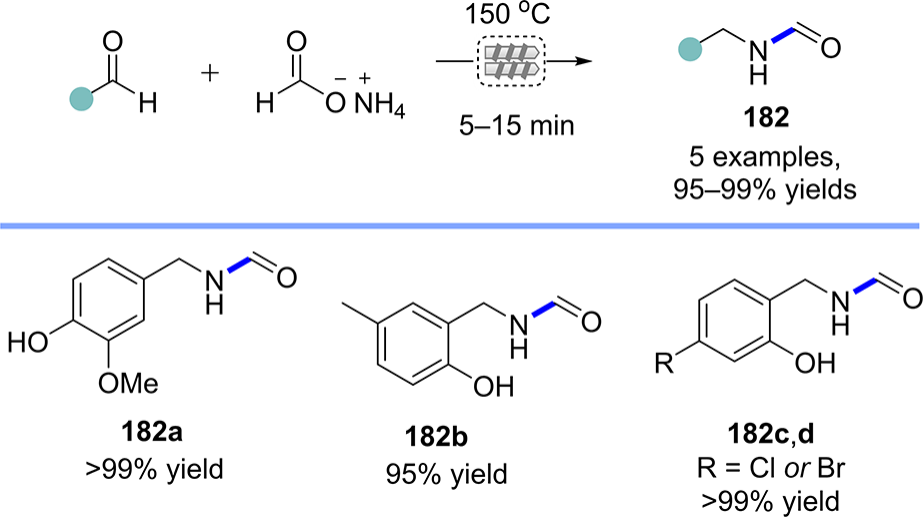
Mechanochemical Synthesis of Formamides via the Leuckart Reaction in a TSE

### 3.4 Rearrangements and Multicomponent Reactions

Beyond the established classical and nonclassical mechanochemical amidation pathways discussed previously, the field has witnessed the implementation of several other strategies. These miscellaneous approaches, encompassing molecular rearrangements and multicomponent reactions (MCRs) further underscore the expanding scope and versatility of mechanochemistry for constructing amide bonds, often leveraging unique reactivity inherent to the solid state or intensified conditions.

#### 3.4.1 Beckmann Rearrangement

Rearrangement reactions are particularly valuable in synthetic chemistry as they occur with inherent 100% atom economy. Among the mechanochemical rearrangements,^316,317^ the Beckmann rearrangement, a classical transformation converting ketoximes into secondary amides, is especially significant. It is widely used in industry for the synthesis of analgesic paracetamol and ε-caprolactam (a precursor to nylon-6,6). Typically, this rearrangement requires an activating reagent and/or a Brønsted acid catalyst to proceed.

In 2021, Mocci et al.^164^ reported the mechanochemical adaptation of the Beckmann rearrangement, performed entirely under solvent-free conditions (Scheme 60). Through screening of acids and activating agents, *p*-toluenesulfonyl imidazole (*p*-Ts-Im), combined with either *p*-toluenesulfonic (*p*-TsOH) or oxalic acid, were identified as the optimal reagent systems. Under these conditions, acetophenone oxime **183** was nearly quantitatively converted into acetanilide **184**. The reaction was rapid, completing in just 15 min when using oxalic acid. The same mechanochemical protocol enabled rearrangement of other oximes, including the synthesis of ε-caprolactam **185** in 93% yield.

**Scheme 60:**
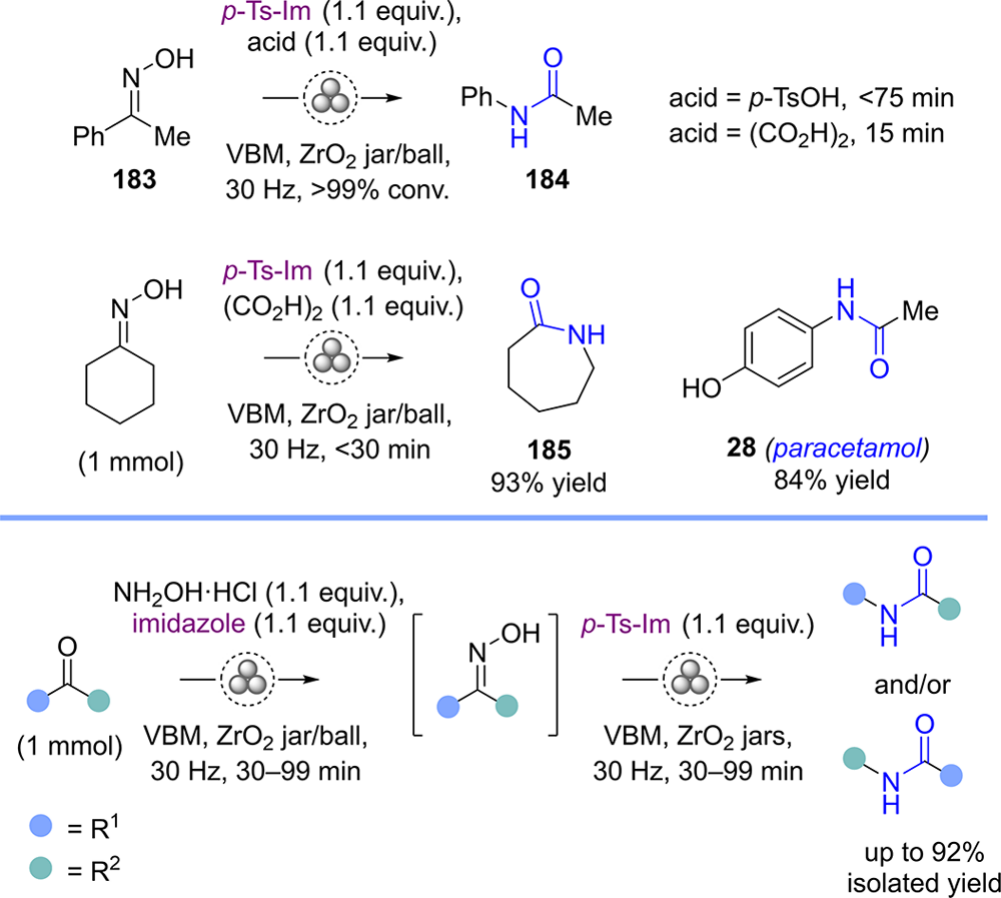
Mechanochemical Beckmann Rearrangement Promoted by *p*-Toluenesulfonyl Imidazole. Selected Examples

Importantly, the methodology was also successfully adapted to a one-pot, two-step procedure directly from ketones, involving initial *in situ* oxime formation followed by rearrangement to the amide. A total of 32 ketones were transformed to the corresponding amides in up to 92% yields, including the synthesis of the pharmaceutically relevant compound paracetamol (**28**) in 84% yield. Among 26 examples of unsymmetrical ketones (R^1^ ≠ R^2^) tested, 11 substrates yielded mixtures of isomeric amides in varying ratios, as the migration selectivity depends on the *E*/*Z* configuration of the oxime and the nature of the R^1^ and R^2^ substituents.

This mechanochemical adaptation presents notable advantages compared to classical Beckmann rearrangement protocols, which generally employ strong acids and harsh conditions. Furthermore, the reported mechanochemical conditions demonstrated superior green chemistry metrics compared to benchmark solution-based processes.

In 2022, Baier et al.^165^ reported an alternative methodology for the Beckmann rearrangement, adapted to both planetary ball milling and continuous processing in a twin-screw extruder. Following a comprehensive screening of reaction parameters, various acids, and additives, the optimal system was identified as a combination of SiO_2_ and Al_2_O_3_ nanoparticles (AlSi NP), phosphorus pentoxide, and *p*-toluenesulfonic acid (Scheme 61). This formulation enabled the synthesis of the model compounds ε-caprolactam **185** and acetanilide **184** in 46% and 94% yield, respectively. Five additional amides were synthesized in the planetary ball mill, affording moderate to good yields, except for paracetamol (**28**), which was obtained in only 4% yield due to sulfonylation of the phenolic OH group by *p*-TsOH. The methodology was successfully translated to twin-screw extrusion by employing montmorillonite clay (M10) as an aluminosilicate additive and increasing the processing temperature above 100 °C. Under these conditions, yields ranging from 44% to 90% were achieved with residence times as short as 7 min, including the preparation of paracetamol in an improved 56% yield. Raising the temperature to 150 °C increased the yield of **185** from 44% to 56%, but reduced the yield of **184** from 90% to 78%. This effect was attributed to evaporation or thermal decomposition of the starting oxime. Although the conversion to **185** improved, decomposition of other reaction components was observed, and therefore the temperature was not increased further. A consistent trend in product yields was observed across both planetary milling and extrusion, with generally comparable results. In the case of twin-screw extrusion, an inverse correlation was noted between product yield and the melting points of the substrates and products. Higher melting points were associated with lower yields, likely due to unfavorable rheological properties of the reaction mixture that hinder effective mixing.

**Scheme 61:**
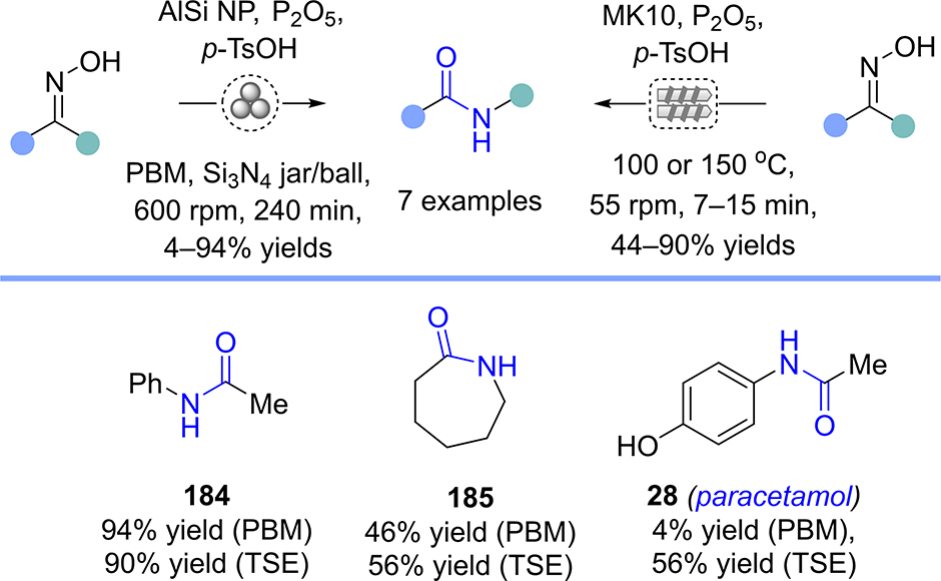
Mechanochemical Beckmann Rearrangement Promoted by Solid Acids

In 2024, Geib et al.^166^ presented the first application of bead mill technology for the mechanochemical synthesis of acetanilide (**184**) and paracetamol (**28**) *via* the Beckmann rearrangement (Scheme 62). Using a Dyno-mill instrument, the method scaled up a previously established protocol^164^ employing *p*-toluenesulfonyl imidazole in combination with oxalic acid as the acidic promoter. High-speed milling was conducted with 0.5 mm stabilized zirconia/yttria beads, with a 50% (v/v) filling degree of the milling chamber. Following optimization, the protocol enabled the synthesis of both amides on a nearly decagram scale, affording high isolated yields and product purity. The presence of abraded materials was assessed by Microwave Plasma-Atomic Emission Spectrometry (MP-AES), revealing low levels of yttrium (1.1 ppm) and zirconium (5.3 ppm). Overall, the optimized processes demonstrated superior green chemistry metrics compared to a benchmarking solution-phase method.

**Scheme 62:**
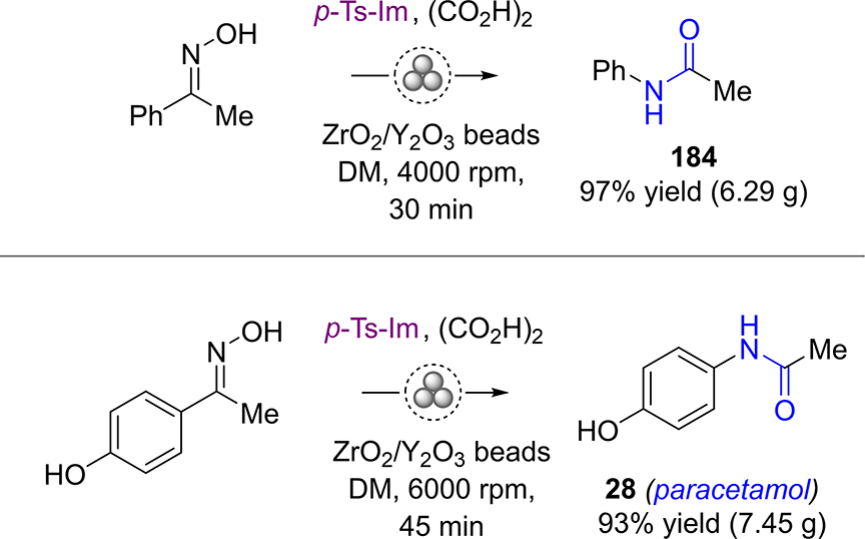
Upscaled Synthesis of Paracetamol and Acetanilide in a Dyno®-Mill

#### 3.4.2 Multicomponent Reactions (MCRs)

Multicomponent reactions (MCRs) are inherently well-suited to mechanochemical methodologies, as they benefit from the high concentrations and solvent-free conditions typical of ball milling. These reactions enable the efficient construction of complex amide-containing molecules in a single step, aligning well with the principles of green and sustainable synthesis.

Polindara-García and Juaristi^167^ were the first to adapt the classical Ugi four-component reaction (involving an aldehyde, amine, carboxylic acid, and isocyanide) to high-speed ball-milling conditions (45 min, 25 Hz, agate vessel). Optimal results were obtained using methanol as a LAG additive and indium­(III) chloride (2 mol %) as a catalyst (Scheme 63, A). Under these conditions, Ugi adducts **186** were obtained in 46–74% yields from 16 different aldehydes (predominantly benzaldehyde derivatives, 1-naphthaldehyde, the heteroaromatic pyrrole-2-carbaldehyde, and a single example of the aliphatic cyclohexanecarbaldehyde), *tert*-butyl isocyanide, chloroacetic acid, and propargylamine. A gram-scale reaction product (**186a**) was prepared in 55% yield. Subsequently, a number of BODIPY-containing Ugi adducts (e.g., **186b**) were synthesized by the same method in 14–98% yields, from propargylamine and various anilines (6 examples), carboxylic acids (5 examples) and BODIPY-containing aldehydes (4 examples).^168^ With anilines, the reaction proceeded even without the InCl_3_ catalyst and regardless of the nature of substituents (including *o*- and *p*–OH, *p*-NO_2_, *p*-Me, *p*-Cl, and *p*-Br), whereas Ugi adducts were not obtained from α-methylbenzylamine and *o*-nitroaniline. The reaction was complete in 90 min at room temperature, rather than after 3 h of heating at 50 °C needed to attain similar yields in solution synthesis.

**Scheme 63:**
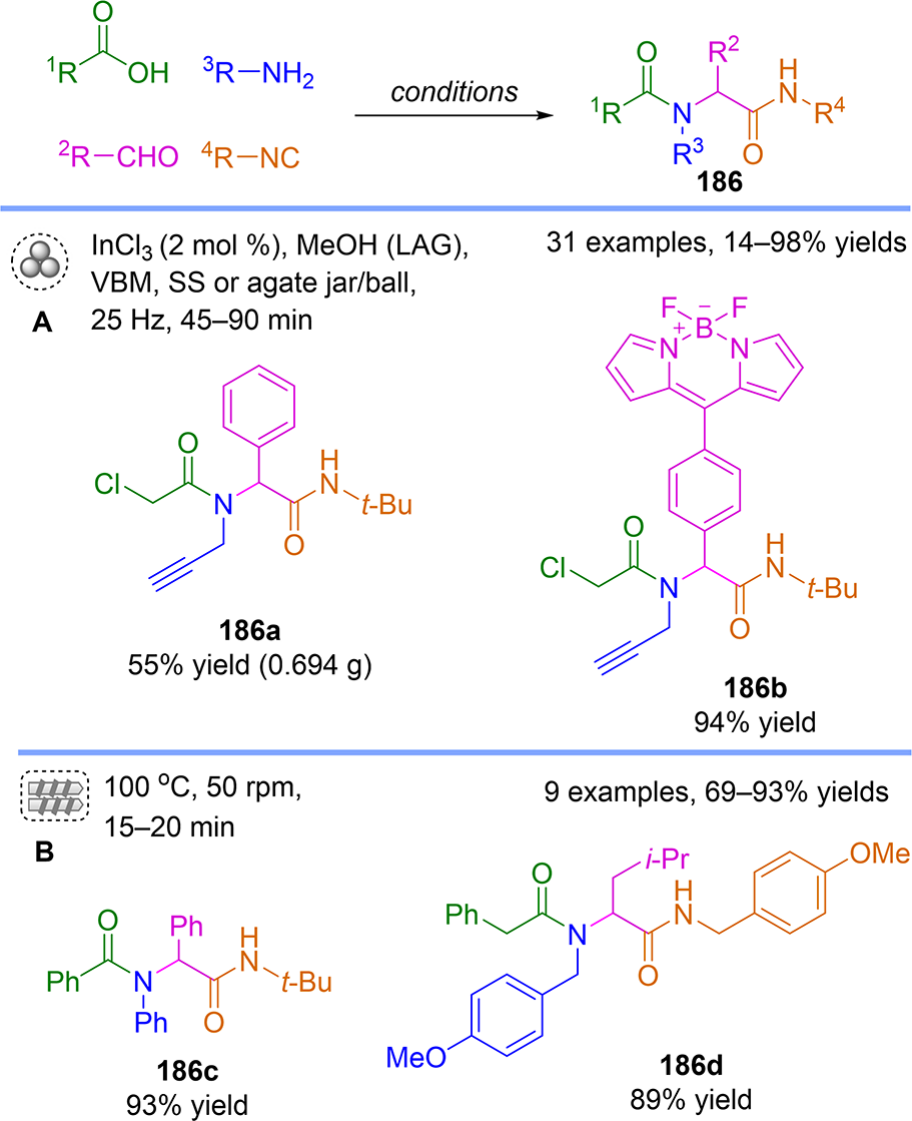
Mechanochemical Ugi Reaction

El-Remaily et al.^169^ further advanced the Ugi reaction by implementing it under solvent-free conditions via twin-screw extrusion (Scheme 63, B). This continuous processing technique delivered α-acylamino amides **186** in up to 93% yield with short residence times (15–20 min) at 100 °C, demonstrating the suitability of TSE for scalable MCR-based amide synthesis. The nature of the substituents in the starting materials had a rather insignificant effect on the product yields (e.g., **186c** and **186d**).

Polindara-García and Juaristi^167^ also reported the first mechanochemical adaptation of the Passerini three-component reaction (aromatic aldehydes, carboxylic acids, and isocyanides, Scheme 64), affording 12 examples of α-acyloxy amides **187** in 40–92% yield by neat grinding for 90 min in a vibratory ball mill with an agate milling vessel. The substrate scope included predominantly benzoic acids and its *p*-chloro- and *p*-nitro-substituted derivatives, benzaldehyde and its *p*-substituted derivatives, *tert*-butyl and cyclohexyl isocyanides. A single example using the aliphatic 3-bromopropionic acid delivered adduct **187a** in 46% yield. Gram-scale preparation of the Passerini adduct **187b** was also demonstrated.

**Scheme 64:**
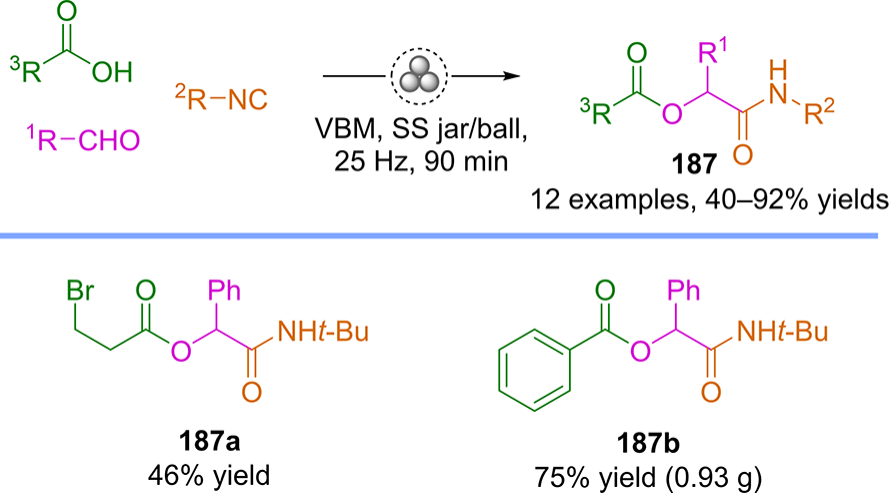
Mechanochemical Passerini Reaction

Additional work by Salami et al. applied the Passerini reaction under ball-milling conditions using silica-supported sulfuric acid as a solid promoter. This protocol afforded α-acyloxycarboxamides in excellent yields (up to 95%) within 15–30 min.^170^

In summary, these studies highlight the utility of mechanochemistry in enabling complex bond-forming cascades under solvent-free conditions. Ugi and Passerini reactions in particular underscore the potential of this approach for the efficient synthesis of functionalized amide scaffolds beyond conventional methods.

## 4 Mechanoenzymatic Transformations

After presenting a comprehensive array of nonenzymatic methods for mechanochemical amidation, this section shifts the focus toward mechanoenzymology, an important intersection of mechanochemistry and biocatalysis. Mechanoenzymology has recently emerged as a promising subfield in mechanochemistry, demonstrating successful application of enzymes to mediate diverse chemical transformations within shaker mills, planetary ball mills, and twin-screw extruders.^171^ The achievements and challenges in this field have been summarized in several review articles,^33,171–174^ one of which focuses on green chemistry aspects.^175^ Since this review focuses on mechanochemical amidation, the following discussion centers on enzyme-mediated formation and cleavage of amide and peptide bonds, with other transformations mentioned only when essential to the discussion.

The efficiency of mechanoenzymatic methods was first demonstrated in the kinetic resolution of secondary alcohols,^176^ β-amino acids,^177^ and amines^178^ using lipase B from *Candida antactica* (CALB), and was later extended to a range of other synthetic applications.^173^ In brief, mechanoenzymatic protocols typically exhibit faster reaction rates, higher space-time yields and lower energy consumption^175^ compared to their solution-based counterparts, while preserving high enzyme selectivity (e.g., enantioselectivity above 99% *ee* in the kinetic resolution processes). They also circumvent solubility limitations while providing efficient mixing in highly viscous media, maintaining high reaction rates without causing enzyme inactivation due to elevated concentrations or water-depleted conditions.^173^ Notably, the activity of glycosyl hydrolases has even been found to increase in the absence of bulk water.^172^ Avoiding bulk solvent helps to reduce the PMI values of biocatalytic processes, which might otherwise be relatively high,^179^ thereby enhancing the overall sustainability and practicality of biocatalytic synthesis. Thus, LAG and reactive aging (RAging),^180^ two common mechanoenzymatic strategies, operate at typical liquid-to-solid ratios below 2 μL·mg^–1^ and 1 μL·mg^–1^, respectively, closely resembling the moisture conditions found in the natural habitats of enzymes.^173^

In view of the high cost and limited commercial availability of many enzymes, their reusability and resistance to degradation, and thus to the loss of catalytic activity, are of particular importance. As demonstrated in preceding sections, short peptide fragments can be efficiently synthesized via mechanochemical nonenzymatic methods, highlighting their resistance to chemical degradation despite the mechanical stresses induced by grinding. However, harsh reaction conditions can disrupt the tertiary or quaternary structure of enzymes, leading to partial or complete unfolding (denaturation) and consequently to a loss of catalytic activity due to disruption of the enzyme’s active site. Remarkably, a number of enzymatic catalysts have shown robustness under mechanochemical conditions, retaining high catalytic activity and selectivity, which enables their recovery and reuse. However, such resilience varies with the enzyme, its formulation, and the process parameters, and activity may decline after several reaction cycles.

For instance, CALB immobilized on acrylic resin was recovered and reused up to four times, allowing for acylative kinetic resolution of secondary alcohols in a ball mill.^176^ In the resolution of racemic 1-phenylethanol, excellent enantioselectivity (>99% *ee*) was achieved in each cycle, indicating that active center was unaffected. However, gradually reduced conversions were observed, which was ascribed to leaching of the enzyme from the solid support upon milling. Interestingly, the catalyst premilled in a VBM (90 min, 25 Hz, ZrO_2_ milling jar and ball) was totally inactive in hexane solution, indicating on plausible denaturation. Later, Hammerer et al. demonstrated that chemically inert additives preserve catalytic activity of β-glucosidases during ball milling, preventing denaturation.^180^

A systematic study of thermal and mechanical stability of CALB immobilized on acrylic resin (Novozym 435, N435) was undertaken by Pérez-Venegas et al.^181^ Remarkably, milling of the catalyst using agate milling jar and ball for 90 min in a VBM increased the enzymatic activity in 1 order of magnitude, without loss of stereoselectivity. This effect was attributed to the reduced particle size, which increases the effective concentration of N435 and facilitates substrate diffusion. At the same time, no enzyme leakage from the solid support was detected. Furthermore, examination of the enzyme kinetics in the resolution of racemic 1-phenylethanol ruled out plausible thermal deactivation and indicated no structural damage to the biocatalyst as a result of mechanical stress, at least under moderate frequencies (10–25 Hz), short reaction times (up to 90 min), and irrespective of material hardness (agate or Teflon). The observed decrease in enzymatic activity and loss of enantiopreference after milling were therefore attributed to a different mode of inactivation, likely caused by contact with organic solvents during enzyme recovery. However, unlike CALB, *Candida antarctica* lipase A (CALA) exhibited markedly different behavior, showing reduced activity and enantioselectivity under mechanochemical conditions, likely due to denaturation caused by a different support matrix (Immobead 150). In addition, porcine pancreatic lipase (PPL), which is inactive in solution, displayed significant but nonenantioselective conversion, indicating that mechanical force alone can induce biocatalytic activity.

Consequently, these findings highlight the need for a deeper understanding of the structural and functional alterations experienced by the enzymes during milling and subsequent recovery cycles. In addition, further research is required to improve enzyme recyclability and durability, and to broaden the scope of transformations and biocatalysts suitable for mechanochemical applications.

In the context of amide bond formation, CALB-mediated acylation was applied to achieve kinetic resolution of racemic amines (Scheme 65).^178^ In the initial experiment, racemic 1-phenylethan-1-amine (0.8 mmol) was milled with ethyl acetate (6 equiv) and CALB (100 mg) for 90 min at 25 Hz in an agate jar, affording the acylated product **188a** in 32% yield and excellent enantiopurity (>99% *ee*). The yield increased to 40% (theoretical maximum: 50%) upon addition of dioxane as a LAG additive. The choice of milling materials significantly affected the outcome: yields dropped with stainless steel jars and no reaction occurred in PMMA vessels. The optimized protocol was applied to a representative set of nine racemic amines, affording the corresponding acetamides **188** in 20–48% yields and with enantioselectivities ranging from 66% to >99% *ee*, except for acetamide **188c**, which was obtained in racemic form. The substrate scope mirrored that of solution-based approaches, yet reaction times were dramatically reduced, from hours or even days in solution to just 90 min under mechanochemical conditions. Notably, in the case of arylamines, the mechanoenzymatic method gave higher enantiopurities than conventional solution-phase reactions. The reactions performed in dioxane solution between 55 and 80 °C showed lower conversions, enantiomeric excess and delivered several byproducts. The biocatalyst could be recovered and reused without loss of enantioselectivity, although the yield of **188a** decreased from 40% to 30% after the first recovery. The yield continued to decline with each subsequent cycle, leading to complete enzyme deactivation after the third use. To demonstrate synthetic utility, both (*S*)- and (*R*)-Rasagilines **191** were prepared from the racemic amine precursor **189** with excellent yields and enantiopurity (>99% *ee*, Scheme 65). The (*R*)-enantiomer is clinically used in the treatment of Parkinson’s disease. Later, the performance of ground N435 was reinvestigated in the kinetic resolution of 1-(2-naphthyl)­ethylamine, demonstrating ca. 25% faster biocatalysis and higher enantiopreference than with the untreated enzyme.^181^

**Scheme 65:**
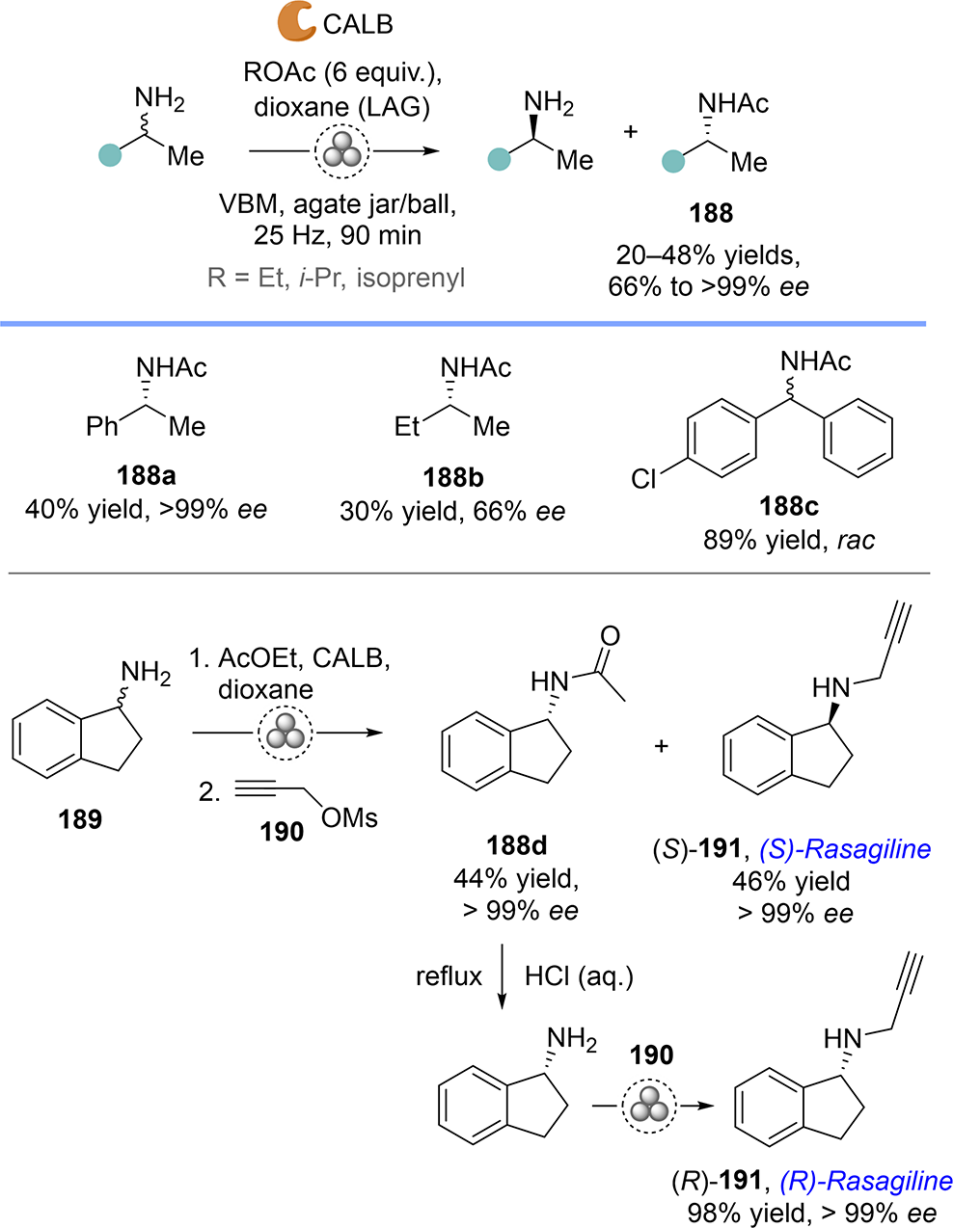
CALB-Mediated Mechanoenzymatic Kinetic Resolution of Racemic Secondary Amines, Selected Examples

The application of enzymes under mechanochemical conditions to mediate the formation of peptide bonds represents an appealing alternative to conventional coupling methods, as enzymatic approach allows the avoidance of coupling reagents while preserving stereochemical integrity. The application of proteases for mechanoenzymatic peptide synthesis was first developed by Hernández et al.^182^ Although proteases primarily catalyze the hydrolysis of peptide bonds, they can also facilitate the formation of amide bonds from amino acids and amino esters. Using peptide hydrolase EC 3.4.22.2 (papain), a range of dipeptides was prepared from *N*-protected amino esters, HCl salts of α-amino acid amides and β-alanine in a VBM (Scheme 66). l-Cysteine was added as an activating additive for papain, resulting in enhanced product yields, while Na_2_CO_3_ was introduced as a hydrated base. Substituting the anhydrous salt with its monohydrate increased the yield of dipeptide **192a** from 63% to 82%, and the use of the decahydrate further improved it to 90%. These findings highlight the crucial role of water molecules, which are proposed to stabilize the enzyme’s active conformation and promote more effective mixing of the reactants. The presence of crystal water likely facilitates the formation of a more homogeneous reaction medium during milling, an effect that appears to outweigh water’s tendency to drive the reaction toward hydrolysis.

**Scheme 66:**
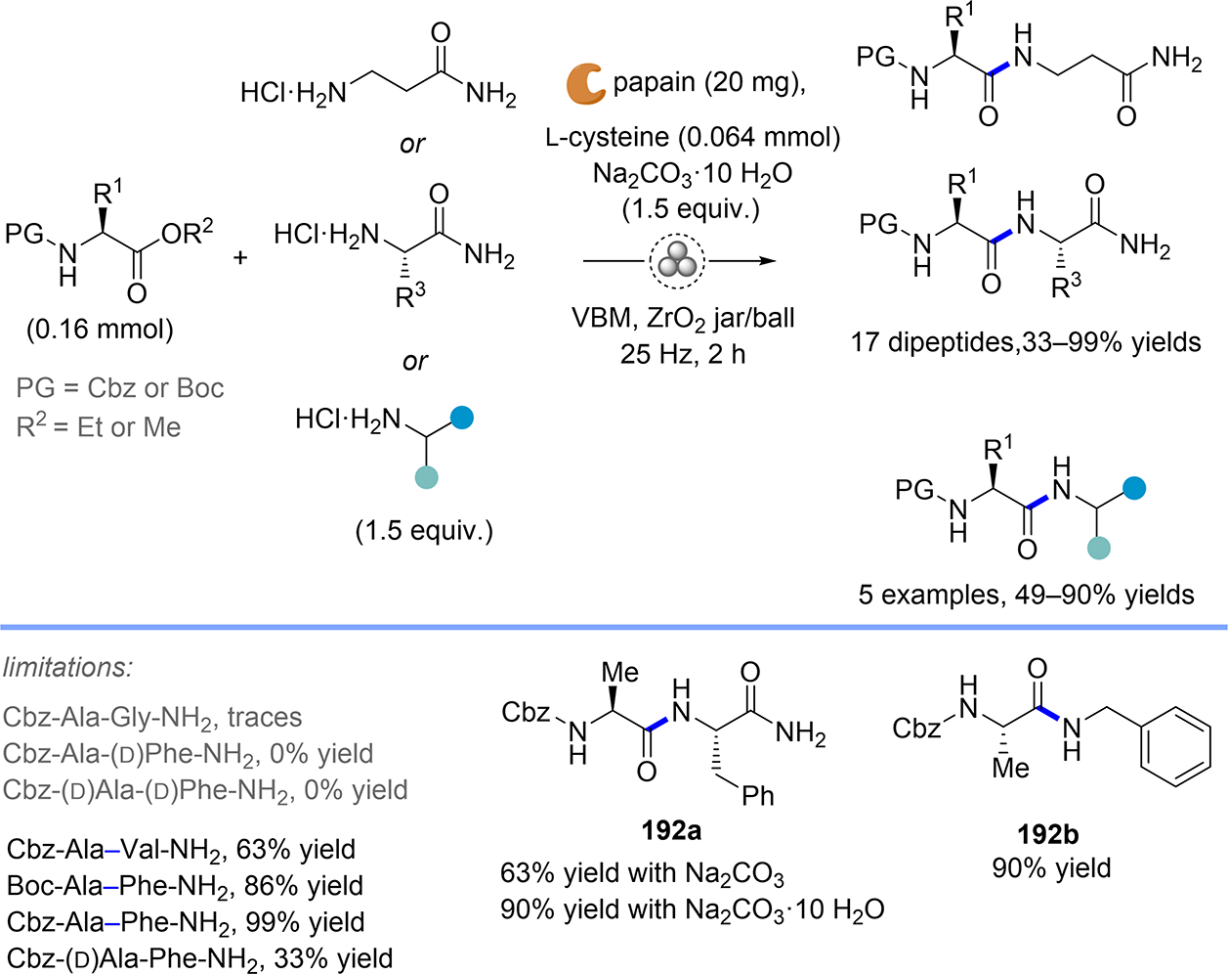
Mechanoenzymatic Formation of Peptide and Amide Bonds Catalyzed by Papain

The use of the Cbz-group as an *N*-protecting group at the terminal position resulted in better outcomes compared to Boc protection. While papain exhibited broad tolerance toward various R^1^ substituents in amino acid esters (AA1), it showed a clear preference for bulky or aromatic hydrophobic R^3^ substituents in the coupling partner (AA2), such as Leu, Ile, or Phe. Although the enzyme was capable of incorporating d-AA1 into dipeptides, the yields were lower than those obtained with the corresponding l-enantiomers. Notably, no product formation was observed when d-AA2 was used, suggesting that papain is more sensitive to the configuration of the AA2 component. In addition to amino acid couplings, standard amide bond formation was also demonstrated using benzylic amines, exemplified by the synthesis of amide **192b**.

The procedure with papain was further extended to the formation of homo-oligopeptides (Scheme 67) by both ball milling and twin-screw extrusion. Examples of produced homo-oligopeptides include oligo-Phe, -Leu, -Ala and -Gly, with degrees of polymerization varying between 6 to 26.^183^ Oligomerization of the respective methyl esters produced homopolypeptides whose degree of polymerization (DP) could be tuned by adjusting the milling time and water content, controlled through the use of anhydrous or hydrated Na_2_CO_3_. Anhydrous conditions (Na_2_CO_3_) favored higher yields for the more hydrophilic Ala and Gly, while additional water in decahydrate increased yields for hydrophobic Phe and Leu esters. In all cases, the presence of water promoted the formation of longer oligomers (DP > 8) compared with dry conditions, with polymer length inversely correlated to monomer hydrophobicity and steric bulk. The process was successfully scaled up to a 10 g of methyl ester load using TSE. The results mirrored those from ball milling, with hydrophilic amino acid esters giving lower conversions and the degree of polymerization decreasing in the order Ala > Leu > Phe. The reaction PMI for oligo-Leu synthesis was approximately four times lower in the ball mill and three times lower using the TSE method compared to the ring-opening polymerization of leucine NCA in THF solution. The synthesized oligopeptides were evaluated as catalysts in the asymmetric Julia–Colonna epoxidation of chalcones, with oligo-Leu exhibiting the best performance.

**Scheme 67:**
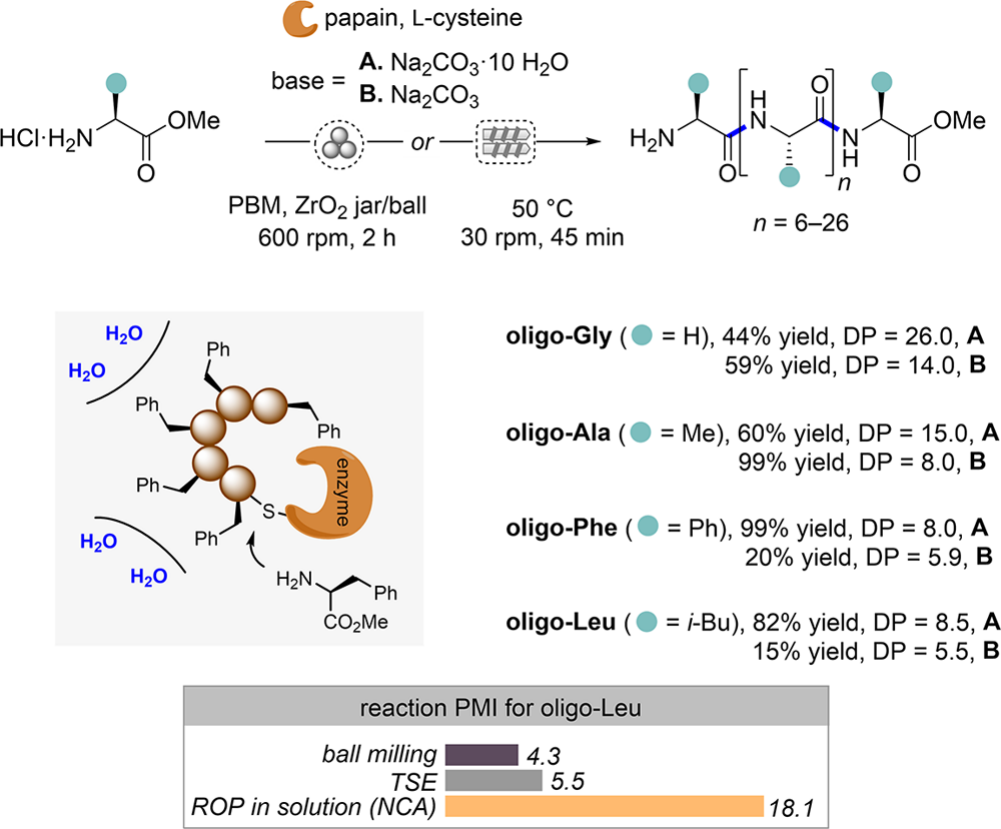
Mechanoenzymatic Synthesis of Homo-Oligopeptides and Cartoon Depiction of a Hydrophobic Pocket in the Growing oligo-Phe^a^

a Reproduced with Permission from reference 183. Copyright 2018, Royal Society of Chemistry.

The high concentration of reactants in the absence of bulk solvent likely contributes to the efficiency of oligomerization under ball-milling conditions, as comparable or longer reaction times in solution (up to 24 h) are required to obtain the same peptides, typically in lower yields. The DPs of the obtained oligopeptides were comparable to those achieved in aqueous or cosolvent systems and followed the same intrinsic reactivity trend (Gly > Ala > Leu > Phe) observed in solution chemistry, even though solid-state conditions circumvent the premature precipitation that limits DP in solution.

These results imply that enzyme–substrate interactions and steric factors could play a key role in controlling the oligomerization process. The presence of water appears to promote the formation of hydrophobic pockets that enhance the proximity between lipophilic amino acid monomers (Leu and Phe esters) and the enzyme’s active site (Scheme 67), whereas the oligomerization of the less hydrophobic Ala and Gly esters is less affected by water.

In 2025, the combination of chemical and mechanoenzymatic methodologies was presented for the synthesis of l-Pro- and l-Phe-containing oligopeptides (Scheme 68).^184^

**Scheme 68:**
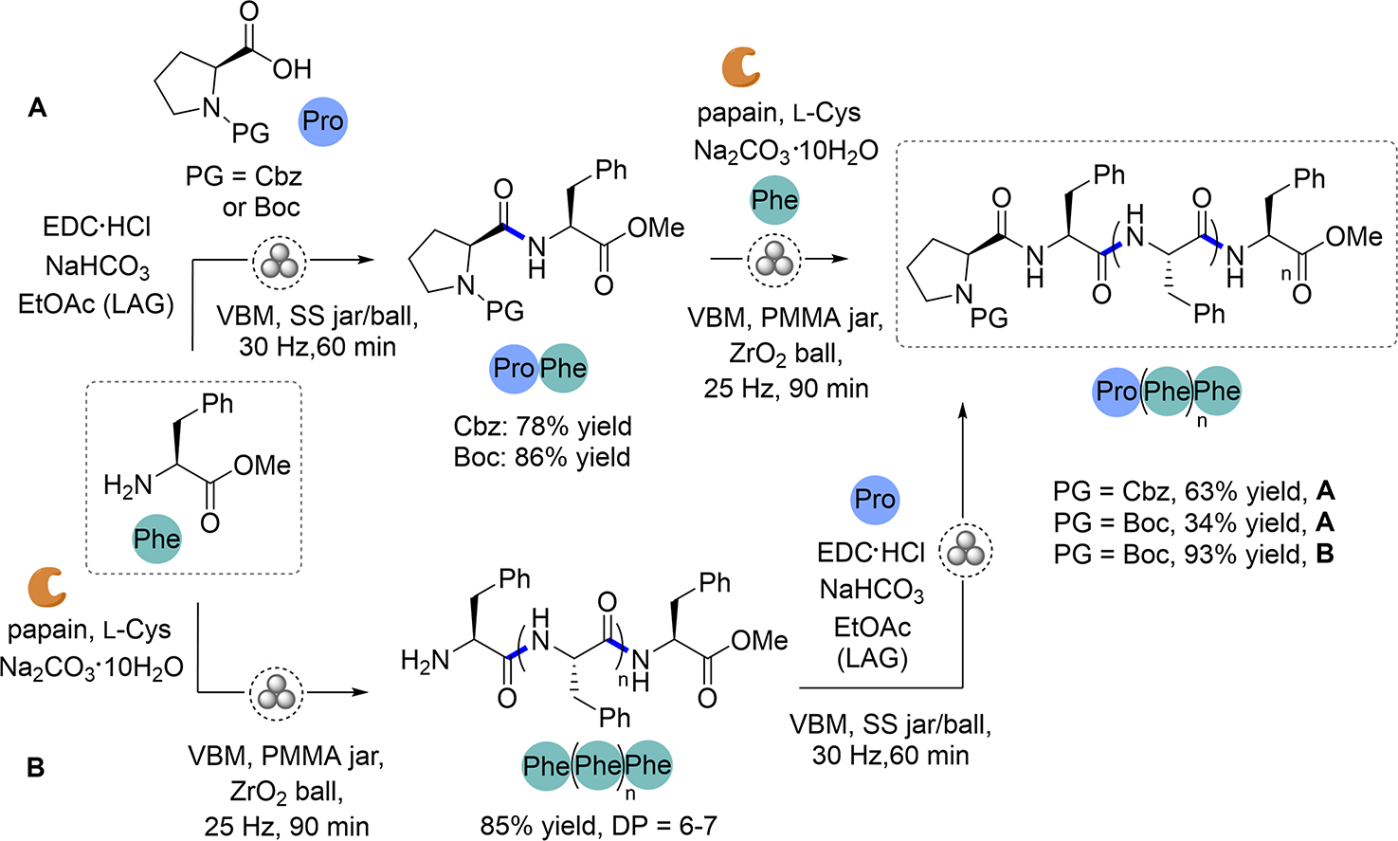
Chemical and Enzymatic Mechanosynthesis of Organocatalytic Oligopeptides

The first synthetic route (A) involved an initial EDC-mediated coupling of Cbz or Boc-protected l-proline with methyl ester of l-phenylalanine, followed by enzymatic oligomerization to extend the phenylalanine segment. However, this approach yielded only moderate amounts of the desired Boc- and Cbz-protected heteropeptides (34% and 63% yields, respectively), along with oligo-Phe byproducts. In contrast, first synthesizing oligo-Phe via the enzymatic method and then coupling the product with Boc-protected l-proline using EDC (route B) resulted in a significantly improved yield of 93%. Compared to solution-based protocols, the mechanochemical method offers notable advantages, including fewer synthetic steps, reduced reaction times, and higher yields. Furthermore, the synthesized peptides demonstrated organocatalytic activity in the asymmetric aldol reaction between cyclohexanone and 4-nitrobenzaldehyde.

The studies presented above demonstrate the feasibility of forming peptide and amide bonds without bulk solvent, using proteases as biocatalysts. Operating in the solid state provides a practical means to obtain dipeptide and oligopeptide products in high yields while minimizing undesired back-hydrolysis. However, the successful examples reported so far have mainly involved amino acid esters with hydrophobic side chains, and further research is required to extend this approach to a broader range of residues from both natural and non-natural amino acids.

## 5 Stereochemical Aspects of Mechanochemical Amide Synthesis

Mechanochemical approaches to the synthesis of target compounds can lead to alterations in reaction mechanisms, selectivity, and outcomes compared to traditional solvent-based methods. This also applies to the stereochemical aspects of these transformations, where asymmetric induction or the preservation of stereocenters may differ significantly from conventional approaches.

Maintaining stereochemical integrity is crucial in the synthesis of APIs and represents a well-known challenge in peptide synthesis. In the context of mechanochemical amidation, peptide bond formation from amino acids serves as a representative model for evaluating the extent of stereochemical preservation. This allows direct comparison with established methods such as solid-phase peptide synthesis (SPPS) and solution-phase techniques. Particularly α-stereocenters of amino acids may undergo partial epimerization through different mechanisms, such as base-catalyzed direct enolization or oxazolone formation (Scheme 69).

**Scheme 69:**
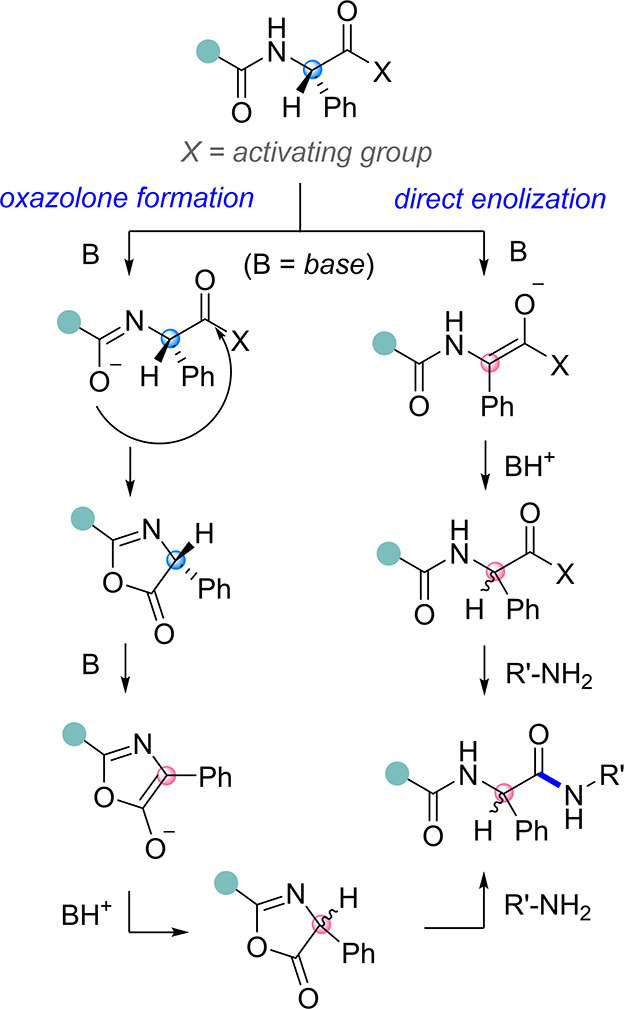
Mechanistic Pathways Leading to Epimerization of α-AA Stereocenters During Peptide Synthesis

In 2009, Declerck et al.^59^ presented the first mechanochemical approach for the synthesis of di- and tripeptides through coupling of α-amino acid UNCA derivatives with an amino ester (Scheme 5). No racemization was detected during the synthesis of Boc-(D)­Phe-(L)­Ala-OMe.^59^ From 2009 onward, several groups reported versatile mechanochemical methods applied to peptide synthesis (see section 2). Di-, tri- and oligopeptides have been predominantly prepared using versatile coupling reagents for activation of carboxylic acids^62,80,81,84,85,92,93,96,97,99,102,107,110^ or through preactivated derivatives, such as *N*-carboxyanhydrides^59,60,63,64^ or acyl fluorides.^86^

In general, applying these approaches in a mechanochemical setting using amino acids containing an α-stereocenter do not lead to significant racemization or epimerization, demonstrating excellent preservation of chirality regardless of the method used (ball milling,^59,60,63,64,80,81,84,86,92,93,96,99,102^ twin-screw extrusion,^62,107^ or manual grinding^85^).

With the aim to compare the epimerization rate on between mechanochemical and traditional solvent-based methods, a systematic study was performed by Yeboue et al.^97^ A model tripeptide was synthesized by coupling of Cbz-Ala-Phg–OH and HCl·NH_2_–Ile-OMe, using a series of different coupling reagents both in solution as well as in a vibratory ball mill with DMF as a solvent (Scheme 70). Phenylglycine (Phg) was chosen due to its tendency to epimerize, while isoleucine (Ile) is a branched amino acid with a relative slow coupling rate. The study demonstrated that ball-milling outperforms solution-based peptide coupling with regards to reaction time, chemical purity as well as level of epimerization, while achieving similar yields (Table 9). However, the results highlight that undesired epimerization remains a threat even when using mechanochemistry, since formation of the LDL epimer (see Table 9) occurred for some frequently used coupling reagents, albeit at a lower level compared to the solution-based reactions. Among the coupling reagents, EDC·HCl/Oxyma with ethyl acetate as a LAG additive (*η* = 0.9 μL·mg^–1^) showed the least epimerization, allowing to synthesize 16 tripeptides with excellent diastereomeric excess (98–99%), as determined by cHPLC.^97^ The low level of epimerization was attributed to the high concentration of the reaction mixture, which decreases the probability of intramolecular oxazolone formation and promotes faster peptide coupling. However, beyond this kinetic suppression, other factors may also contribute. Epimerization is known to proceed more readily in protonic and polar solvents, whereas reactions in a ball mill likely occur within an amorphous phase composed of reactants, products, and additives. This ‘liquid-like’ environment may provide less stabilization for racemization intermediates than conventional solvents, thereby contributing to the reduced extent of epimerization. Further studies are nevertheless required to elucidate the role of the reaction medium and the specific mechanisms responsible for stereochemical preservation.

**Scheme 70:**
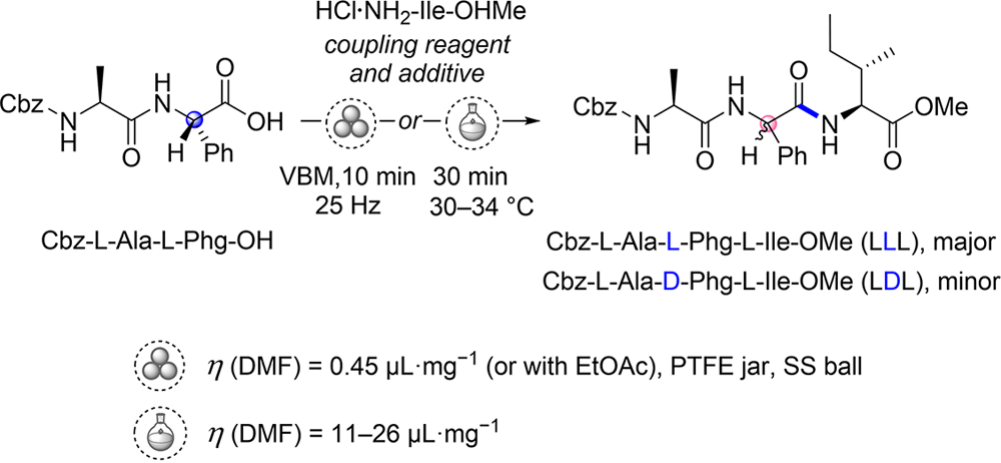
Model Peptide Synthesis to Evaluate Coupling-Induced Epimerization

**Table 9:** Model Peptide Synthesis to Evaluate Coupling-Induced Epimerization: Ball Milling *vs* Solution Synthesis^a^

Coupling reagents	Time (min)	Yield (%)	Purity (%)	LDL (%)^b^
EDC·HCl/Oxyma	10 (30)	93 (88)	>99 (32)	<1 (9)
EDC·HCl/HOBt·H_2_O	10 (30)	90 (90)	70 (48)	25 (35)
EDC·HCl/HOAt	10 (30)	88 (90)	95 (59)	<1 (26)
DIC/HOAt	10 (40)	n.d. (n.d.)	67 (39)	17 (33)
DIC/Oxyma	10 (40)	n.d. (n.d.)	46 (<10)	<1 (n.d.)
HATU/Et_3_N	10 (60)	85 (88)	88 (58)	1 (<1)
HBTU/Et_3_N	10 (20)	86 (82)	71 (55)	2 (9)
EDC·HCl/Oxyma^c^	30	96	>99	<1

a Values in parentheses refer to the synthesis in DMF solution.

b Content of LDL epimer, as determined by cHPLC.

c EtOAc was used as a liquid additive instead of DMF.

Despite the many examples in which stereochemistry is retained for short peptide synthesis, the possibility of epimerization remains a case-to-case risk, as illustrated by the four examples below.

After setting optimal conditions to perform amide synthesis using COMU or TCFH as coupling reagents (Scheme 35), Dalidovich et al.^110^ investigated the substrate scope revealing excellent yields with no remarkable epimerization of stereocenters in amino acids and (*S*)-naproxen, with the exception of the amide **114** obtained from (*S*)-ibuprofen (93–94% *ee*).

Santino et al.^64^ reported a mechanochemical peptide synthesis performing amidation using *N*-carboxyanhydrides (NCA) and hydroxyapatite as a base (Scheme 9). Although this method was successful in synthesizing several different peptides using different amino acids without appreciable epimerization and in good yields, performing the protocol with l-phenylglycine NCA and d-Val-OMe led to formation of a dipeptide with 93% yield and a 92:8 diastereomeric ratio.

A mechanochemical method to produce oligopeptides using TBTU as coupling reagent and Cs_2_CO_3_ as a base was developed by Wróblewska et al.^87^ and was evaluated for its tendency to epimerization (Scheme 23). Tetrapeptides Boc-Ala-Phe-Phe-Phe-OMe (87:13 *dr*), Boc-Phe-Leu-Phe-Phe-OMe (93:7 *dr*), Boc-Phe-Val-Phe-Phe-OMe (74:26 *dr*) and Boc-Phe-(D)­Ala-Phe-Phe-OMe (92:8 *dr*) were obtained in high stereoselectivity, but as mixtures of diastereomers. This outcome may be attributed to the strong basicity of Cs_2_CO_3_, which facilitates epimerization. In this context, the potassium *tert*-butoxide-mediated amidation of esters is particularly notable,^118^ as the use of this strong base led to complete racemization of the stereocenter in the _L_-proline-derived amide **133g** (Scheme 40).

Finally, a recent twin-screw extrusion method for the synthesis of di- and tripeptides from unactivated amino acids developed by El-Dine et al. showed varying epimerization ratio’s depending on the coupling reagent used (Scheme 34).^107^ Although the optimized procedure applying DIC with Oxymapure and AcOEt as liquid additive revealed dipeptide formation with excellent yields absent of epimerization, the screening of other coupling reagents and additives revealed epimerization (5.25% epimerization during mechanochemical synthesis of Fmoc-His­(Trt)-*S*-phenylethylamide using TCFH/DIEA).

To summarize, the majority of known mechanochemical methods for peptide synthesis demonstrate negligible epimerization of epimerization-prone α-stereocenters in amino acids. These include protocols based on ball milling, twin-screw extrusion, and even manual grinding. However, despite these favorable findings, the risk of epimerization remains an important consideration, as it is highly dependent on the choice of reagents and reaction conditions.

## 6 Amide Bond-Forming Reactions as a Model for Elucidation of Driving Forces of Mechanochemical Transformations

Understanding the fundamental driving forces that govern chemical reactivity under mechanochemical conditions, such as intense collisions, friction, shear, localized energy dissipation, and local pressure increases, remains a central challenge in the field. One of the complications in understanding mechanochemistry lies in the broad range of phenomena it encompasses, spanning from the scission of covalent bonds in polymers^185^ to chemical transformations in powdered solids, which are the focus of this review. In addition, powder-based mechanochemistry spans a wide range of reaction types and experimental techniques, with reaction kinetics governed by numerous process parameters that currently challenge rigorous physical chemistry formalism and are normally addressed through phenomenological models.^186–188^

This inherent complexity has sparked ongoing debates^42^ about whether specific transformations or mechanochemical techniques can be strictly classified as “mechanochemical” according to the classical IUPAC Gold Book definition.^41^ Indeed, in certain instances, the primary outcome of applying mechanochemical conditions appears merely to be enhanced mixing and macroscopic mass transfer, rather than chemical activation driven explicitly by mechanical force. In the context of amide bond formation, this is exemplified by successful amide couplings performed by manual mixing, such as CDI-mediated amidation achieved by spatula stirring^79^ or an EDC-mediated reaction facilitated simply with a glass rod.^103^

Moreover, reactions in powdered solids can be successfully modeled by postulating that the primary role of ball milling is to enable efficient mixing of solid reactants within “highly concentrated solid solutions”.^189–191^ Depending on the case, such systems may proceed through mechanisms closely resembling those in solution, though influenced by differences in the dielectric environment and without a pronounced contribution from mechanical force itself.^191^ Nevertheless, irrespective of the underlying physical rationale, it is evident that these conditions differ substantially from conventional solution-based reactions, and their interpretation still relies heavily on phenomenological models due to limited mechanistic understanding.

Given their practical importance and the broad range of developed synthetic methodologies, amide bond-forming reactions have primarily served as a rich source of empirical data, collected across diverse substrates, conditions, and mechanochemical platforms. These findings have helped to identify key factors influencing reactivity under solvent-free or solvent-deficient conditions. More rarely, amide bond formation has also been employed as a model system to probe mechanistic phenomena and elucidate the complexities of solid-state reactivity under mechanical stress. In such studies, the integration of synthetic experimentation with analytical techniques (both *in situ* and *ex situ*) as well as theoretical modeling, has begun to shed light on critical aspects such as energy transfer dynamics, the role of transient intermediates, and the influence of the physical milling environment.

Since the seminal work on amide^301^ and peptide synthesis,^59^ several crucial process parameters influencing reaction kinetics in the solid state have been firmly identified. For example, lower milling speed (such as reduced oscillation frequency in a VBM) results in slower reaction rates and decreased conversion. Similarly, increasing the milling load reduces conversion,^59,60^ and both dependencies can serve as indicators of the mechanochemical character of a transformation. Additionally, the initial work by Declerck et al.^59^ on UNCA-based peptide synthesis (Section 2.2.1) employed solid-state IR and ^13^C CP/MAS NMR to unambiguously confirm that peptide bond formation occurred entirely in the solid state, ruling out artifacts from workup procedures. This study also established apparent zero-order kinetics, consistent with a solid–solid reaction mechanism. Moreover, the material of the milling vessel and ball (e.g., steel, ZrO_2_, agate, PTFE, or rubber) has been identified as another critical factor, as most systematically demonstrated in studies on CDI-mediated amide synthesis (Section 2.2.3).^81^

Overall, the set of physical parameters governing the milling process, including milling speed, vessel dimensions, ball material and weight, and jar filling degree, collectively determine the mechanical energy input, which in turn dictates the observed chemical reactivity. A key challenge lies in navigating the interplay of multiple milling parameters to ensure reproducible outcomes, especially when transitioning between different types of ball mills. In 2024, Jafter et al.^128^ addressed this issue by developing a kinematic model, proposing that reaction progress is governed not merely by milling time or frequency, but by the cumulative mechanical energy transferred (*E*_*total*_) during the process. The latter can be calculated based on readily accessible inputs, such as dimensions of a milling vessel, mass of milling ball(s), milling speed and reaction time. Using three examples of *t*-BuOK-mediated amidation of esters (originally developed by Nicholson et al.^118^) as a model system, occurring with high, medium, and low yields (Scheme 71), they demonstrated that maintaining a constant *E*_*total*_ across different milling setups (such as a vibratory ball mill in the original study,^118^ and a planetary mill) enabled consistent and reproducible yields of amides **133a** and **102a**. To maintain a constant *E*_*total*_, three parallel reaction runs were performed for each amide using different milling speeds in a planetary mill, with the milling time adjusted accordingly. For the low-yielding synthesis of benzamide **135g** (16% yield in the original publication),^118^ the reproducibility was reasonably good (11% and 15% yield), as in one run the reaction showed a yield of only 4%, which could be caused by secondary effects that the models does not take into account (such as autocatalytic behavior, temperature effects, or altered reaction kinetics). Nevertheless, this finding supports the use of *E*_*total*_ as a practical and quantifiable descriptor linking multiple process parameters to chemical yields. The study also underscored that the impact energy per collision (*E*_*impact*_) must exceed a reaction-specific threshold (*E*_*threshold*_), highlighting the energies of individual impacts as another critical factor, especially at scale-up.

**Scheme 71:**
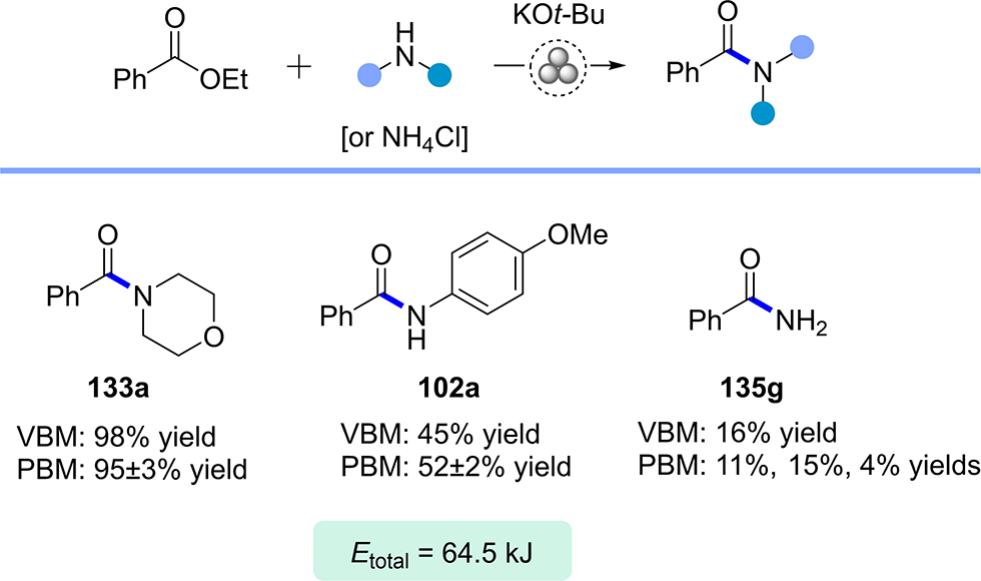
Experimental Validation of the Kinematic Energy Model Developed by Jafter et al.^128^

Another important characteristic of mechanochemical reactivity is that the chemical behavior of individual compounds is not governed solely by classical stereoelectronic factors typically emphasized in organic chemistry, but also by their physical properties, such as melting point and aggregation state. In particular, compounds that are liquid or possess lower melting points often exhibit enhanced reactivity, primarily due to improved mixing and molecular mobility. This phenomenon is well illustrated in the context of amide synthesis. As was already demonstrated in the study on ester amidation (Section 3.1.1, Scheme 40),^118^ the physical state of the ester had a significant influence on reaction outcome: under otherwise identical milling conditions, ethyl 4-bromobenzoate (a liquid) afforded amide **133b** in 80% yield, while the corresponding methyl ester (a solid) gave only 42% yield. Further illustration comes from the adaptation of the Beckmann rearrangement to reactive extrusion,^165^ where higher-melting-point substrates gave lower yields, likely due to impaired rheology hindering mixing.

It has also been proposed that the formation of a liquid or low-melting phase is a necessary feature of many mechanochemical reactions at some stage of the process.^192^ This hypothesis is consistent with observations of Ravalico et al.^65^ who identified DMAP as the most effective base in the acylation of amines with NHS esters, attributing its efficiency not only to its basicity but also to the more rapid liquefaction of the reaction mixture during milling. Wróblewska et al.^105^ provided significant mechanistic insight into the EDC-mediated coupling (Section 2.3.1, Scheme 32) using solid-state NMR and XRD. They showed that EDC exists in a stable cyclic form in the solid state, which is unreactive toward carboxylic acids. However, upon grinding with an acid, EDC undergoes ring-opening, accompanied by the formation of a low-melting intermediate phase essential for reactivity. This behavior is notably different from the same reaction in organic solvents.

The importance of a transient liquid phase in enhancing reactivity under mechanochemical conditions is also evident from numerous examples of amide synthesis employing LAG.^60,62,92,93,110,159,161^ Liquid additives were introduced to accelerate reaction kinetics,^60^ improve yields,^93^ facilitate material flow in reactive extrusion,^62^ and modulate rheological properties. Although the precise mechanistic role of LAG remains incompletely understood,^37,193^ the liquid phase may serve multiple, context-dependent functions, including acting as a molecular lubricant, promoting better mixing, or stabilizing reactive intermediates. Alternatively, inert solid additives such as NaCl and Na_2_SO_4_ have been employed to improve mixing efficiency in both ball milling^67^ and twin-screw extrusion processes.^120^

Mechanochemical conditions significantly enhance the reactivity of solid reagents that are typically unreactive in conventional organic solvents. In the context of amide bond formation, notable examples include the use of inorganic compounds such as hydroxyapatite,^64,99^ hydrotalcite,^98^ alkali metal carbonates,^59,60,67,87^ and phosphates^93,110^ as bases, as well as zirconium nitride^152^ as a reaction promoter. Importantly, these conditions can lead to mechanistic pathways that differ markedly from those in solution-phase chemistry. For instance, in mechanochemical amide couplings mediated by COMU,^110^ dipotassium phosphate (K_2_HPO_4_) was found to substantially outperform other inorganic and organic bases in terms of the yield of amide product **112** (Scheme 72). Mechanistic investigations revealed that K_2_HPO_4_ serves a dual function: it acts not only as a base but also as a precursor to reactive acyl phosphate species **A**. This dual role introduces a mechanistic pathway, which complements the classical activated ester mechanism involving intermediates **B** and **C**.

**Scheme 72:**
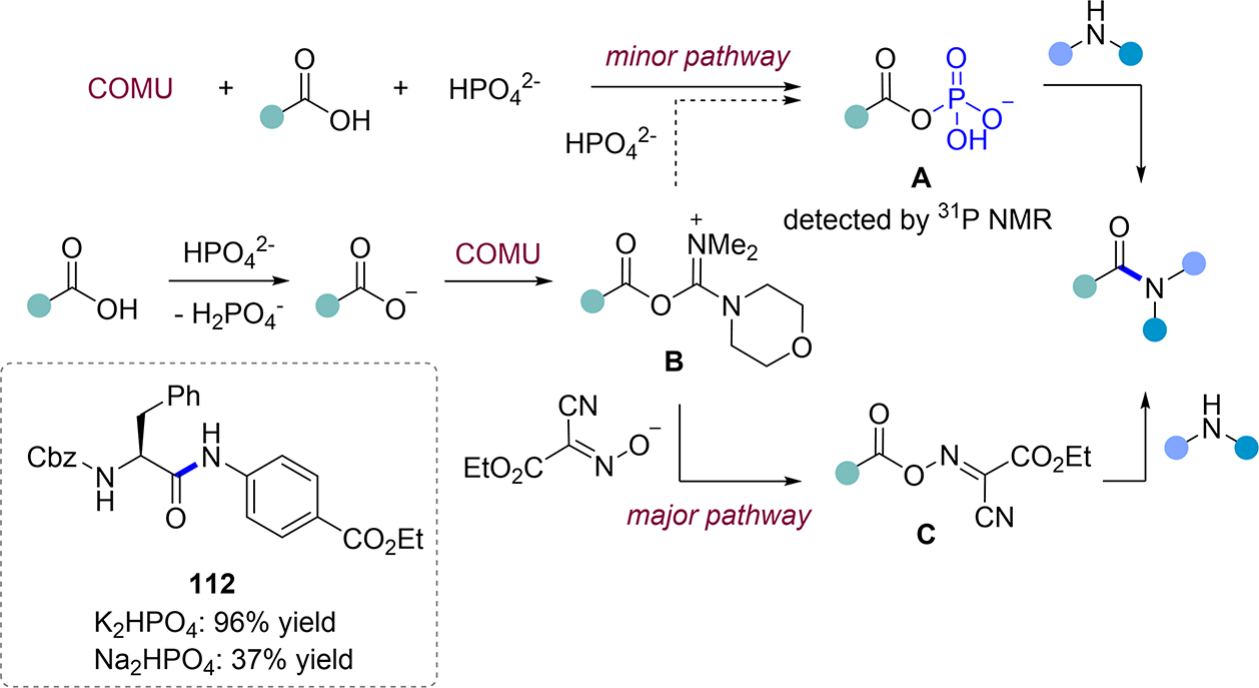
Role of Inorganic Phosphates in Promoting COMU-Mediated Amide Coupling

Notably, in this study, the corresponding sodium salt (Na_2_HPO_4_) proved significantly less effective, affording amide **112** in only 37% yield during optimization. This highlights a key aspect of ionic solid reactivity under mechanochemical conditions: the behavior of ionic species, such as inorganic salts, is strongly influenced by ion pairing in the solid state.^194^ Under solvent-free conditions, the reactivity of cations and anions is modulated by their counterions, with interaction strength often estimated via lattice energies or interpreted through the hard–soft acid–base (HSAB) theory.^195^ A further example of this principle is found in chloride-ion-templated macrocyclizations of oligopeptides,^114^ where tetraalkylammonium chlorides bearing weakly interacting cations, outperformed inorganic salts like KCl, NaCl, or CaCl_2_.

Wróblewska et al.^87^ investigated the mechanistic role of cesium carbonate in TBTU-mediated peptide coupling under ball milling conditions (Scheme 73). In this study, *N*-Boc-protected leucine was first milled with Cs_2_CO_3_ for 5 min in a ball mill, resulting in the transformation of the initial solid powder into a viscous, paste-like phase. TBTU was then added, and the mixture was milled for an additional 5 min, during which further phase changes were observed. The process was monitored using solid-state ^133^Cs NMR spectroscopy.

**Scheme 73:**
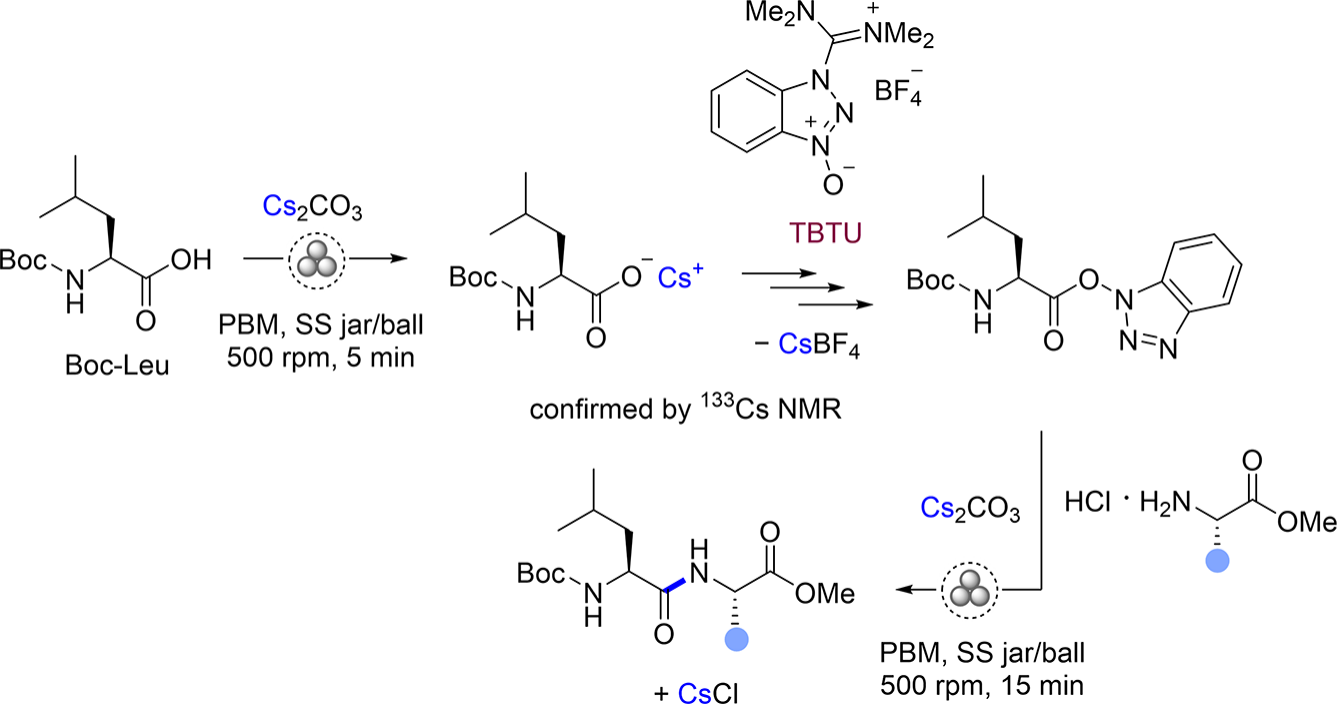
Role of Cesium Carbonate in Peptide Coupling with TBTU

These analyses confirmed the formation of the cesium salt of the amino acid in the first step, followed by its conversion into CsBF_4_ following the addition of the coupling reagent, indicative of successful activation. These results suggest that the cesium base enhances the nucleophilicity of the amino acid anion and the formation of CsBF_4_ as a driving force in the activation step. In the final step, addition of the amino acid methyl ester hydrochloride and further milling for 15 min led to the generation of CsCl, as confirmed by ^133^Cs NMR, completing the peptide synthesis.

Insight into the reaction intermediates involved in thermally promoted direct amide coupling was gained through *ex situ* synchrotron XRD analysis, as demonstrated in the study by Stolar et al.^49^ This study identified specific cocrystal salt intermediates formed between reactants, suggesting supramolecular preorganization plays a role even under combined thermal and mechanical stress. *In situ* Raman spectroscopy has also enabled continuous real-time monitoring of amide bond formation between 4-nitrobenzoyl azide and 1,4-diaminobenzene under various LAG conditions, revealing that the reaction is base-catalysed by the liquid additive.^318^

Several mechanistic proposals have also arisen from observing reaction outcomes under mechanochemical conditions. For example, Shibata et al.^86^ hypothesized that the base-free mechanochemical reaction between acyl fluorides (generated by using TFEDMA) and amines (Section 2.2.3) might proceed via trapping of the generated HF by the amide carbonyl oxygen, a plausible pathway specific to the concentrated, solvent-free environment. The observed lack of epimerization in many mechanochemical peptide coupling protocols (Section 5), particularly highlighted by Yeboue et al. using EDC/Oxyma,^97^ has also been mechanistically linked to the high-concentration environment suppressing the formation of epimerization-prone oxazolone intermediates, a distinct advantage over dilute solution conditions.

Although mechanistic pathways may differ in some cases from their solution-phase counterparts, most mechanochemical reactions follow analogous mechanisms. For example, studies on transition metal-catalyzed C–H amidation, supported by kinetic isotope effect experiments, have confirmed this mechanistic similarity.^142,144,145,149^

The influence of temperature on product selectivity in mechanochemical amidation was investigated by Cindro et al.^196^ (Scheme 74). Ball milling of acyl azide **193** with diamine **194** (2:1 ratio) at room temperature led exclusively to the monoamide product **195**, with no detectable formation of diamide **196**, even after 24 h. However, increasing the milling temperature to 40 °C enabled the formation of diamide **196** in 96% yield after just 4 h. Furthermore, a one-pot synthesis of the amide–urea compound **197** was achieved by initially milling **193** and **194** at room temperature, followed by heating the reaction mixture to 80 °C during continued milling. This temperature shift triggered the Curtius rearrangement of **193** and its subsequent reaction with **194**, as confirmed by *in-situ* Raman spectroscopy. Notably, when the mixture of **193** and **194** was preheated at 80 °C for 1 h before milling, the exclusive formation of diurea **198** was observed. These results demonstrate that precise thermal modulation during milling enables selective control over product formation (amide vs urea), providing access to structurally distinct products from the same reactants. In addition to selectivity control, temperature increase also significantly accelerated the reaction, offering synthetic possibilities not achievable by conventional milling or solution-phase methods.

**Scheme 74:**
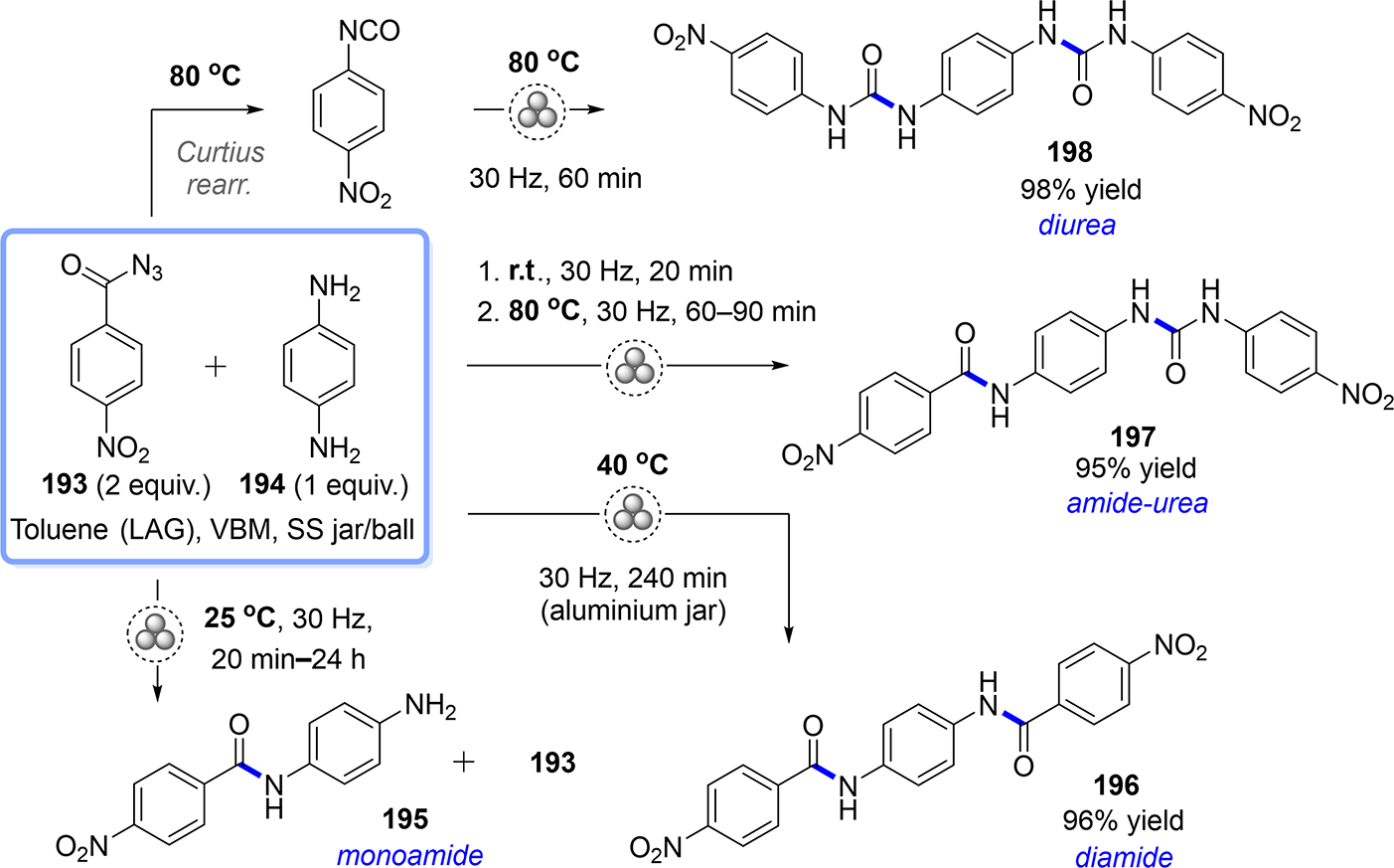
Selective Formation of Amide and Urea Derivatives by Thermally Controlled Mechanochemical Reactions

Stolar et al.^197^ demonstrated the significance of mechanochemistry in prebiotic peptide bond formation. The oligomerization of nonactivated glycine into linear oligopeptides (Gly_*n*_, *n* ≥ 2) was achieved via ball milling in the presence of mineral mediators such as TiO_2_ (anatase) in the absence of water (Scheme 75). TiO_2_ was selected due to the known high adsorption of glycine onto the mineral, although other prebiotically relevant minerals (SiO_2_, Montmorillonite K10) also catalyzed the reaction. Oligomerization of glycine to linear oligomers (*n* ≥ 2) was observed already at room temperature, along with generation of diketopiperazine **199**.

**Scheme 75:**
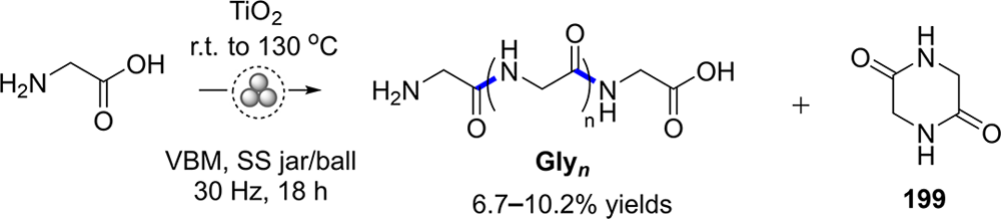
TiO_2_-Assisted Mechanochemical Peptide Bond Formation

Temperature played a critical role in promoting the polycondensation reaction: the highest combined yield of all oligomers (*n* ≥ 2) was 10.2% at 100 °C. Increasing the temperature to 130 °C led to the formation of longer oligomers (up to Gly_11_), although enhanced generation of diketopiperazine **199** was also observed. The reaction with 1.7 equiv of water as a LAG additive indicated tolerance of the oligomerization process to moisture at room temperature and at 130 °C. Notably, **199** was identified as a productive intermediate in the oligomerization process, rather than a “dead-end” species. Similarly, l-alanine afforded linear peptides up to Ala_5_, while a complex mixture of hetero-oligopeptides was produced using equimolar amounts of Gly and Ala.

Finally, the stability and activity of complex enzymatic systems under mechanical stress have been probed using mechanoenzymatic amide coupling (Section 4). Studies involving lipases (CALB) for kinetic resolution via amide formation^178^ or proteases (papain) for direct peptide synthesis^182^ demonstrated that these biomacromolecules can retain catalytic function under specific ball-milling conditions, although activity often diminishes upon recycling. The systematic study of the thermal and mechanical stability of CALB by Pérez-Venegas et al.^181^ ruled out degradation caused by mechanical forces and suggested that denaturation induced by organic solvents is a plausible deactivation mechanism.

In conclusion, amide bond-forming reactions have proven to be a versatile and informative model system for investigating the complex driving forces underlying mechanochemistry. Studies encompassing peptide couplings, direct amidations, and C–H functionalization have yielded critical insights into key process parameters governing energy transfer and reactivity. These include the role of reactive intermediates (such as acyl species, cocrystals), the physical state of reactants and low-melting phases, temperature effects, rheology, milling environment (including vessel wear and additive effects), and the stability of sensitive catalysts such as enzymes. Mechanochemical studies have even extended into prebiotic chemistry, further highlighting the breadth of this approach.

While notable progress has been achieved, particularly through the use of solid-state analytical techniques such as solid-state NMR, XRD and Raman spectroscopy, further *in situ* and *ex situ* monitoring and multiscale modeling efforts focused on amide-forming reactions will be essential to fully connect macroscopic energy inputs to the microscopic events driving chemical transformations, and clarify the role of mechanical force. Such understanding is crucial not only for advancing fundamental mechanistic knowledge, but also for enabling the rational design, scale-up, and reliable transfer of mechanochemical processes across different platforms and instruments.

## 7 Conclusions and Outlook

Since their emergence over the past few decades, mechanochemical methods for amide bond formation have evolved significantly, resulting in a rich and diverse array of synthetic strategies complemented by notable technological advances. This progress has been driven not only by the practical and structural importance of the amide bond in pharmaceuticals, agrochemicals, peptides, and advanced materials, but also by the inherent capability of mechanochemistry to minimize or entirely eliminate solvent use. This advantage not only avoids hazardous solvents such as DMF and DCM but also facilitates remarkably rapid reaction kinetics, significantly lower reaction PMI values, and streamlined workup and isolation, which in many cases can be achieved by direct filtration or crystallization from the milling medium, thereby reducing downstream processing and enabling process intensification, including integration with continuous mechanochemical processing.

A snapshot of the current methodological landscape is presented in Table 10. Mechanochemical approaches developed to date predominantly mirror classical solution-phase methodologies, particularly those involving activation of carboxylic acids. The field initially emerged with the use of activated carboxylic acid derivatives such as anhydrides,^301,302,58,72^ as well as UNCAs and NHS esters for peptide synthesis.^59,60,63^ Currently, methods utilizing coupling reagents such as EDC and CDI have become the most mature and widely applied strategies, offering broad substrate scopes and operational simplicity. EDC-based couplings are favored for their excellent functional group tolerance, minimal epimerization, and practical ease of operation. Similarly, CDI-mediated protocols enable robust two-step activations with broad applicability and convenient purification procedures. Importantly, these methods are unaffected by solubility constraints inherent to solution chemistry.

**Table 10:** Summary of the Key Mechanochemical Strategies for Amide Synthesis

Strategy	Equipment	Typical scale and maximal capacity demonstrated	Key highlights	Limitations	Estimated TRL reached
Thermal amidation	VBM, TSE	Gram-scale	High AE, excellent PMI ≈ 1, close to theoretical maximum	Scope limitations due to high temperature (>150 °C)	1–3
Activated esters and carboxyanhydrides	VBM, TSE	Gram- to decagram	Pioneering and well-developed methodology for peptide synthesis, including oligopeptides	Requires preparation of an activated ester or carboxyanhydride, optimized mostly for AAs	1–4
Acid chlorides and anhydrides	VBM, TSE	Gram- to decagram	Robust; quantitative yields for 2D polyamides and pigments at kilogram-per-day scale, complete solvent-free	Requires preparation of acid chloride; best suited to readily available acid chlorides or anhydrides	4–5
Amide coupling reagents	VBM, PBM, TSE, SSE	Gram-scale to ∼ 100 g (extrusion)	Wide substrate scope, low epimerization, mild reaction conditions, STY up to 4.7·10^6^ kg·m^–3^·day^–1^ (TSE), applications to API synthesis	Low AE, some coupling reagents necessitate prior activation of the carboxylic acid and may lead to byproduct formation	4–5
Amidation of esters	VBM, PBM, TSE	Gram-scale to ∼ 0.5 kg (TSE)	High AE, PMI < 2, 70 g·h^–1^ throughput (TSE), broad substrate scope, applications to API synthesis	Strong base (*t*-BuOK) limitations: racemization, base-induced side reactions, moisture-sensetive	4–5
Mechanoenzymetic methods	VBM, PBM, TSE	Gram- to decagram	Excellent enantioselectivity in kinetic resolutions, avoidance of coupling reagents in peptide synthesis	Enzyme cost, stability and recovery, peptide synthesis demonstrated only for natural AAs with hydrophobic side chains	1–4
Transamidation	VBM	Gram-scale	Transamidation driven by mechanical energy without catalysts and at room temperature	Demonstrated for phthalimides only	1–3
Ritter reaction	VBM	Gram-scale	Short reaction times (30 min) at room temperature	Side processes promoted by strong Brønsted acid catalyst (H_2_SO_4_)	1–3
Metal-mediated transformations, C–H activation and redox chemistry	VBM	Gram-scale	Significantly reduced reaction times compared to solution-based methods, unconventional routes to amides via nonstandard bond disconnections	Demonstrated mainly as proof-of-concept studies, often involving scarce metal catalysts	1–3
Leuckart reaction	TSE	Gram-scale	Short reaction times (5–10 min), STY 2.74 kg·L^–1^·h^–1^	Scope limitations due to high temperature (150 °C), aromatic aldehydes only	1–3
Rearrangements	VBM, TSE, bead mill	Gram- to decagram	100% AE, scalable synthesis of paracetamol (bead mill), STY up to 3.5 · 10^4^ kg·m^–3^·day^–1^ (TSE)	Currently limited to Beckmann rearrangement only, requires acid catalyst	4–5
MCR (Ugi and Passerini reactions)	VBM, TSE	Gram-scale (VBM) to 0.2 mol (TSE)	Short reaction times, complex scaffolds assembled in a single step	Isonitriles as starting materials, limitations in substrate scope	1–3

Alternative amide coupling reagents such as COMU, TCFH, and TFEDMA have been introduced to address specific limitations, particularly in the coupling of sterically hindered carboxylic acids or poorly nucleophilic amines. All these methodologies demonstrate improved green chemistry metrics, including reduced PMI and higher RME, when compared to benchmarking solution-based protocols. They also offer significant advantages such as shortened reaction times, simplified workup, and the ability to use inexpensive inorganic bases in place of tertiary amines.

Peptide synthesis, in particular, has seen substantial advancements through mechanochemical methods, enabling efficient sequential couplings for the construction of oligopeptides with yields comparable to traditional SPPS when utilizing EDC reagent in combination with Oxyma.^94^ Notably, peptide macrocyclization under highly concentrated mechanochemical conditions was also demonstrated and benefits from unique features such as chloride-anion templating.^114^ The peptide coupling reactions demonstrate excellent stereochemical fidelity by minimizing epimerization pathways such as oxazolone formation, likely due to the highly concentrated reaction environment.^97^

Moreover, to address the need to avoid waste-intensive processes involving activating groups and coupling reagents, direct condensation of carboxylic acids with amines has been demonstrated under elevated temperatures (>150 °C).^49,50^ This method achieves the highest atom economy and lowest reaction PMI values, approaching the ideal PMI = 1, which remains unattainable in solvent-based approaches.

Nonclassical strategies involving unactivated carboxylic acid equivalents (esters, lactones, nitriles, phthalimide) and amine surrogates (solid ammonia precursors, ammonium salts) effectively circumvent some limitations of conventional methods, such as reliance on low atom-economy activating agents or harsh reaction conditions. Additionally, transition metal-catalyzed C–H activation reactions rely on a different strategic bond disconnection and have been pioneered by utilizing Rh, Ir, Co, and Fe complexes, showing enhanced reactivity, reduced catalyst loadings, and milder conditions compared to solution-phase analogues. Redox-based transformations, rearrangements (Beckmann), multicomponent reactions (Ugi, Passerini), and even challenging C–F activations further underscore the versatility and synthetic breadth of mechanochemical amidation. Mechanoenzymatic strategies, employing enzymes such as lipases and proteases, have been explored and adapted for highly enantioselective kinetic resolution of secondary amines and peptide couplings under mechanical conditions, though enzyme stability and recyclability remain significant challenges.

However, many synthetic approaches well-established in solution chemistry remain unexplored under mechanochemical conditions, and thus offer promising avenues for future research (Figure 2). For example, organoboron catalysis,^198^ or other small-molecule catalysts^199,200^ for direct amide bond formation have not yet been demonstrated in a mechanochemical context. The closest related reports involve solvent-free conditions,^201–203^ which fall outside the scope of this review. Several other nonclassical strategies^9,204^ yet to be adapted to mechanochemistry include umpolung approaches, more diverse transamidation methods,^133^ carbonylation,^205^ and chemical ligation methods for peptide synthesis.^8^ Broadening the scope to incorporate these underexplored methodologies presents a promising direction for future development.

**Figure 2:**
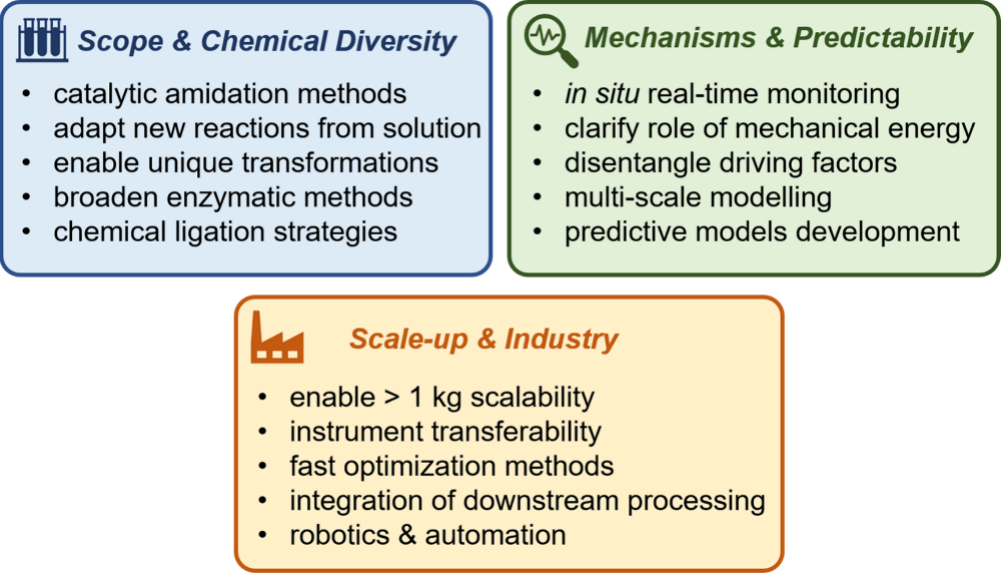
Promising avenues for future research.

In addition, there is a notable scarcity of amidation protocols that are truly unique to mechanochemistry and lack solution-phase analogues. The development of such unconventional methodologies could capitalize on distinctive features of mechanochemistry, such as the ability to activate otherwise inert inorganic solids, offering new solutions to long-standing synthetic challenges, such as enabling amide bond formation without the use of coupling reagents or high temperatures. Representative examples include the use of potassium hypophosphite (KH_2_PO_2_) with iodine^116^ as a mild coupling system and FeCl_3_-mediated amidation promoted by nanocellulose.^161^ Mechanochemical models of prebiotic peptide synthesis may also inspire the development of novel activation strategies.^197^

Importantly, the scalability of key mechanochemical methodologies has been validated, particularly through single- or twin-screw extrusion (TSE), for both pharmaceutically relevant peptides and APIs, reinforcing their potential for industrial-scale application. Notable examples include amide bond formation protocols using EDC, CDI, and DIC, as well as thermally promoted direct amide coupling, applied to such targets as moclobemide or aspartame. Among these, the *t*-BuOK-mediated amidation of esters stands out, having achieved production on a nearly half-kilogram scale via TSE.^120^ Nevertheless, the number of reports involving syntheses on a scale exceeding 10 g remains limited and warrants significant expansion. Further investigation into alternative scalable mechanochemical technologies is essential. Techniques such as bead milling,^166,206^ and resonance acoustic mixing (RAM),^207^ for which no examples of scalable amidation are yet reported, offer promising avenues for development.

To support broader industrial adoption, it is advisible to prioritize the development of scalable procedures for all mechanochemically efficient methods that have demonstrated high performance at the lab scale. In parallel, robust engineering solutions must be implemented to accommodate process intensification, including the integration of downstream operations such as extraction, crystallization, and purification. Minimizing reactor wear and preventing product contamination is critical, particularly for pharmaceutical manufacturing. Accordingly, quality-by-design principles and good manufacturing practice considerations should be addressed early, as mechanochemical processing can affect impurity profiles and particle/solid-state properties and may introduce trace wear-derived contaminants. These risks can be mitigated through appropriate equipment/material selection and routine monitoring of product quality and contamination. In addition, the development of fully automated robotic platforms for performing mechanochemical processes is highly desirable. This emerging field has the potential to greatly enhance reproducibility, scalability, and reaction optimization through precise force control.^208^

Another important aspect concerns the rational transfer of protocols between different types of mechanochemical equipment. This, along with successful scale-up, requires the identification of key process parameters and must be grounded in a deep understanding of the fundamental driving forces and reaction mechanisms. At present, empirically driven and practically oriented approaches are often prioritized over mechanism-based development, largely due to the still incomplete understanding of mechanochemical reaction pathways and the role of mechanical energy. Amide bond-forming reactions have served as convenient and practically relevant model systems in this context, suggesting cumulative energy input as a key parameter governing reaction yields.^128^

Although significant progress has been made in elucidating how mechanochemical transformations operate at the molecular level, such as the detection of intermediates and the study of reaction kinetics, a deeper mechanistic insight remains critical. To advance this understanding, continued development and application of *in situ* monitoring techniques, including real-time Raman spectroscopy and synchrotron XRD, are essential. These methods, combined with advanced computational modeling, will help to unravel reaction pathways, clarify the role of transient phases (such as eutectics and amorphous states), and quantify energy transfer dynamics. Moreover, further advances in predictive models that correlate milling parameters (such as frequency, duration, ball size and material, and temperature) with reaction outcomes remains a key objective. It is likely that such models will need to account for the physical properties of the reacting materials, including hardness, plasticity, particle size and morphology, aggregate state, rheological behavior, thermal conductivity and heat capacity (which influence mechanical and thermal energy dissipation), triboelectric charging, and other relevant parameters. Accounting for the combined influence of these factors makes the development of predictive mechanochemical models a complex and challenging task. Achieving precise control over the reaction environment, particularly with regard to temperature management inside the mill, will be vital for optimizing selectivity and enabling reactions involving sensitive substrates.

Given the early stage of the field and the limited understanding of the underlying driving forces in mechanochemical reactions, transparent reporting of all key experimental parameters is essential. These include factors that determine the cumulative mechanical energy transferred to the reactants,^128^ as well as any unsuccessful or null results. Reporting such negative outcomes, particularly for substrates that remain unreactive under mechanochemical conditions yet readily react in solution, is highly valuable for elucidating fundamental reactivity patterns under solvent-depleted conditions and mechanical stress. At present, the substrate scope in most studies is dominated by successful examples, often biased toward solid, readily available aromatic derivatives. Future reports should aim to present concise yet informative^127^ substrate screenings that also address aliphatic derivatives, sterically demanding or poorly nucleophilic substrates, epimerizable compounds, and molecules bearing sensitive functional groups. Furthermore, aspects specific to mechanochemistry, such as the influence of aggregate state (solid vs liquid), polymorphic form, and melting point differences,^209^ which may be critical for the formation of transient liquid or low-melting phases, should also be systematically evaluated.

The clear and consistent use of terminology is also essential to avoid inconsistencies that have appeared in the literature. Ongoing efforts by the IUPAC task group^210^ on “Terminology and Symbolism for Mechanochemistry” to develop standardized definitions and nomenclature will play an important role in establishing a common language, improving clarity in reporting, and supporting broader recognition and adoption of mechanochemical methodologies.

When it comes to greener synthetic methods, even though mechanochemistry inherently reduces solvent waste, future efforts should focus on minimizing or eliminating hazardous reagents or any additives, wherever possible. For example, LAG additives should preferably be chosen from among green and environmentally benign solvents.^211^ Developing efficient catalyst recycling strategies and designing protocols that simplify purification, for example, by enabling direct crystallization or precipitation from the mill, will further enhance sustainability. Moreover, expanding the range of catalysts and reagents specifically tailored for mechanochemical environments,^212^ particularly those based on earth-abundant metals, remains another important area for innovation. Enhancing stability and recyclability of enzymatic catalysts, broadening scope of mechanoenzymatic synthesis to accommodate more polar, charged, and structurally complex substrates, and integrating enzymatic steps with other mechanochemical methods represent additional promising research directions.

In summary, mechanochemical amide bond formation has firmly established itself as a powerful synthetic technique, distinguished by its strong alignment with green chemistry principles, high efficiency, time and resource economy, and operational robustness. Its innovative potential continues to drive progress across both academic and industrial settings. Future advancements in mechanistic insight, methodological innovation, reactor and process engineering will further solidify mechanochemistry as an essential platform in modern synthetic chemistry, extending its impact well beyond amide synthesis.

## +

Department of Chemistry and Materials Science, Aalto University, Kemistintie 1, 02150 Espoo, Finland

## Abbreviations

AA: Amino acid
Ac: Acetyl
AE: Atom economy
API: Active pharmaceutical ingredient
BDE: Bond dissociation energy
Bn: Benzyl
BO: Bayesian optimization
Boc: *tert*-Butyloxycarbonyl
BTEAC: Benzyltriethylammonium chloride
CALB: *Candida antarctica* lipase B
Cbz: Benzyloxycarbonyl
CC: Column chromatography
CDI: 1,1′-Carbonyldiimidazole
CEf: Carbon efficiency
cHPLC: High-performance liquid chromatography on a chiral stationary phase
COMU: (1-Cyano-2-ethoxy-2-oxoethylidenaminooxy) dimethylamino-morpholino-carbenium hexafluorophosphate
CuTC: Copper­(I) thiophene-2-carboxylate
DCE: 1,2-Dichloroethane
DCM: Dichloromethane
DEPBT: 3-(Diethoxyphosphoryloxy)-1,2,3-benzotriazin-4­(3*H*)-one
DIC: *N*,*N’*-Diisopropylcarbodiimide
DIEA: *N*,*N*-Diisopropylethylamine
DKP: Diketopiperazine
DM: Dyno-mill
DMAP: 4-Dimethylaminopyridine
DMEDA: *N*,*N*′-Dimethylethylenediamine
DMF: *N*,*N*-Dimethylformamide
DMI: Dimethyl isosorbide
DMTr: Dimethoxytrityl
DP: Degree of polymerization
EDC: 1-[3-(Dimethylamino)­propyl]-3-ethylcarbodiimide
Fmoc: 9-Fluorenylmethyloxycarbonyl
GA: Grinding auxiliary
GVL: γ-Valerolactone
HAp: Hydroxyapatite
HATU: (2-(7-Aza-1*H*-benzotriazol-1-yl)-*N,N,N’,N’*-tetramethylaminium hexafluorophosphate
HBTU: (Benzotriazol-1-yloxy­(dimethylamino)­methylidene)-dimethylazanium hexafluorophosphate
HDMB: 1-((Dimethylamino)­(morpholino))­methylene-1*H*-benzotriazonium
HFIP: 1,1,1,3,3,3-Hexafluoro-2-propanol
HOAt: 1-Hydroxy-7-azabenzotriazole
HOBt: Hydroxybenzotriazole
HT-S: Hydrotalcite
ICP-MS: Inductively coupled plasma mass spectrometry
LA: Liquid additive
LAG: Liquid-assisted grinding
LCA: Life-cycle assessment
MCR: Multicomponent reaction
ML: Milling load
NCA: *N*-Carboxyanhydride
ND: Nanodiamonds
NHS: *N*-Hydroxysuccinimide
NMI: 1-Methylimidazole
NMP: 1-Methyl-2-pyrrolidone
NP: Nanoparticles
Oxyma: Ethyl cyanohydroxyiminoacetate
PBM: Planetary ball mill
PDI: Perylene diimide
PG: Protecting group
PMI: Process mass intensity
PMMA: Poly­(methyl methacrylate)
PTCDA: Perylene-3,4,9,10-tetracarboxylic dianhydride
PTFE: Polytetrafluoroethylene
*p*-Ts-Im: 1-(*p*-Toluenesulfonyl)­imidazole
PXRD: Powder X-ray diffraction
RAM: Resonance acoustic mixing (mixer)
RME: Reaction mass efficiency
ROP: Ring-opening polymerization
RT: Residence time
SDS: Safety data sheet
SEM: Scanning electron microscopy
SPPS: Solid-phase peptide synthesis
SS: Stainless steel
SSE: Single-screw extruder
STY: Space time yield
Su: Succinimide functional group
TBTU: 2-(1*H*-Benzotriazol-1-yl)-*N*,*N,N’,N’*-tetramethylaminium tetrafluoroborate
TCFH: Chloro-*N*,*N*,*N′,N′*-tetramethylformamidinium hexafluorophosphate
TCT: 2,4,6-Trichloro-1,3,5-triazine
TEM: Transmission electron microscopy
TFEDMA: 1,1,2,2-Tetrafluoroethyl-*N*,*N*-dimethylamine
TPE-NH_2_: Tetraphenylethylene-NH_2_
Trt: Trityl
TRL: Technology Readiness Level
TSE: Twin-screw extrusion (extruder)
UNCA: Urethane-protected *N*-carboxyanhydride
VBM: Vibratory ball mill
WC: Tungsten carbide
XRD: X-ray diffraction

## Notes

Reference 50 has now been published in a peer-reviewed journal: Lavayssiere, M.; Bantreil, X.; Lamaty, F. Bayesian Optimization of Solvent-Free Thermal Amidation via Reactive Extrusion. *Chem. Eur. J.* **2026**, e70914. https://doi.org/10.1002/chem.70914
